# Identification of peptide epitopes in major antigens of *Yersinia pestis* from infected humans and immunised mice

**DOI:** 10.1016/j.ebiom.2026.106523

**Published:** 2026-10-29

**Authors:** Hebert Echenique-Rivera, Arnaud Simon, Jean-Louis Spadoni, Pavlo Holenya, Karsten Schnatbaum, Jean-Philippe Meyniel, Olifara Andriatefy, Minoarisoa Rajerison, Javier Pizarro-Cerdá, Christian Demeure, Matthieu Schoenhals, Jean-François Zagury

**Affiliations:** a Laboratoire Génomique, Bioinformatique, et Chimie Moléculaire, EA7528, Conservatoire National des Arts et Métiers (Cnam), Paris, France; b Université Paris Cité, Institut Pasteur Paris, Unite de Recherche Yersinia, CNRS UMR6047, F-75015, Paris, France; c Université Paris Cité, Yersinia National Reference Laboratory, WHO Collaborating Research & Reference Centre for Plague, FRA-140, Paris, France; d Unité d’Immunologie des Maladies Infectieuses, Institut Pasteur de Madagascar, BP 1274, 101 Antananarivo, Madagascar; e Unité Peste, Institut Pasteur de Madagascar, BP 1274, 101 Antananarivo, Madagascar; f Bioinformatics Team, Peptinov, Hôpital Cochin, 27 Rue du Fbg Saint-Jacques, 75014, Paris, France; g JPT Peptide Technologies GmbH, Berlin, Germany; h Life Sciences Department, AdvanThink (ISoft), Parc des Algorithmes, Bâtiment Euclide, Route de l’Orme, 91190, Saint-Aubin, France; i Université Paris Cité, Institut Pasteur Paris, Département d’Immunologie, Institut Pasteur, F-75015, Paris, France

**Keywords:** Epitope mapping, *Yersinia pestis*, Plague patient, Humans, Mice, Vaccine

## Abstract

**Background:**

*Yersinia pestis* (*Y. pestis*), the bacterial pathogen responsible for plague in humans, remains a public health threat, particularly in light of the recent emergence of antibiotic resistance strains.

**Methods:**

To better understand the immune response against plague and to identify potential targets for immunointervention, we performed an epitope mapping study of the IgG response against a library of peptides representing 18 *Y. pestis* proteins. The study included sera from 51 individuals who had recovered from plague, 26 uninfected human controls, and 20 sera from mice protected by a live-attenuated vaccine.

**Findings:**

Analysis of human sera revealed 32 reactive peptides distributed across eight protein antigens, whereas analysis of mouse sera identified 187 reactive peptides spanning all 18 proteins. Combined analysis of the human and mouse IgG responses to the 32 human-reactive peptides identified 27 B-cell epitopes located within YopD, YopB, LcrV, FyuA, Pla, Ail, Caf1, and PsaA. Of these, one epitope had previously been described as protective and another as immunodominant, whereas the remaining 25 represent immunodominant epitopes identified in this study.

**Interpretation:**

Interestingly, IgG antibody responses to selected YopD and YopB peptides were significantly stronger in pneumonic plague than in bubonic plague, suggesting that the distinct clinical manifestations of the disease may be associated with distinct underlying pathophysiological and molecular mechanisms. Our comprehensive mapping of linear IgG epitopes recognised by convalescent human sera, combined with prioritisation based on their predicted neutralising potential, provides a resource for future studies evaluating peptide-based diagnostics, vaccines, and monoclonal antibody therapies against plague.

**Funding:**

The project was supported by a grant from Agence Nationale de la Recherche (ANR).

**Research in context**

**Evidence before this study**

Plague, caused by *Yersinia pestis*, remains endemic in many regions of the world and represents a growing threat due to the emergence of antibiotic-resistant strains. Previous work characterising the humoural response to *Y. pestis* has focused on a limited number of well-known proteins, such as LcrV and F1 antigen, with broad serological profiling without specific B-cell epitopes. Available data on human immune responses are particularly limited, with only a few studies systematically examining epitope-level reactivity in convalescent individuals. Evidence from animal studies indicates that targeting some innate virulence factors or outer membrane proteins may contribute to protection. To contextualise the evidence available before the publication of this study, we searched PubMed from database inception to September 5, 2026, using combinations of the terms “*Y. pestis*” with “B-cell epitope”, “B cell epitope”, “linear epitope”, “humoural response”, “neutralizing epitope”, “neutralizing antibodies”, and “epitope”. We also screened the reference lists of relevant publications. A few previous studies have identified antibody-reactive epitopes in selected *Y. pestis* antigens, including LcrV, Pla, and Caf1. However, we found no previous study providing a comprehensive analysis of epitope specificity across a large panel of *Y. pestis* proteins.

**Added value of this study**

This study has leveraged our unique blood samples from convalescent humans available at Institut Pasteur of Madagascar and provides a comprehensive mapping of IgG linear epitopes recognised after the recovery from a natural plague infection in humans and/or after vaccination/infection of mice. We prioritised 18 *Y. pestis* proteins to map linear epitopes and identified hundreds of reactive peptides. By comparing human and murine datasets, our study has identified 27 shared B-cell epitopes across eight antigens. Two peptide epitopes were previously described and the other 25 represent immunodominant peptide epitopes observed in both human and murine humoural responses. We established a priority ranking of the 27 B-cell epitopes based on their predicted potential to function as neutralising epitopes. These findings expand the known epitope landscape of *Y. pestis* and identify targets that may be exploited for immunodiagnosis and the development of preventive or therapeutic strategies.

**Implications of all the available evidence**

The combined evidence from this study and with previous research suggests that linear B-cell epitopes across multiple *Y. pestis* antigens are associated with natural and vaccine-induced protection. The epitope reactive peptide map provides a resource that can support the rational design of peptide-based vaccines and targeted monoclonal antibodies, which is particularly important in the context of rising antibiotic resistance. The differential antibody responses to T3SS proteins (YopD, YopB, and LcrV) epitopes observed in bubonic and pneumonic plague raise intriguing questions about the mechanisms by which distinct host–pathogen interactions influence disease manifestations and associated immunological signatures. Taken together, these findings underscore the potential of epitope-level profiling to refine plague countermeasures and identify antigens of interest for the development of improved diagnostics or vaccines.

## Introduction

Plague, a notorious infectious disease caused by the bacterium *Yersinia pestis*, has exerted a profound historical influence as a major cause of pandemics. Likely originating in Central Asia, it spread to Europe, Africa, and eventually the Americas through three major pandemics spanning from ancient to modern times.^1^ Typically transmitted by flea bites, the disease is fatal without prompt antibiotic treatment. The disease manifests primarily in three clinical forms in humans: bubonic, pneumonic, and septicaemic plague. Rodents are the plague reservoir in nature. Advances in diagnosis and therapy have dramatically reduced the global incidence of plague. Nevertheless, outbreaks persist in endemic areas, particularly in developing regions such as Madagascar, which reports the majority of global cases annually.^2^

*Y. pestis* has evolved clonally from *Yersinia pseudotuberculosis*, an enteric pathogen typically causing mild gastrointestinal infections. This evolutionary transition involved both gene loss and horizontal gene acquisition, yet the two species remain genetically very close.^3^ Both harbour the pCD1 plasmid encoding a Type III Secretion System (T3SS), an essential virulence mechanism responsible for injecting effector proteins into host cells. The T3SS features the LcrV antigen at its distal tip, a critical virulence factor and one of the best-characterised targets for neutralising immune responses.^4^ On the other hand, acquisition by *Y. pestis* of two plasmids (pPCP1/pPla and pMT1/pFra) endowed the bacterium with unique virulence determinants. These include the surface protease Pla, which promotes tissue invasion and bacterial dissemination within the host^5^ and the F1 antigen, which forms an anti-phagocytic capsule-like layer surrounding the bacterial surface.^6^ The F1 antigen is a molecular signature of *Y. pestis* infection, and an efficient target for neutralising antibodies^7^ so that almost all candidate vaccines efficient against plague now include F1 as target of immunisation.^4^ F1 detection is also used to diagnose plague using rapid tests and ELISAs.^8^

*Y. pestis* remains a threat to public health for several reasons. First, owing to its extreme pathogenicity, it is classified among the most hazardous microorganisms with potential use as a biological weapon, thereby constituting a major biosecurity concern.^9^ Second, in endemic settings, delayed diagnosis and treatment may contribute to severe disease and mortality. Third, although plague remains treatable with currently available antibiotics, recent studies have documented the emergence of antibiotic-resistant *Y. pestis* strains.^10^ Furthermore, evidence indicates that strains lacking the F1 antigen can still retain full virulence and infect humans.^11^ Together, these factors highlight the urgent need for improved diagnostic tools for early detection, as well as alternative prophylactic and therapeutic approaches against plague. Vaccines or neutralising monoclonal antibody treatments are likely to be most effective when directed towards carefully selected targets. Relevant selection criteria for these targets may include antigen accessibility on the bacterial surface, functional involvement in virulence, or metabolic pathways, and molecular abundance, all factors that enhance the likelihood of effective neutralisation and immune clearance. Identifying such antigens capable of eliciting potent antibody responses through vaccination or suitable for targeting by monoclonal antibodies thus represents a major objective. Previous investigations of the humoural immune response have identified antibodies directed against the F1 antigen^12^ as well as broadly reactive antibodies against various surface molecules.^13^

In antibody–antigen interactions, conformational epitopes, which depend on the three-dimensional structure of the antigen, are far more frequently observed than linear epitopes.^14^ Several studies suggest that peptide-based arrays may still detect antibodies targeting conformational epitopes, as linear peptides can partially reproduce the physicochemical and spatial properties of native epitopes.^15^, ^16^ Such binding may occur with reduced affinity compared to intact antigen–antibody interactions.^15^, ^16^ This property makes epitope mapping approaches useful not only for identifying linear epitopes but also for providing an overview of serum immunoreactivity against protein antigens.

In the present work, we investigated *Y. pestis* antigens recognised by antibodies from patients convalescent from plague with the epitope mapping technology. We focused on 18 selected surface proteins of *Y. pestis*, specifically targeting regions likely to be exposed to antibodies in the external environment. Sera obtained from individuals recovering from plague in endemic areas of Madagascar were tested to determine which peptides were recognised by their IgG antibodies. To further augment this study, the analysis of sera from mice immunised with a live attenuated F1+ *Y. pseudotuberculosis* vaccine (VTnF1)^17^ and subsequently challenged with a fully virulent *Y. pestis* strain was also performed. The mouse vaccination–challenge model was used as a controlled system to evaluate antibody responses following exposure to whole *Y. pestis* antigens and to complement human serological analyses for identification of potential protective immune targets. This complementary approach enabled the identification of epitopes recognised by both humans and mice antibodies following exposure to whole bacterial antigens, resulting in a list of potential targets for developing peptide-based therapeutic strategies (vaccine or monoclonal antibodies) as well as improved diagnostic tools.

## Methods

### Biological samples

The human blood samples were collected in Madagascar by the Institut Pasteur de Madagascar and originated from 51 human individuals (bubonic *n* = 37, and pneumonic *n* = 14) with a documented history of plague infection. No septicaemic plague cases were reported in our study. Detailed information on the healthy control and convalescent plague patients included in our study is provided in Table S1. During plague outbreaks in endemic areas, the number of infected individuals is generally limited. During each sampling campaign, we systematically collected samples from all available infected individuals in the affected region, without applying selection criteria among patients. In this sense, the sampled cases can be considered representative of the documented outbreak, as all identified infected individuals were included. Because the number of cases per outbreak is typically low, the 51 patients included in this study originated from three independent sampling campaigns. Taken together, these cohorts can therefore be considered broadly representative of plague cases observed in these endemic settings.

The blood was collected three months after the episode of *Y. pestis* infection and plasma was frozen at −80 °C until analysis. Each plasma underwent a confirmation testing for the presence of anti-F1 antibodies by ELISA test. The selection of the three-month post-infection time point for human convalescent samples was intended to capture a mature antibody response enriched in higher-affinity and more stable antibodies. This timing may result in reduced overall antibody titres and a narrower detectable repertoire compared with earlier time points. Nevertheless, this approach may favour the detection of peptide epitopes that elicit higher-specificity antibody responses across the 18 antigens. The plasma of 26 uninfected participants, which were confirmed negative by ELISA for the F1 antigen, were used as negative controls. It was reasonable to assume that control individuals, originating from the same plague-endemic regions, were exposed to broadly similar environmental microorganisms as convalescent plague patients, with the exception of *Y. pestis*.

### Ethics

The collection of human blood samples was approved by the National Ethics Committee of Madagascar (ethics protocol numbers 086/MSNAP/SG/AMM/CERBM dated 27/07/2021 and 050/MSNAP/SG/AMM/CERBM dated 10/05/2022). All patients gave written informed consent and in cases of children, parents gave the authorisation. To ensure confidentiality, all samples were coded prior to use. The study involving human participants and human biological samples was conducted in accordance with the ethical principles of the Declaration of Helsinki.

Animal experiments were approved under the research protocol (APAFIS 180074) by the Institut Pasteur Ethics Committee for Animal Experimentation (CETEA) on behalf of French Ministry of Research. They were conducted in accordance with applicable national and institutional guidelines for animal care and use and were reported in compliance with the ARRIVE guidelines. No formal pre-registration of the study protocol was performed, and the study design and analysis plan were defined prior to initiation of the experiments.

### Bacterial strains and growth conditions

The vaccine strain VTnF1, derived from *Y. pseudotuberculosis* IP32953 used for immunisation, was previously described.^17^ This bacterial strain was genetically modified to express the F1 antigen (Caf operon), retain a functional Type III Secretion System and carry attenuating mutations such as the deletion of the High Pathogenicity Island and genes *psaA* and *yopK*.^17^ The VTnF1 strain was grown on LB agar plates at 28 °C overnight before immunisation, washed with saline solution, and normalised to the desired bacterial concentration. For the challenge of immunised mice, *Y. pestis* CO92 strain from a frozen stock (−80 °C) was grown on LB agar supplemented with hemin 0·002% for 48 h at 28 °C. Bacterial suspensions in saline for infection were prepared from the initial culture on LB agar-plates to the desired concentration of 10^3^ CFU in 50 μl by measuring OD_600nm_, and the actual content was verified by plating. CO92 is a ‘Orientalis’ *Y. pestis* belonging to the same 1.ORI phylogenetic branch as the *Y. pestis* found in Madagascar.^18^

### Animal housing, immunisation, and infection

Institut Pasteur animal facilities are accredited by the French Ministry of Agriculture (#B 75 15-01, 05/22/2008), in compliance with the French and European regulations on Laboratory Animals (EC Directive 86/609, French Law 2001-486 issued on June 6, 2001). Experiments were conducted in a BSL3 facility. OF:1 mice are outbred and were selected for our immunisation–challenge studies because they exhibit a more diverse immunological repertoire than inbred strains. A single sex (female) was used in this study to minimise potential confounding effects associated with mixed-sex housing. In previous vaccination studies no sex-related differences in immune responses or protective efficacy were observed.^17^, ^19^ The experimental unit is represented by a single animal. The experimental design and sample size were defined with the help of a statistician to allow for a paired longitudinal analysis with mice followed from naïve through vaccination and later challenge. This design allowed each mouse to serve as its own control, thus reducing the impact of inter-animal variability as a potential confounding factor. A power analysis based on Fisher's exact test indicated that a sample size of 20 mice per group would provide 97·5% power to detect a difference in survival between control mice (expected survival rate <5%) and vaccinated mice (expected survival rate >60%), assuming a Type I error rate (α) of 0·05. Furthermore, this sample size was considered adequate for comparisons between groups for the secondary quantitative endpoints. Blinding was not performed because all animals were housed randomly and all received the same treatment.

A total of 20 female OF1 mice, six weeks old (RRID:MGI:8245573; Charles River Laboratories) were used to collect sera at three different time points: before vaccination (“naïve”; day −7), after vaccination (“vaccinated”; day 21), and after challenge with *Y. pestis* (“vaccinated and challenged”; day 60). The experimental protocol in mice is shown in Figure S1. Mice were immunised by oral vaccination with the VTnF1 live plague vaccine candidate^17^ at the dose of 10^8^ CFU (colony forming units). The challenge with *Y. pestis* was performed 30 days post immunisation by subcutaneous (s.c., 50 μl) injection of CO92 (10^3^ CFU – 100× LD_50_) to mimic bubonic plague as previously reported (Figure S1).^17^ The primary outcome measure was survival following challenge with *Y. pestis*. Secondary outcome measures included serum IgG reactivity against *Y. pestis* peptides, assessed by high-density peptide array analysis.

All mice survived the challenge, and blood was collected 30 days post challenge which corresponds to the sera “vaccinated + challenged”. The samples of the three different time points (60) were kept at −20 °C until analysis. A group of seven naïve mice was also challenged in parallel with the vaccinated mice to ensure that the challenge was lethal, however, these animals were not used for serum collection. Therefore, a total of 27 mice was used in this study. No animal was excluded from the analysis, all had comparable behaviours and survival to vaccination and challenge. In accordance with institutional and ethical guidelines, predefined humane endpoints were applied (humane killing of mice losing 30% weight, or in a clear state of prostration with no reaction to stimuli, with ruffled fur and dirty eyes indicating strong fever). However, such endpoints were not reached because all naïve mice rapidly succumbed to challenge infection, as expected for this lethal model. As expected, vaccinated mice survived and did not show clinical signs of plague. No unexpected adverse events, such as abnormal clinical signs outside the expected course of infection, procedure related complications, or unintended mortality, were observed.

### Design of peptide array

The *Y. pestis* CO92 (GenBank: CP009973.1) was used as a reference strain for the study. The analysis of epitope mapping was performed taking as reference 18 protein sequences from major *Y. pestis* virulence factors and surface exposed proteins that may be structural proteins or have roles in bacterial metabolism (Table S2). The protein sequences were analysed, and the identification of transmembrane regions was performed using the online software SWISS-MODEL (RRID:SCR_018123; https://swissmodel.expasy.org/) for protein structures. To focus on epitopes accessible to antibodies, we excluded peptides predicted to be structurally inaccessible in intact bacteria. For each protein, peptides were defined as sequences of 15 amino acids long overlapping with the previous one by 11 amino acids to provide full linear coverage of the selected protein sequences.

### Peptide microarrays experiment

The peptide microarrays used for the epitope mapping experiment were designed and synthesised by the company JPT technology GmbH (Berlin, Germany) as previously described.^20^ The peptides were synthesised using SPOT synthesis method,^21^ cleaved and chemo selectively immobilised on glass slides. Each microarray glass slide contains each peptide in triplicate. The peptide library contained 1079 overlapping 15-mer peptides. Peptide array slides were incubated with samples diluted in buffer SuperBlock T20 (TBS) (Thermo Scientific #37536). For human plasma, (diluted 1:200) for 1 h at 30 °C and revealed with AlexaFluor647-labelled anti-human IgG (Jackson ImmunoResearch Labs Cat# 109-605-098, RRID:AB_2337889; 1 μg/ml). Mice sera (diluted 1:200) were similarly incubated for 1 h at 30 °C and slides were revealed with Goat Anti-Mouse IgG Fc (DyLight® 650-labelled; Abcam Cat# ab97018, RRID:AB_10679367; 1 μg/ml). Between each incubation, a washing step is added with 0·5% Tween-20 in 1× TBS buffer. After the last incubation, microarray slides are washed and dried into a microcentrifuge prior to scanning with the GenePix Autoloader 4300 SL50 (Molecular Devices, Pixel size: 10 μm) equipped with the GenePix Pro 7·0 analysis software (Molecular Devices; RRID:SCR_010969). Signal (Mean Fluorescent Intensities: MFI) ranged between 0 and 60,000. Each plasma was tested in three technical replicates in the same slide and signal values are the result of the mean from the triplicates. Each array allows one control and six test samples per slide, and batches of four slides were run for each experiment, for a total of six batches for human and four for mice samples. Personnel involved in animal handling, vaccination, challenge, and sample collection were aware of animal identities and sampling time points. Investigators performing the peptide array analysis received only coded numbers for human and mouse samples with no information regarding the subject or the timepoint ensuring that outcome assessment was conducted in a blinded manner.

### Statistics

Statistical analyses were performed using R (RRID:SCR_001905, version 4·3·1) within the software platform Amadea (AdvanThink/ISoft, Saint Aubin, France) and used as already described previously.^20^ Amadea is a no-code data science platform designed to facilitate high-performance data processing and transformation through a user-friendly, visual interface. Its proprietary Data Morphing® technology enables dynamic and intuitive data manipulation, while the underlying engine can process up to ten million rows per second and per core. No formal sample size calculation was performed; instead, all available sera fulfilling the inclusion criteria were included in the analysis (negative for healthy controls, and positive for convalescent in anti-F1 ELISA). The batch effect was removed using technical control spots (serum-free). For each control spot, we adjust the mean fluorescence intensity to a fixed value “*c*” (50 MFI for human data and 200 MFI for mice). This referenced value was chosen based on the lowest signal of the technical control spot. For each slide, the corrected MFI (mean fluorescence intensity) value of each individual peptide was calculated as *Batch-corrected MFI = raw MFI* − *(median (MFI control spot on the slide)* − *c)*. This process was applied to all the sera across the ten different batches. To account for peptide-specific baseline differences, responses for each peptide were centred by subtracting the 12·5th percentile of the corresponding response distribution across all samples.^22^ This robust normalisation reduces peptide-specific background while preserving the relative ranking of individual responses. The choice of the 12·5th percentile represents an empirical compromise between estimating the lower baseline response and avoiding excessive influence of extreme low values. Any resulting negative value was fixed to 1. Normality was assessed using Q–Q plots and the Shapiro–Wilk test. While some peptide distributions were approximately normal, others deviated from normality. Given this heterogeneity, both parametric and non-parametric tests were applied systematically to all peptide comparisons rather than selecting a test on a peptide-by-peptide basis.

For humans the data is composed of two groups: Healthy (*n* = 26) and Plague Convalescent individuals (*n* = 51, 37 from bubonic plague and 14 from pneumonic plague). For mice, the dataset is composed of 60 samples of sera from 20 mice collected at three different time points: Naïve, Vaccinated, and Vaccinated + Challenged. Statistical analyses were performed to compare two groups by pairs. To account for the variability and heterogeneity within the peptide datasets, we applied both parametric and non-parametric statistical tests.

For mice and humans, parametric (Student's *t*-test) were used to evaluate differences in group means, and non-parametric test (Mann–Whitney test for humans and Wilcoxon test for mice) were used as distribution-free complementary approaches. For Student's *t*-test and non-parametric analysis, to account for multiple testing, both Bonferroni (*p* < 0·0002) and false discovery rate (FDR) corrections (adjusted *p* < 0·05) were applied for each peptide comparison. When comparing two groups the analysis was performed as unpaired for humans and paired for mice. We implemented an additional analysis independently of distributional assumptions referred to as the rank-based approach (extreme responder) in which MFI values for each peptide were ranked in descending order. Peptides were selected as reactive peptides if the top 20% (15/77) sera high responders corresponded exclusively to the test group convalescent for the human dataset, or if the top 40% (16/40) corresponded to the vaccinated or vaccinated and challenged groups for the mice. This method complements classical statistical analyses by focussing on subsets of individuals with high responses rather than on differences in group central tendencies and is conceptually derived from outlier-based approaches such as COPA and Outlier Sum statistics.^23^, ^24^

The peptides identified from the three different analyses for each dataset were filtered and considered reactive peptides if a median value for each peptide was >500 MFI in the test groups. To facilitate comparison of immunogenicity across proteins of different sizes, reactivity percentage was defined as the proportion of reactive peptides among all peptides tested for each protein. This value corresponds to the number of reactive peptides (combining parametric, non-parametric, and extreme responders) divided by the total number of peptides tested. This normalisation reduces bias associated with protein length and provides a more balanced estimate of relative antigenic density. Boxplots were generated using the R library ggplot2 (RRID:SCR_014601, version 3·4·2) to visualise distributions and variability of reactive peptides between phenotypic groups.

Principal Component Analysis (PCA) was performed in Python Programming Language (RRID:SCR_008394, version 3·0) to visualise the structure and variability of data across the cohorts. Three-dimensional representation of PCA was performed to reduce the dimensionality of the peptide array matrix, which consisted of individuals (humans or mouse samples as rows) and either 1079 peptides or only the reactive peptides (as columns). All the PCA analyses were performed with the corrected datasets. Several PCA analyses were performed for humans: all peptides (1,079), all reactive peptides (32), the set of peptides tested in the study per single antigen, and the peptides identified as reactive for specific antigens. Group differences were tested using permutational multivariate analysis of variance (PERMANOVA test) with 999 permutations.^25^ For mice, PCA analyses were performed with all the peptides (1,079), and the set of peptides identified as reactive in the direct comparison naïve vs vaccinated + challenged. Before applying the PCA, data were standardised by mean centring using the StandardScaler function from the Scikit-Learn package (RRID: SCR_002577; version 1·5·2) in Python. Then, the PCA was applied with the same tool to generate three main principal components and their percentage of variance. For visualisation, three-dimensional plots were generated using Plotly (RRID:SCR_013991; version 6·0·1).

To investigate whether peptide reactivity differences were specifically associated with infection status rather than potential confounding factors, multiple linear regression analyses were performed in Python, using Pandas (RRID:SCR_018214. version 2·3·3) and statsmodel (RRID:SCR_016074. version 0·14·5) packages. Mean log_10_-transformed peptide reactivity values were used as the independent variables, whereas phenotypic group, age and sex were included as explanatory variables. Regression coefficients and corresponding *p* values were used to assess the contribution of each explanatory variable to the variability in peptide reactivity. The regression coefficients and associated *p* values were visualised as a forest plot generated using Matplotlib (RRID:SCR_008624. version 3·9·2).

### Identification of peptide epitopes

To identify linear peptide epitopes, the 32 reactive peptides (p) detected in humans were analysed together with their respective upstream (p −1) and downstream (p +1) overlapping peptides for significant reactivity in the comparison between healthy and convalescent individuals using a *t*-test. When adjacent peptides showed significant reactivity, the overlapping sequence was defined as a putative linear epitope. The epitope length was defined as 11 amino acids when two overlapping peptides were reactive and seven amino acids when three overlapping peptides were reactive (*p* < 0·05). When only a single peptide was significantly reactive, the entire peptide sequence was considered a putative epitope. For the identification of peptide epitopes in mice, the same set of peptides identified in humans was analysed for differential reactivity between naïve and vaccinated + challenged mice using a *t*-test (*p* < 0·05). The same criteria for epitope identification were applied. Human-derived epitopes were then used as reference sequences, and the sequence shared between the corresponding human and mouse epitopes was defined as the putative core epitope and highlighted by underlining the amino acid sequence.

### Mapping of epitopes in protein structures

To map the immunodominant epitopes identified, amino acid sequences were mapped onto the predicted 3D structure of each protein with the AlphaFold2 software^26^ using protein sequences from *Y. pestis* CO92 (shown in Table S2). The pLDDT (predicted Local Distance Difference Test) provides a per-residue estimate of model reliability, with values ranging from 0 to 100. Residues with pLDDT >90 are considered very high confidence, typically corresponding to well-resolved and stable structural elements. Scores between 70 and 90 indicate reliable backbone geometry but lower side-chain accuracy. Regions with pLDDT <70 often correspond to flexible loops, intrinsically disordered segments, or areas where the prediction is uncertain^26^ and described for structure interpretation in the AlphaFold Protein Structure Database.^27^ The experimental crystal structure of the *Y. pestis* Caf1M-Caf1 complex was retrieved from the Protein Data Bank (PDB ID: 1P5U; resolution 1·99 Å, RCSB PDB; RRID: SCR_012820).^28^ The structure corresponds to the X-ray diffraction model of the ternary Caf1M:Caf1:chaperone–subunit complex. Visualisation of molecular surface rendering and epitopes were performed using PyMOL (RRID:SCR_000305; version 2·5·5; https://www.pymol.org/).

For membrane-associated proteins, the predicted epitopes accessibility was evaluated according to their membrane localisation. The proteins sequences were queried against the SWISS MODEL repository to verify available structures that includes membrane positioning. The amino acids residues were manually inspected to determine whether they were located within transmembrane segments or exposed outside the membrane. Only solvent-accessible residues located outside the membrane were selected as antibody-accessible amino-acids. To estimate the solvent exposure and surface accessibility of reactive peptide regions, the Solvent-Accessible Surface Area (SASA)^29^ of each selected epitopes was calculated in PyMOL using the *get_area* function. To enable comparisons between epitopes of different length, the SASA value was normalised by the number of amino acids composing each epitope, resulting in an “mean SASA per residue” expressed in Å^2^ (square ångströms) per residue.

### Role of funders

The funders of the study had no role in the design, data collection, data analysis, data interpretation, or writing of the report.

## Results

### Selection of protein candidates for linear epitope mapping

To perform the linear epitope mapping, we used a peptide array technology. Eighteen proteins from *Y. pestis* strain CO92 were selected based on surface exposure and documented roles in virulence or metabolism (Table S2; Figure S2) for the linear epitope mapping. The proteins comprise the Type III secretion system (T3SS) related proteins (YscF, LcrV, YopD, and YopB), the subunit of the Psa/pH6 fimbriae (PsaA), F1 antigen proteins (Caf1 and Caf1M), and tubular proteins anchored in the outer membrane (Pla, Ail, OmpA, OmpF2, OmpX, OmpT, OmpC, and YadC), and some of them directly related to metabolism (Maltoporin850, FyuA and FhuE). Protein sequences were trimmed to remove putative transmembrane regions. The final sequence and number of peptides used for each protein are reported in Table S3: altogether, a total of 1079 overlapping peptides covering the 18 proteins were included in the study. Detailed information on antigen selection, sequence processing and peptide design is provided in Supplementary Results.

### Mapping of the humoural response (IgG) against *Y. pestis* antigens from plasma of plague individuals

Plasma samples were obtained from plague-convalescent individuals. Clinical plague manifestations included bubonic plague (*n* = 37) and pneumonic plague (*n* = 14). Blood sampling was performed three months following diagnosis. A group of healthy individuals with no known history of plague (26 Antananarivo blood donors) was also recruited and served as negative controls (Table S1). For both groups, plasma was tested by F1 antigen ELISA and Western Blotting for reactivity against the F1 antigen (data not shown). Healthy donors were all negative for F1 IgG by serology and plague survivors were all positive. Peptide microarray was used to assess IgG reactivity against each of the 1079 peptides and the IgG responses were compared between uninfected and plague samples. For this analysis, patients convalescent from pneumonic and bubonic plague were grouped together under “Convalescent” and analysed using three complementary statistical approaches (non-parametric Mann–Whitney test,^30^ and the parametric Student's *t*-test,^31^ with Bonferroni corrections^32^). In addition, an “extreme responder” analysis was also evaluated for each peptide (Fig. 1; Methods).

**Fig. 1:**
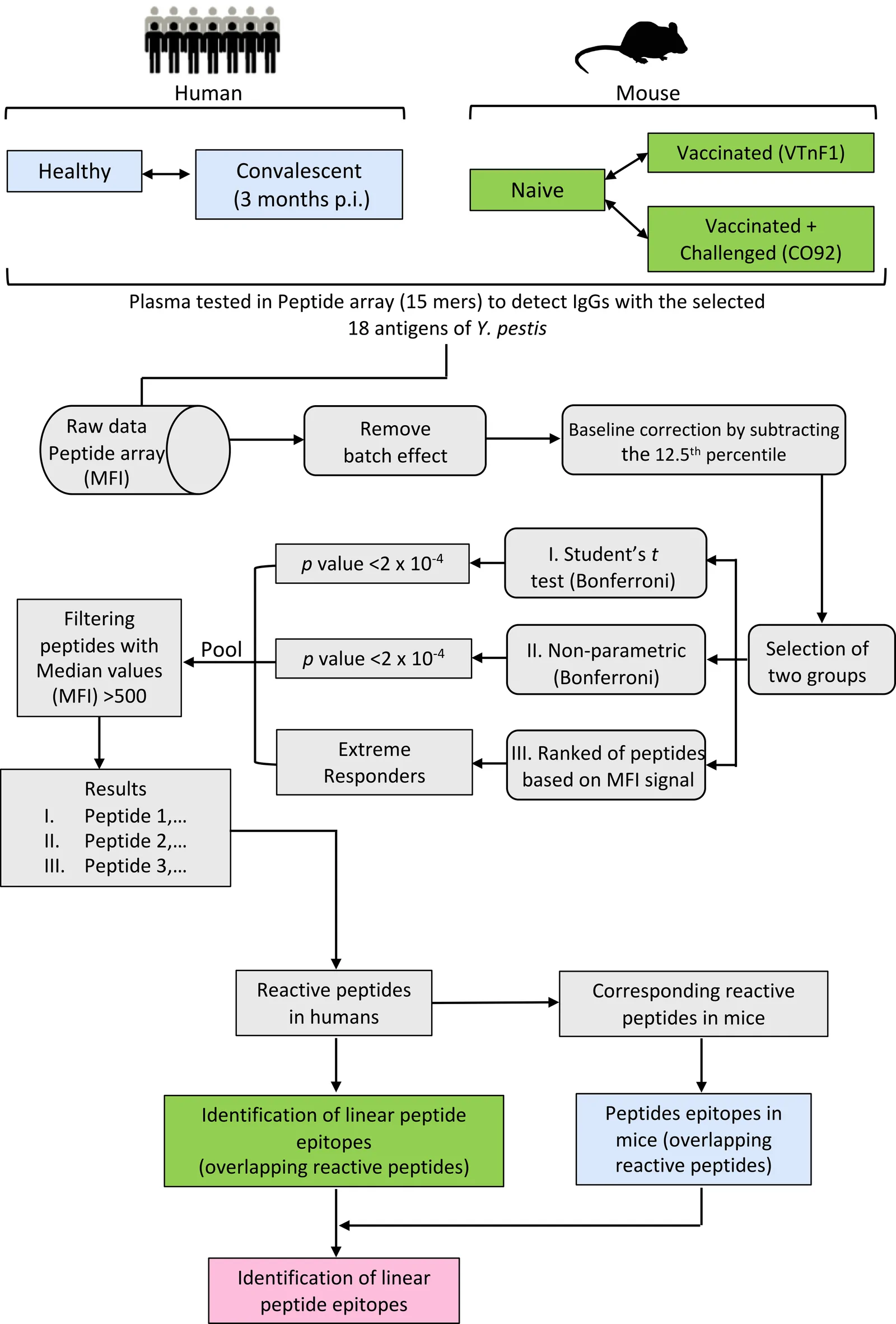
**Diagram describing the pipeline for the epitope mapping and identification of B cell epitopes against *Y. pestis* antigens**. Plasma samples were collected from healthy individuals (*n* = 26) and convalescent plague patients with confirmed *Y. pestis* infection (*n* = 51). Samples were screened for reactivity against the F1 antigen using ELISA and Western Blotting. In parallel, a mouse model of protection against *Y. pestis* was used to identify potential protective epitopes induced by the VTnF1 vaccine (live attenuated *Y. pseudotuberculosis* containing F1 antigen). Twenty mice were vaccinated on day 0 and challenged with *Y. pestis* CO92 on day 30. Human plasma and mouse sera were analysed using a peptide microarray containing overlapping 15-mer peptides spanning 18 selected *Y. pestis* antigens. Peptide array signals (mean fluorescence intensity, MFI) were processed using bioinformatic and statistical analyses to identify reactive peptides showing significantly higher responses than the corresponding control groups (healthy individuals for the human cohort and pre-vaccination sera collected on day −7 for the mouse cohort). For the non-parametric analyses (II), the Mann–Whitney test was used to compare two human groups, whereas the Wilcoxon signed-rank test was applied to the paired mouse samples. These analyses were complemented by a rank-based approach, in which peptides were also considered reactive if they met the predefined criteria for extreme responders (see Methods). Linear peptide epitopes were delineated by identifying overlapping positive peptides (*p* < 0·05) in the human dataset and were similarly evaluated in the mouse dataset. Linear epitopes identified in humans were then compared with those detected in mice. When overlapping epitopes were observed in both species, their shared sequence was defined as the putative core epitope. Arrows with two heads ←→ indicates direct comparisons between the groups for the analysis. p.i.: post infection. MFI: mean fluorescence intensity.

By combining the three methods, we found 32 peptides corresponding to eight proteins specifically recognised by plasma from convalescent individuals compared to controls (Table 1). For each of the eight reactive proteins, we computed a reactivity percentage (defined as the proportion of peptides found to be reactive among those tested for each protein) to rank them according to their linear peptide reactivity (Table 1): YopD (9·5%, 7/74) > LcrV (8·8%, 7/79) > PsaA (7·7%, 2/26), YopB (7%, 6/86) > Pla (5·7%, 4/70) > Ail (5·5%, 2/36) > Caf1 (2·9%, 1/35) > FyuA (2·5%, 3/118). This metric, based on reactivity percentage, allows a simplified comparison of protein immunogenicity and facilitates a global comparison between human and mouse responses.

**Table 1:** Peptide analysis of IgG response to *Y. pestis* antigens in humans and mice groups.

Antigens				Human (Healthy vs Convalescent)					Mouse (Naïve vs Vaccinated + Challenged)				
Protein name	Length (aa)	Experimental size of protein (aa)	Peptide number in Array (15 aa each)∗	Mann Whitney (Bonferroni *p* < 0·0002)	Student's *t*-test (Bonferroni *p* < 0·0002)	Extreme responder	Combination of peptides	Reactivity (%)	Wilcoxon (Bonferroni *p* < 0·0002)	Student's *t*-test (Bonferroni *p* < 0·0002)	Extreme responder	Combination of peptides	Reactivity (%)
YscF	87	78	17	0	0	0	0	0	3	0	0	3	17·6 (3/17)
YopB	401	355	86	3	1	5	6	7 (6/86)	20	2	5	20	23·3 (20/86)
YopD	306	306	74	5	4	4	7	9·5 (7/74)	20	2	7	20	27 (20/74)
LcrV	326	326	79	2	3	6	7	8·8 (7/79)	25	0	0	25	31·7 (25/79)
Caf1	170	149	35	0	0	1	1	2·9 (1/35)	6	0	0	6	17·1 (6/35)
Caf1M	258	233	56	0	0	0	0	0	18	11	4	18	32·1 (18/56)
PsaA	158	114	26	1	1	2	2	7·7 (2/26)	10	1	0	10	38·5 (10/26)
YadC	615	495	121	0	0	0	0	0	2	1	0	3	2·5 (3/121)
Ail	179	155	36	0	0	2	2	5·5 (2/36)	14	1	0	14	38·9 (14/36)
OmpX	174	127	29	0	0	0	0	0	3	1	0	3	10·3 (3/29)
OmpA	353	100	23	0	0	0	0	0	1	0	0	1	4·3 (1/23)
Pla	312	291	70	0	2	3	4	5·7 (4/70)	19	2	0	20	28·6 (20/70)
OmpT	311	214	51	0	0	0	0	0	5	5	1	5	9·8 (5/51)
OmpC	371	185	44	0	0	0	0	0	4	1	0	4	9·1 (4/44)
OmpF2	371	227	55	0	0	0	0	0	4	1	2	4	7·4 (4/54)
Maltoporin850	419	182	43	0	0	0	0	0	5	3	0	5	11·6 (5/43)
FyuA	673	481	118	1	1	2	3	2·5 (3/118)	14	0	1	14	11·9 (14/118)
FhuE	753	473	116	0	0	0	0	0	12	6	0	12	10·3 (12/116)
Total			1079	12	12	25	32		185	37	20	187	

The number of reactive peptides identified for each antigen for study group is shown. In the human cohort, peptide reactivity was compared between Healthy control (*n* = 26) vs Convalescent plague patients sampled three months after infection (*n* = 51). In the mouse cohort (*n* = 20), IgG responses were compared between the groups naïve vs vaccinated and challenged. For each antigen, the number of reactive peptides identified by each of the three statistical analyses is reported separately for the human and mouse datasets. The combined number of unique reactive peptides identified across the three analyses is also provided for each dataset. For both datasets, the percentage of reactivity (“Reactivity (%)") was calculated for each protein as the ratio of the total number of unique reactive peptides identified by the three analyses to the total number of peptides representing that protein in the peptide array (∗). aa, amino acids.

To assess the discriminatory power of the 32 identified peptides, their mean fluorescence intensity (MFI) values were summed for each individual, and the resulting composite reactivity scores were compared between the Healthy and Convalescent groups (Fig. 2A). A *t*-test revealed a statistically significant difference between groups (*p* = 1·69 × 10^−11^). This analysis indicates that these immunodominant peptides may contribute to a serological signature associated with plague infection. Interestingly, among the most immunogenic proteins identified by human IgG in the peptide array, we find those composing the tip of the T3SS (LcrV) and the translocon components (YopB and YopD). The signal obtained for each of the 32 peptides in the control and plague groups are shown in Figure S3. The same analysis was performed using false discovery rate (FDR) correction (Table S4A), identifying the same 32 peptides detected using Bonferroni correction (Table 2), along with 11 additional peptides (Table S4B).

**Fig. 2:**
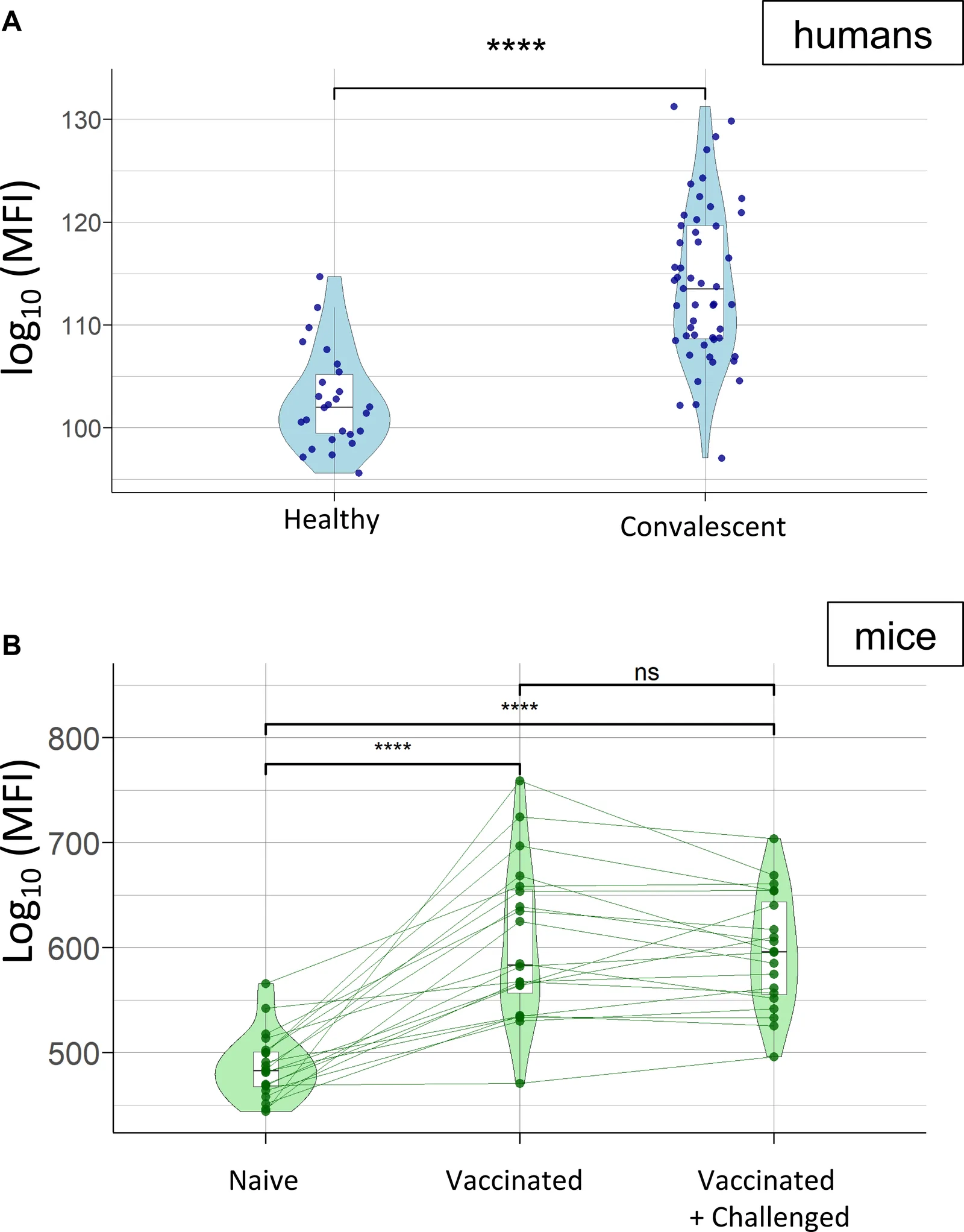
**IgG response to *Y. pestis* antigens in the peptide array**. **A)** Comparison of serum IgG responses between healthy controls (*n* = 26) and convalescent plague patients (*n* = 51; pneumonic and bubonic forms). Each data point in the box plot represents one individual and corresponds to the sum of log_10_ (MFI) values obtained against the 32 reactive peptides identified in the human cohort (and presented in Table 1). **B)** Comparison of serum IgG responses in mice from the protection model against plague. Sera were collected at three time points: day −7 (naïve), day 21 after vaccination with VTnF1 (vaccinated), and day 60, corresponding to 30 days after challenge with *Y. pestis* CO92 (vaccinated and challenged). Each data point represents one mouse and corresponds to the sum of the log_10_ (MFI) values obtained for the 187 reactive linear peptides identified by comparing naïve and vaccinated + challenged mice. These peptides are distributed across all 18 antigens included in the peptide array (Table 1). Statistical analyses between groups were performed using Student's *t*-test with significance defined as ∗∗∗∗, *p* value < 0·0005; ns: not significant. Human samples were analysed as unpaired data, whereas mouse samples were analysed as paired data. In each violin plot, the horizontal black line indicates the median, the box represents the interquartile range (IQR; 25th–75th percentile). MFI: mean fluorescence intensity.

**Table 2:** Linear peptide epitopes identified in *Y. pestis* from the analyses in humans and in vaccinated and challenged mice.

This table presents the 32 peptides specifically recognised by IgG in sera from convalescent plague patients, corresponding to eight *Y. pestis* proteins. In the blue section, the amino acid sequence is provided for each peptide, with residues highlighted in red indicating overlap with adjacent peptides also identified as positive in the same comparison (healthy vs convalescent; Student's *t*-test, *p* < 0·05). The number of overlapping peptides is indicated, together with the position of any additional positive peptide, when applicable (preceding peptide: p−1; following peptide: p+1). Corresponding linear epitopes identified in mice (comparison of naïve vs vaccinated and challenged animals; Student's *t*-test, *p* < 0·05) are shown in the green section of the table. The final (red) section presents the linear peptide epitopes identified in humans. Amino acids shared with the corresponding mouse epitopes are underlined and define the putative core epitope. Further details regarding peptide epitope identification are provided in Table S6. Pep = peptide.

### PCA of reactive peptides distinguishes clinical manifestations of plague in humans

A Principal Component Analysis (PCA) was used to assess whether some peptide sets could distinguish the different study groups. In the PCA with all peptides (1,079), individuals from the three groups (controls, bubonic, and pneumonic) clustered closely, showing little separation (Figure S4A). However, when the analysis was restricted to the 32 identified peptides, the PCA clearly separated a dispersed cluster of convalescent patients (both bubonic and pneumonic) from the compact cluster of healthy controls (Figure S4B), with the observed group separation supported by PERMANOVA (all peptides *p* = 0·01; 32 peptides *p* = 0·001).

For YopB (Figure S4D) and YopD (Figure S4E), PCA of the reactive peptides revealed distinct patterns differentiating the two types of infection. This observation was further supported by violin plot analyses (Figure S5) and by Table S5A, which showed that YopD and YopB were the only antigens containing peptides with stronger reactivity in pneumonic than bubonic patients (Figure S5B, *p* = 0·0033; Figure S5C, *p* = 0·049). These findings suggest that reactive peptides derived from YopD and YopB could represent candidate markers for distinguishing between bubonic and pneumonic plague, pending validation in larger cohorts.

### Mapping of the IgG response against *Y. pestis* antigens in a mouse vaccination-challenge model

In parallel with the human response, it was interesting to study the epitopes identified in an independent experimental mouse model. Mice were vaccinated with the live attenuated *Y. pseudotuberculosis* strain IP32953-vaccine VTnF1, which induces both humoural and cellular immune responses and confers protection against fully virulent *Y. pestis* in a bubonic plague challenge model.^17^ The vaccinated mice were subsequently challenged with *Y. pestis* CO92, and the collected sera were profiled using the same peptide arrays.

IgG reactivity was analysed in a similar manner as for human samples using the three complementary statistical approaches (see Methods) (Fig. 1). The comparison of the two groups (naïve vs vaccinated + challenged) identified 187 reactive peptides (Table 1). Proteins with the highest proportions of reactive peptides included Ail (38·9%, 14/36), PsaA (38·5%, 10/26), Caf1M (32·1%, 18/56), LcrV (31·7%, 25/79), Pla (28·6%, 20/70), YopD (27%, 20/74), and YopB (23·3%, 20/86) (Table 1). To evaluate the discriminatory capacity of the 187 peptides, MFI values were summed for each mouse and compared statistically between groups (*p* = 6·86 × 10^−8^) (Fig. 2B). Similar results were obtained when comparing naïve vs vaccinated groups (see Supplementary Results).

### Analysis of IgG-reactive peptides in humans and mice highlight potential linear B-cell peptide epitopes

Using the 32 reactive peptides identified in humans as a reference, we defined 27 peptide epitopes based on clusters of adjacent significantly reactive peptides (Table 2). Analysis of the corresponding peptide set in mice showed that only three of the 32 human-reactive peptides lacked supporting reactivity in mice (Table S6). Combined analysis of the human and mouse IgG responses showed that 31 of the 32 reactive peptides were supported by significant reactivity of adjacent peptides (*t*-test, *p* < 0·05), providing evidence for the corresponding linear peptide epitopes. Because some of the 32 reactive peptides were adjacent to one another, this integrative analysis ultimately resulted in 27 distinct peptide epitopes, with putative core epitopes delineated by the overlapping sequences shared between the human and mouse peptide epitopes (Table 2 and Table S6). The IgG responses to the 32 human-reactive peptides with the identified peptides epitopes are presented in Figure S3. The corresponding analysis of mouse IgG responses to these peptides, including reactivity to the upstream and downstream peptides (*t*-test, *p* < 0·05) used to define the epitopes, are shown in Table S6 and Figure S7.

### Mapping immunodominant epitopes onto 3D structural models

To evaluate the accessibility of the identified peptide epitopes to antibodies, we mapped them onto predicted 3D protein structures generated using Alpha Fold2 (Fig. 3). The three antigens with the highest number of mapped epitopes were LcrV (seven epitopes; Fig. 3A), YopB (five epitopes; Fig. 3B), and YopD (six epitopes; Fig. 3C), all components of the T3SS. For these three proteins, the monomeric AlphaFold models were predicted with low confidence. Nevertheless, given their roles in the assembly of the T3SS translocon complex and their localisation at the bacterial surface, the identified epitopes remain of particular interest.^33^, ^34^, ^35^

**Fig. 3:**
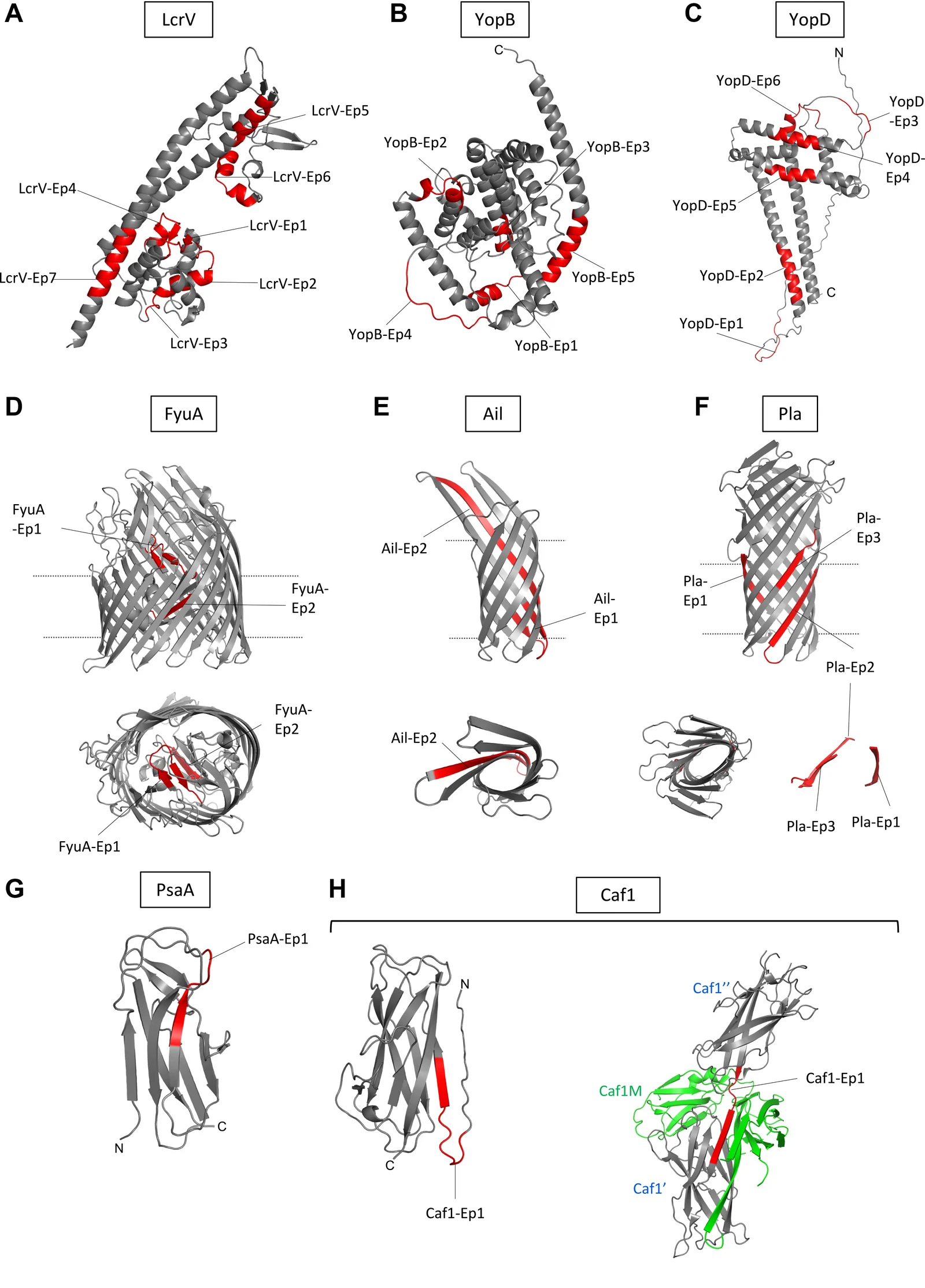
**Mapping of epitopes identified in the peptide arrays onto 3D structures**. The predicted protein structures were built from sequences of *Y. pestis* strain CO92 using the PyMOL tool based on the predicted structures obtained from Alpha Fold2. The figure shows the mapping of peptide epitopes identified in the present study (Table 2). For each protein the epitopes are highlight in red colour. Proteins related to T3SS (**A**–**C**), anchored membrane proteins (**D**–**F**), subunit of fimbriae (**G**), and Caf1 subunit of F1 antigen (grey) or associated with Caf1M (green) (**H**). The experimental crystal structure of Caf1-Caf1M was retrieved from the Protein Data Bank (PDB ID: 1P5U, 1·99 Å). The structure was visualised using PyMOL. Dotted lines in the structure models (**D**–**F**) indicate the protein regions that are predicted to be inserted in the bacterial outer membrane. The confidence scores for predicted structure (pLDDT score) of the proteins in panels A to H are shown in Table S2. For panel H (right side, Caf1M:Caf1 chaperone subunit complex) the pLDDT score is 83·2. “N” indicates N-terminal and “C” indicates C-terminal regions.

For the surface-exposed proteins anchored to the outer membrane such as Ail, Pla, and FyuA, our analysis identified peptide epitopes positioned in the external accessible parts of the protein (Fig. 3D–F). For PsaA, subunit of Psa/pH6 antigen (fimbriae), a single epitope was identified (PsaA-Ep1) (Fig. 3G). For Caf1 subunit of the F1 antigen also one epitope (Caf1-Ep1) was mapped onto the available structural model of Caf1 subunit (Fig. 3H, left) and in complex with its Caf1M chaperone (Fig. 3H, right).^28^ Taken together, our integrated approach combining epitope identification with structural mapping led to the identification of candidate epitopes for the non-anchored-membrane proteins (LcrV, YopB, YopD, Caf1, and PsaA) and for the outer membrane-anchored proteins (Ail, FyuA and Pla). Detailed description of individual epitopes, including their positions within the predicted structures and model confidence scores, is provided in the Supplementary Results.

## Discussion

In this study, we characterised the IgG responses to 18 major *Y. pestis* proteins by epitope mapping using sera from convalescent plague patients from Madagascar and from mice immunised with a live-attenuated vaccine and subsequently challenged with *Y. pestis*. Overall reactivity was higher in mice, with 187 positive peptides identified, compared to 32 in humans. The 187 murine peptides mapped to all 18 antigens analysed, whereas the 32 human peptides were restricted to eight antigens: Pla, LcrV, YopD, YopB, Ail, Caf1, PsaA, and FyuA (Table 1). Several factors may account for the observed interspecies differences: differences in immunisation routes and infectious doses, early antibiotic treatment of human patients which may limit antigen exposure, prolonged antigen exposure in mice vaccinated with a live-attenuated strain, and the use of infection-naïve seven-week-old mice as controls which likely have minimal prior microbial exposure.

The 32 peptides recognised by human convalescent sera corresponded to 27 distinct B-cell epitopes (Table 2, Fig. 3), most of which were confirmed in the mouse model. The detection of these peptide epitopes in two distinct host species suggests their potential value as immunological signatures of infection and raises the question of whether they also represent protective epitopes. Three peptide epitopes (LcrV-Ep7, YopD-Ep1, and YopD-Ep6; Table 2) were uniquely detected in humans and not recognised in mice, indicating that some epitopes may be specifically targeted by the human immune system, potentially due to species-specific antigen processing or presentation mechanisms.

We also performed a PCA analysis of the IgG responses based on the positive peptides identified within the eight proteins recognised as immunoreactive in humans. Peptides derived from YopB and YopD displayed clear discriminating patterns between patients with pneumonic and bubonic plague (Figure S4D and E) with higher peptide reactivities in pneumonic than bubonic plague (Figure S5B and C and Table S5A). In contrast, this differential response was not observed for LcrV, the third major component of the T3SS (Table S5A). One possible explanation for the differential recognition of YopB and YopD between the two clinical forms is related to tissue-specific differences in host–pathogen interactions. In the lungs, the primary target cells intoxicated by T3SS-mediated effector injection are phagocytes, including macrophages, which also function as antigen-presenting cells (APCs).^36^ Following antibiotic treatment, which all patients in this cohort received, pulmonary APCs were likely exposed to increased amounts of extracellular T3SS components released during bacterial clearance, potentially promoting enhanced antigen presentation and stronger humoral responses against proteins such as YopB and YopD. The discriminatory contribution of these peptides may therefore reflect differences in antigen exposure associated with the distinct pathophysiology of pneumonic and bubonic plague. Because YopB and YopD are essential structural components of the T3SS translocon that mediate effector delivery into host cells, their differential immunoreactivity may also indicate differences in T3SS activity or host–pathogen interactions between pulmonary and lymphatic tissues. This interpretation remains a hypothesis and will require validation in an independent cohort.

This study comprehensively maps linear peptide epitopes of *Y. pestis* recognised by immunised humans and mice. Previously, only two epitope-mapping studies have examined full-length protein sequences, each focussing on individual antigens such as Pla^37^ or Caf1 and LcrV.^38^ Among the 18 proteins selected for our analysis, eight had been previously reported as immunogenic targets, including PsaA,^39^ FyuA,^40^ Caf1,^7^ Ail,^41^ Pla,^42^ YopD,^43^ LcrV,^44^ and YopB.^45^ Importantly, the 32 peptides discriminating healthy controls from convalescent plague patients found in our study map the same eight previously recognised immunogenic proteins (Fig. 2A).

If the peptide epitopes identified in our analysis are immunodominant, the critical next question is whether any of them also function as protective epitopes. In the following sections, we discuss the protein-specific results in the context of previously published work. Among the 18 antigens included in our study, YopD, YopB, and LcrV were among the most immunogenic in both mouse and human datasets. These proteins constitute key components of the Type III secretion system (T3SS), the major virulence factor of *Y. pestis* and several other pathogenic bacteria. Antibodies targeting epitopes within these proteins could therefore contribute to neutralising *Y. pestis* pathogenicity. Previous studies have demonstrated the potential of these proteins as vaccine antigens,^4^ and recent work from some of us has shown that the presence of the T3SS (which includes YopD, LcrV, and YopB) is required for optimal protection in a mouse plague model.^46^

YopB and YopD have been shown to be protective antigens, as polyclonal antibodies targeting them conferred protection against *Y. pestis* infection in a mouse model with a F1 minus *Y. pestis* strain.^47^ Despite their demonstrated protective potential, no specific linear B-cell epitopes had previously been defined for YopD or YopB. Here, we identify five linear peptide epitopes for YopB and six for YopD (Fig. 3 and Figure S8, YopB and YopD), none of which have been reported previously.

LcrV, one of the most studied antigens for plague therapeutics and included in vaccine formulations currently in clinical trials,^4^ was the most reactive antigen in our study. However, this strong response was largely driven by a subset of high responders, consistent with the known variability of the IgG response to LcrV in humans. In agreement with this, LcrV is poorly recognised by individuals vaccinated with a *Y. pestis* live-attenuated plague vaccine.^38^ This variability may reflect attenuation-associated reductions in LcrV expression in the attenuated *Y. pestis* NIIEG vaccine strain used. Moreover, the less consistent recognition of LcrV compared with the F1 antigen in former plague patients^48^ is likely related to the fact that LcrV is 20-fold less abundant *in vivo* than F1, which is the most abundant *Y. pestis* antigen.^49^ In the present study, we identified seven linear peptide epitopes for LcrV (Fig. 3A). One epitope (LcrV-Ep6) overlaps with a previously described protective epitope (Figure S8, LcrV), whereas the remaining six have not been reported. Early studies identified a protective region spanning amino acids 168–275 of LcrV, as antibodies elicited by immunisation with this fragment conferred passive protection against plague.^50^ Subsequent work demonstrated that truncated LcrV constructs contain two protective regions, termed “minor” (residues 2–135) and “major” (residues 135–275) epitopes.^51^ The major epitope corresponds to that described by Motin et al.^50^ and led to the development of the protective monoclonal antibody mAb7·3.^51^ This epitope is suggested to be conformational, and it was later demonstrated that the amino acid N255 was essential for the activity of mAb7·3 and protection in a plague model.^52^ Among the epitopes identified in our study, four mapped to the minor epitope and two to the major epitope (Figure S8, LcrV) described by Hill et al.^51^ A protective linear epitope within residues 196–225 has also been described, based on binding of the protective monoclonal antibody BA5 to overlapping peptides spanning LcrV.^53^ This region corresponds to our peptide epitope LcrV-Ep6 and overlaps with the protective regions identified by Motin et al.^50^ and Hill et al.*,*^51^ strongly suggesting that LcrV-Ep6 represents a protective linear epitope (Figure S8, LcrV). Notably, vaccination with an LcrV Δ196–225 mutant still conferred protection,^53^ implying the existence of additional protective determinants. Another work has suggested that LcrV contains protective epitopes that are predominantly conformational, as immunisation with overlapping linear peptides (30-mer, ten-aa overlap) did not induce protection, whereas full-length LcrV did.^54^ Only one of the peptides conferred partial protection (50%), and this sequence does not correspond to any of the epitopes identified here (Figure S8, LcrV). The six identified linear epitopes therefore warrant further investigation to determine whether any could contribute to protective immunity.

Caf1, the subunit of the F1 antigen, is considered as a minor virulence factor of *Y. pestis* due to the fact that deletion mutants are still virulent in mice^55^, ^56^ but with differential effects in bubonic (attenuated) vs pneumonic plague.^57^ Caf1 is thought to facilitate immune evasion, protect the bacterium from phagocytosis,^58^ and antimicrobial peptides.^59^, ^60^ It is also the principal antigen used for plague diagnosis by ELISA and Western blot as it is unique to *Y. pestis*.^61^ Moreover, F1 is one of the most promising protective antigens for plague vaccination, as demonstrated in several animal models.^4^, ^7^ This protective potential is consistent with the high abundance and surface accessibility of F1 on the bacillus, and with the ability of F1-specific antibodies to promote opsonisation by phagocytes. Our study in humans identified only one peptide epitope (Caf1-Ep1) located close to the N-terminal region of the protein (Fig. 3H and Figure S8, Caf1) which differs from what has been reported before. Sabhnani and Rao^62^ immunised mice with Caf1 peptides predicted to be immunogenic in silico and identified a Caf1 immunogenic epitope not detected in our study (Figure S8, Caf1). In their study, immunisation was performed using synthetic peptides not covering the entire Caf1 protein sequence. Differences in epitope identification may also arise from the immunisation strategy, as our approach examined responses elicited during natural infection or after immunisation with a live-attenuated vaccine. Furthermore, a protective F1-specific mAb named F2H5 failed to bind any linear epitope in a peptide ELISA covering the full Caf1 sequence (17-mers with 12-aa overlaps), despite recognising the intact F1 antigen by Western blot and ELISA. This finding suggests that critical protective F1 epitopes may also be conformational, formed either within the folded monomer or within multimeric structures.^63^ In humans immunised with a live plague vaccine, testing of sera against an overlapping peptide library (17-mer, 11-aa offset) identified seven linear B-cell epitopes, one of which was defined as a marker of early immunisation.^38^ However, none of the epitopes identified in these previous studies have been shown to confer protection. Caf1-Ep1 localises close to the “Gd” donor-strand binding site of Caf1 (Fig. 3H, right), which has been reported as essential for F1 fibre assembly.^28^ Although this Gd strand is thought to be buried in the mature polymer, its recognition by human and mouse sera suggests transient exposure during polymer biogenesis or the presence of non-polymerised Caf1 subunits *in vivo*. Consequently, antibodies targeting this region could potentially interfere with F1 antigen assembly, and the protective potential of Caf1-Ep1 warrants further investigation.

PsaA is the major subunit of the pH6 antigen fimbriae, which assemble on the bacterial surface into a multimeric filamentous structure. The pH6 antigen is considered a key virulence factor involved in adhesion to host cells and evasion of host immune responses.^64^, ^65^ PsaA has also been shown to elicit a protective immune response.^66^ In our study, we identified a linear epitope located near the C-terminal region of the protein (Fig. 3G and Figure S8, PsaA), a region thought to be essential for correct folding and polymerisation of the fimbrial structure, which in turn is required for adhesion. This epitope may thus represent a promising target for interfering with pH6 antigen function and thereby impairing *Y. pestis* pathogenesis.

Pla is a protease that promotes tissue invasion by activating plasminogen and degrading host barriers. It also contributes to immune evasion and is essential for systemic infection, particularly in pneumonic plague.^5^ Antibodies directed against Pla have been shown to protect against pneumonic plague.^41^ Our study identified three peptide epitopes within Pla (Table 1). A previous analysis of sera from individuals vaccinated with an attenuated plague vaccine using a Pla peptide library (15-mers with ten-aa overlaps) identified a single putative immunodominant epitope, although no evidence of protective efficacy was provided.^37^ This epitope shows partial sequence homology to one of our epitopes, Pla-Ep3 (Figure S8, Pla). Pla-Ep3 is located partly within the predicted transmembrane region of Pla and is therefore partially surface-exposed and accessible to antibodies (Fig. 3F and Table 3). Our study additionally identified two epitopes (Pla-Ep1 and Pla-Ep2), but both predicted to be partially inserted on the bacterial membrane (Fig. 3F and Table 3).

**Table 3:** Priority evaluation of identified peptide epitopes based on structural and biological selection criteria.

Candidate peptide epitopes were evaluated according to the following prioritisation criteria: (i) surface exposure (score 1), (ii) presence within major virulence factors critical for bacterial pathogenicity (score 2), and (iii) occurrence in proteins previously shown to induce protective immunity against *Y. pestis* or related bacterial species with homologous sequences (score 3). Surface exposure was assessed based on two sub criteria: amino acid exposure, calculated using Solvent-Accessible Surface Area (SASA), and epitope accessibility. For each sub criterion, a score of 2 was assigned when fully met, 1 when partially met, and 0 when not met. All the scores were summed to generate a composite score, with higher-scoring peptides considered top-priority candidates for further investigation.

For proteins lacking an experimentally resolved three-dimensional structure, such as YopB or YopD, SASA-based amino acid exposure and epitope accessibility could not be directly evaluated. However, as these proteins are components of the type III secretion system (T3SS), they are expected to be surface exposed or accessible during host–pathogen interactions. Therefore, a score of 2 was assigned for the epitope accessibility sub criterion, with an intermediate SASA score of 1. The sequences of the human linear epitopes derived from Table 2 are presented, with putative core epitope residues underlined. Amino acids belonging to the core epitope are coloured in blue, whereas those predicted to be located within transmembrane regions or buried in the resolved structure are coloured in green. In the latter case, these residues are considered not surface-exposed and are assigned a surface exposure score of 0. Further details on SASA score calculations are provided in Table S7. For scores 2 and 3, numbers in superscript denote the reference on which the evaluation was based. ∗Protein structure not available.

Ail has been implicated in serum resistance as well as cell adherence and invasion of *Y. pestis*.^78^ Immunisation with Ail elicits protection against the F1-negative *Y. pestis* CO92 strain in a murine bubonic plague model, and protection against pneumonic plague following challenge with wild-type *Y. pestis* CO92.^41^ Ail therefore represents an interesting vaccine candidate. In our study, we identified two epitopes within Ail (Table 2). Notably, Ail-Ep2 maps to a region adjacent to extracellular loop 2 (EL2), which has been described as critical for cell adhesion and binding to extracellular matrix (ECM) components^78^ (Fig. 3E and Figure S8, Ail). This raises the possibility that antibodies targeting Ail-Ep2 could interfere with Ail-mediated interactions with host adhesion molecules such as laminin, fibronectin, vitronectin, heparan sulphate, and proteoglycans, as proposed by Kolodziejek et al.^78^ and interfere with host–pathogen interactions relevant to plague pathogenesis. This topic deserves further investigation.

FyuA is a major virulence determinant of *Y. pestis*, functioning as the outer-membrane receptor and transporter for yersiniabactin, a small but essential siderophore required for iron acquisition.^73^, ^74^, ^75^ To date, FyuA has not been evaluated as a protective antigen against plague. The two peptide epitopes identified in this study (FyuA-Ep1 and FyuA-Ep2) mapped to loops located within the lumen of the β-barrel structure (Fig. 3D), a region previously proposed to participate in flux control.^79^ Antibodies targeting these epitopes might interfere with the uptake of the yersiniabactin–iron complex and impair virulence. However, the deep intraluminal location of these peptides may restrict antibody accessibility, and the extent to which these regions are exposed or reachable in the native protein remains to be investigated.

Among the potential applications of our work, the primary focus is the development of mAbs, as the corresponding technologies are well established and mAbs both target the therapeutic and the diagnostic areas. The use of these epitopes for diagnostic and prognostic purposes (for instance pneumonic vs bubonic forms) represents a second potential application, however this would require substantially larger cohorts of human samples from diverse clinical cases, which are not readily available. A further long-term application is the development of peptide-based vaccines targeting the identified epitopes. However, this remains more distant, as it would require extensive validation in animal models followed by clinical trials.

For mAbs development, epitopes can be prioritised according to the following criteria: (i) localisation in surface-exposed zones and therefore likely accessibility to antibody binding, (ii) presence within major virulence factors critical for bacterial pathogenicity, and (iii) occurrence in proteins previously shown to induce protective immunity against *Y. pestis* or related bacterial species with homologous sequences. In Table 3, we quantified these three criteria to generate a composite score of the epitopes identified in Table 2 and rank them. This ranking is highly informative, with the highest-scoring epitopes located in LcrV^50^^,^^67^ and Caf1,^7^^,^^55^^,^^56^ followed by YopD,^47^^,^^69^^,^^70^ YopB^45^^,^^47^^,^^68^ and then, Ail,^41^^,^^71^^,^^72^ Pla,^41^^,^^77^ PsaA,^64^^,^^66^ and FyuA.^73^, ^74^, ^75^, ^76^ Notably, the YopB and YopD epitopes may be subject to a slight bias, as no experimental 3D structures are currently available for these proteins. Consequently, the scores of their epitopes may have been slightly underestimated. These findings confirm the strong immunodominance of the T3SS translocon proteins, consistent with previous observations.

It is important to acknowledge the potential limitations of our study. As with any observational study, several factors could theoretically act as confounders, including differences in demographic characteristics and geographical origin between cases and controls, variability in post-infection treatments, differences in sampling times following infection, and the presence of potential coinfections. As an example, to assess potential confounding effects, we performed multiple linear regression analyses incorporating sex, age group, and infection status (healthy vs convalescent) as covariates. These analyses were conducted across both the complete peptide dataset (*n* = 1079) and the subset of 32 peptides identified in the human analysis. No significant associations with sex, age group, or infection status were observed across the full dataset. In contrast, for the 32 selected peptides, IgG abundance was significantly associated only with infection status (Figure S9). Diseases co-circulating with plague in Madagascar are numerous and diverse, as is typically observed in tropical regions, regardless of the presence of plague. Consequently, it cannot be completely excluded that previous exposure to other microorganisms elicited antibodies cross-reactive with some *Y. pestis* peptides. However, several lines of evidence strongly support that the observed responses reflect a specific anti-*Y. pestis* antibody (see Supplementary Discussion). Although peptide array technology is highly reproducible owing to its stringent and standardised chemical conditions, peptide immobilisation on the arrays may represent a source of technical variability. However, the use of a common linker and three replicate spots for each peptide on each array slide reduces the potential impact of this variability (see Supplementary Discussion for details). Another limitation is the sample size. Nevertheless, the cohort size remains substantial for an epitope-mapping study and is comparable to, or larger than, those commonly reported in the field (see Supplementary Discussion). Overall, the risk of false-positive findings was mitigated through the application of stringent statistical significance thresholds and by the independent validation of the main findings in immunised mice. Additionally, this linear epitope-mapping strategy is primarily designed to identify linear B-cell epitopes and may therefore underestimate antibody responses directed against conformational epitopes, which depend on the native three-dimensional structure of the antigen. This limitation may account for the modest reactivity observed for well-known protective antigens such as Caf1 and PsaA. These potential sources of bias and study limitations are described and extensively discussed in the Supplementary Material, where we provide evidence and supporting arguments indicating that their impact is likely to be limited.

Overall, this epitope mapping study identified 27 peptide epitopes recognised by human IgGs and independently validated in mice, of which only two had been previously reported. These findings substantially expand the repertoire of known *Y. pestis* B-cell epitopes and provide a foundation for the rational design of next-generation plague vaccines, monoclonal antibody-based therapeutics, and improved diagnostic tools.

## Contributors

Conceived and designed the study: CD, MS and JFZ. Collection and evaluation of sera: MR, MS, HE-R and OA. Peptide array design: HE-R, CD and JFZ. Peptide array experiments: PH and KS. Animal work: HE-R and CD. Training for using Amadea: JPM. Training for statistical analysis: JLS. Verified and analysed the data: HE-R and AS. Provided administrative support: JP-C. All authors read and approved the final version.

## Data sharing statement

The data supporting the findings of this study are available from the Institut Pasteur de Madagascar or the Conservatoire national des arts et métiers (Cnam). The human studies were approved by the Ethics Committee of Madagascar. The Committee has strict requirements regarding the protection of biological samples and data originating from Madagascar and does not permit their public release. Accordingly, the human data underlying the findings of this study cannot be made publicly available. Access may nevertheless be granted to researchers upon reasonable request to the senior authors for the purpose of specific research projects, subject to compliance with the applicable ethical and regulatory requirements and, where necessary, approval by the relevant authorities. Mouse data are not subject to these confidentiality restrictions and can be made fully and publicly available upon request to zagury@cnam.fr.

## Declaration of interests

All the authors declare that they have no conflict of interest related to this work.

## Appendix A Supplementary data

Supplementary Tables and Figures

Western Blot
