# Supplementary material for: Identification of peptide epitopes in major antigens of Yersinia pestis from infected humans and immunised mice
Source: eBioMedicine. 2026 Oct 29;133:106523. doi: 10.1016/j.ebiom.2026.106523 (PMC13742484; doi:10.1016/j.ebiom.2026.106523)
Supplement: Supplementary file 1 [file mmc1.pdf]

Supplementary Material to: Identification of peptide epitopes in major antigens of *Yersinia pestis* from infected humans and immunised mice

Hebert Echenique-Rivera*[1,2], Arnaud Simon*[1], Jean-Louis Spadoni[1], Pavlo Holenya[7], Karsten Schnatbaum[7], Jean-Philippe Meyniel[8], Olifara Andriatefy[4], Minoarisoa Rajerison[5], Javier Pizarro-Cerdá[2,3], Christian Demeure[2]#, Matthieu Schoenhals[4,9]#, Jean-François Zagury[1,6]#

[1]Laboratoire Génomique, Bioinformatique, et Chimie Moléculaire, EA7528, Conservatoire National des Arts et Métiers (Cnam), Paris, France.
[2]Université Paris Cité, Institut Pasteur Paris, Unite de Recherche Yersinia, CNRS UMR6047, F-75015 Paris, France.
[3]Université Paris Cité, Yersinia National Reference Laboratory, WHO Collaborating Research & Reference Centre for Plague, FRA-140, Paris, France.
[4]Unité d'Immunologie des Maladies Infectieuses, Institut Pasteur de Madagascar, BP 1274, 101 Antananarivo, Madagascar.
[5]Unité Peste, Institut Pasteur de Madagascar, BP 1274, 101 Antananarivo, Madagascar.
[6]Bioinformatics Team, Peptinov, Hôpital Cochin, 27 Rue du Fbg Saint-Jacques, 75014 Paris, France.
[7]JPT Peptide Technologies GmbH, Berlin, Germany.
[8]Life Sciences Department, AdvanThink (ISoft), Parc des Algorithmes, Bâtiment Euclide, Route de l'Orme, 91190 Saint-Aubin, France.
[9]Université Paris Cité, Institut Pasteur Paris, Département d'Immunologie, Institut Pasteur, F-75015, Paris, France

*, #: These authors share an equal contribution

Content

Supplementary Results (pages 3-5)

-Selection of protein candidates for linear epitope mapping
-Mapping of the humoural response (IgG) against *Y. pestis* antigens from plasma of plague individuals
-PCA of reactive peptides distinguishes clinical manifestations of plague in humans
-Mapping of the IgG response against *Y. pestis* antigens in a mouse vaccination-challenge model
-Mapping Immunodominant epitopes onto 3D structural models

Supplementary Discussion: Limitations of the study, potential confounding factors, and their mitigation (pages 6-8)

1. Potential Confounding Factors Related to Patient Demographics and Clinical Management
2. Other potential confounding factors
3. Limitations of the study

Supplementary Tables (pages: 9-21)

Table S1. Demographic and clinical characteristics of individuals included in the IgG peptide array analysis.
Table S2. Antigens of *Y. pestis* (18) included in the peptide array.
Table S3. Amino acid sequences of the 18 *Y. pestis* proteins used in this study.
Table S4. Epitope mapping of IgG responses to *Y. pestis* antigens in human and mouse groups using FDR.
Table S5. Epitope mapping of IgG responses to *Y. pestis* antigens in bubonic and pneumonic plague subgroups.
Table S6. Analysis of the antibody response against overlapping peptides for the determination of the linear peptide epitopes.
Table S7. Calculation of Solvent-Accessible Surface Area (SASA) for peptide epitopes.

Supplementary Figures (pages: 22-38)

Figure S1. 3D structural models of *Y. pestis* proteins included in this study.
Figure S2. Experimental design for mouse immunisation and challenge study.
Figure S3. Boxplots of IgG signals for the 32 peptides identified in humans.
Figure S4. Principal component analysis (PCA) of the reactive peptides in humans.
Figure S5. Boxplots showing human IgG responses to the eight *Y. pestis* proteins for which reactive peptides were identified.
Figure S6. IgG responses of mice to *Y. pestis* peptide antigens in the peptide array.
Figure S7. Boxplots of mouse IgG responses to peptides overlapping with the 32 peptides identified in humans.
Figure S8. Sequence alignment and mapping of the 27 peptide epitopes onto their corresponding *Y. pestis* protein sequences.
Figure S9. Multiple linear regression analysis assessing potential confounding variables.
Figure S10. Western blot of *Y. pestis* proteins with sera from patients with plague collected on day 11 and Month 3 post-infection.

Supplementary Results

Selection of protein candidates for linear epitope mapping

The overall aim of this study was to identify immunodominant epitopes of *Y. pestis* antigens, to uncover promising candidates for therapeutic intervention. Eighteen proteins from *Y. pestis* strain CO92 were selected (Table S2) based on two criteria: 1) existing surface exposure to ensure accessibility to neutralising antibodies, and 2) documented role in the virulence or metabolism of *Y. pestis*. The panel comprised *Y. pestis*-specific protein and proteins shared with *Y. pseudotuberculosis*, representing several structural and functional classes. Three structural categories were considered, which could be classified as: 1) external proteins such as pseudo pilus or fimbriae components (anchored but mostly floating outside of bacteria), 2) outer membrane-anchored proteins (transporters, receptors, proteases) and 3) proteins part of a complex protruding structure (the Type III secretion apparatus). In this epitope mapping study, we targeted *Y. pestis* specific antigens, namely the F1 antigen (the main subunit Caf1 and the basement antigen Caf1M) and the Pla protease, and 15 additional antigens shared with its closely related specie *Yersinia pseudotuberculosis* (strain IP32953). These include the Type III secretion system (T3SS) related proteins (YscF, LcrV, YopD, and YopB), PsaA (subunit of the Psa/pH6 fimbriae), and tubular proteins anchored in the outer membrane (Ail, OmpA, OmpF2, OmpX, OmpT, OmpC, and YadC), and some of them directly related to metabolism (Maltoporin850, FyuA and FhuE).

To focus on the potential accessible regions to antibodies, an *in-silico* analysis was first performed to define and exclude signal peptides and protein regions predicted to be within the bacterial outer membrane (transmembrane domains), thus potentially less exposed and less accessible to antibodies. For this reason, transmembrane regions were identified from all antigens inserted in the outer membrane (OmpA, OmpF2, OmpX, OmpT, OmpC, Maltoporin, FhuE, FyuA and YadC) and removed from our analysis except those of Pla and Ail, for which all the protein sequence was analysed. When excluding transmembrane regions, we extended the surface-exposed sequence by four amino acids into the membrane to ensure adequate representation of partially embedded epitopes. For T3SS components LcrV, YscF, YopB and YopD, as well as PsaA, Caf1 and Caf1M the entire protein sequence was analysed, but putative signal peptides were removed (except for YopD and YopB). These corrections generated a final “experimental protein length” that served to prepare the list of overlapping 15-mer peptides used for the epitope mapping (Table S3).

Mapping of the humoural response (IgG) against *Y. pestis* antigens from plasma of plague individuals

To probe peptide array slides, human plasma samples were obtained from plague-convalescent individuals, from a plague-endemic region of Madagascar which were collected by Institut Pasteur de Madagascar as part of the national public health survey of Plague. Clinical plague manifestations included bubonic plague (n = 37) and pneumonic plague (n = 14). Blood sampling was performed three months following diagnosis. A group of healthy individuals with no known history of plague (26 Antananarivo blood donors) was also recruited and served as negative controls (Table S1). For both groups, plasma was tested by F1 antigen ELISA and Western Blotting for reactivity against the F1 antigen (data not shown). Healthy donors were all negative for F1 IgG by serology and plague survivors were all positive.

Peptide microarray was used to assess IgG reactivity against the individual peptides, with antibody recognition detected through fluorescence and signal intensity quantified as Mean Fluorescence intensity (MFI) for each peptide. The dataset containing the MFI signals was used to perform the bioinformatic and statistical analyses as described in Figure 1 and in the Methods section. After quality controls, IgG responses against each of the 1,079 peptides were compared between uninfected and plague samples. For this analysis, patient’s convalescent from pneumonic and bubonic plague were grouped together under “Convalescent”. For each peptide, three complementary statistical approaches were applied to identify the most significant difference between groups. Due to variability and heterogeneity within our peptide dataset, two well-known statistical tests were used, namely the non-parametric Mann-Whitney test[30] and the parametric Student’s *t*-test[31], with Bonferroni corrections.[32] In addition, an “extreme responder” analysis was also evaluated for each peptide (see Methods). Via the Mann-Whitney test, 12 peptides passed the statistical filter (Table 1) and in the Student’s *t*-test analysis, 12 peptides also passed the filter (Table 1). Between the two analyses, eight peptides were common. Twenty-five peptides met the “extreme responder” criterion (Table 1).

Analysis of the human response by False Discovery Rate

As no systematic evaluation of the immune response has been conducted to date, we applied a stringent significance threshold based on the Bonferroni correction, complemented with extreme responder approach. This conservative approach was intended to ensure the selection of the most robust peptide epitopes while minimising the risk of false-positive findings and potential confounding effects. We also compared healthy and convalescent individuals using both the Mann–Whitney test and the *t*-test, applying the less conservative false discovery rate (FDR) correction (adjusted $p < 0{\cdot}05$). As shown in Table S4A, this analysis identified a total of 43 reactive peptides. As expected, these included all 32 reactive peptides corresponding to the epitopes reported in Table 2, together with 11 additional reactive peptides, which are presented in Table S4B.

PCA of reactive peptides distinguishes clinical manifestations of plague in humans

A Principal Component Analysis (PCA) was used to assess whether some peptide sets could distinguish the different study groups. Patients with pneumonic (n = 14) and bubonic (n = 37) plague were labelled separately to evaluate how they clustered based on various peptide sets. For antigens possessing more than two reactive peptides (YopD, YopB, LcrV, FyuA, and Pla), we also performed PCAs using only these peptides (Figure S4C–G). These antigen-specific PCAs consistently separated convalescent individuals from healthy controls with the separation confirmed by the PERMANOVA test. The corresponding PERMANOVA p values for analyses using all peptides and the reactive peptide subsets, respectively, were as follows: LcrV, 0·011 and 0·002; YopB, 0·002 and 0·001; YopD, 0·001 and 0·001; FyuA, 0·098 and 0·001; and Pla, 0·125 and 0·01.

In addition to YopB and YopD shown in the main text, LcrV exhibited separation between the groups when using all LcrV peptides while focusing only on the seven most reactive peptides did not improve discrimination (Figure S4C). This suggests that a broad range of LcrV peptides, not just the most reactive ones, may contribute to the humoural response. For FyuA and Pla, PCA also clearly separated healthy and convalescent groups but did not distinguish pneumonic from bubonic infections (Figure S4F, G).

Findings on YopB and YopD prompted a direct comparison among the healthy, bubonic plague, and pneumonic plague subgroups. Differential peptide reactivity was assessed using the Mann–Whitney test and Student's t-test with Bonferroni correction ($p < 0·0002$) (Table S5B). This analysis identified a total of 15 reactive peptides: seven were specific to the healthy versus bubonic plague comparison, six were specific to the healthy versus pneumonic plague comparison, and two were shared between both comparisons. No peptide reached statistical significance in the comparison between the bubonic and pneumonic plague subgroups, likely reflecting insufficient statistical power. Table S5C lists the reactive peptides together with the corresponding p values for each comparison. Thirteen of the 15 reactive peptides overlapped with the 32 previously identified in Table 1 and listed in Table 2.

Overall, the pneumonic plague subgroup exhibited higher antibody reactivity to YopD- and YopB-derived peptides than the bubonic plague subgroup for the majority of reactive peptides (Table S5A). In contrast, extending the analysis to LcrV, the third Type III Secretion System (T3SS) protein containing reactive peptides, revealed the opposite trend, with higher antibody reactivity observed in the bubonic plague subgroup. Table S5A summarizes the mean and median antibody reactivity scores for all reactive T3SS-derived peptides across the three study groups.

Mapping of the IgG response against *Y. pestis* antigens in a mouse vaccination-challenge model

Twenty OF:1 female mice were used for the experiment. Pre-immune sera were collected seven days before vaccination (naïve group, n = 20), after which mice received an oral dose of the VTnF1 vaccine. Sera were then collected on day 21 post-vaccination (vaccinated group, n = 20). No signs of suffering were recorded after vaccination. On day 30 post-vaccination, mice were challenged subcutaneously with virulent *Y. pestis*. All animals survived, and final sera were collected 30 days post-challenge (vaccinated and challenged group, n = 20). The *Y. pestis* antigens included in the epitope mapping are highly homologous to their counterparts in the vaccine strain. IgG reactivity was analysed in a similar manner as for human samples using the three complementary statistical approaches (see Methods) (Figure 1).

For each peptide, we conducted the following paired comparisons: naïve vs vaccinated, naïve vs vaccinated and challenged, and vaccinated vs vaccinated and challenged. In the naïve vs vaccinated comparison, 292 peptides were identified as positive across all three statistical methods. These peptides significantly differentiated the two groups ($p = 6·36 \times 10^{-7}$) based on the sum of log-transformed MFI values per mouse (Figure S6A). This initial analysis revealed a broad repertoire of peptides recognised in vaccinated animals compared to naïve controls. To capture the immune response at a more mature stage, following affinity maturation and the development of higher-affinity antibodies, subsequent analyses focused on comparing naïve and vaccinated + challenged mice. This later time point, may reflect a more mature immune response, increases the likelihood of identifying peptides specifically induced by *Y. pestis* challenge. Additionally, the timing of this comparison more closely matches the sampling window used in the human dataset. The results of this analysis are described in the Main Manuscript. Additionally, the PCA performed on the full set of 1,079 peptides showed clear segregation between the two groups naïve vs vaccinated + challenged (Figure S6B). A similarly distinct separation was observed when the analysis was restricted to the 187 identified peptides (Figure S6C). These findings indicate that the identified peptides are representative of the broader immune response.

Analysis of the mouse response by False Discovery Rate
As for the human dataset, an additional analysis was performed by comparing naïve with vaccinated and challenged sera using the Mann–Whitney test and Student's *t*-test with the less stringent false discovery rate (FDR; adjusted $p < 0·05$) correction. The results are presented in Table S4A (mouse: Naïve vs Vaccinated + Challenged) and identified a total of 377 reactive peptides, substantially more than those identified using the Bonferroni correction and the extreme responder approach (377 versus 187 peptides).

Overall, the 18 proteins exhibited broader IgG peptide recognition in mice than in humans. The proteins that elicited the strongest responses in humans, namely YopB, YopD, and LcrV, also showed strong reactivity in mice (Table 1). In contrast, some of the most immunogenic proteins in mice, namely Ail, Pla, Caf1M and PsaA, elicited minimal or no detectable responses in human samples.

Mapping Immunodominant epitopes onto 3D structural models

To evaluate the accessibility of the identified peptide epitopes to antibodies, we mapped them onto predicted 3D protein structures generated using AlphaFold (alphafold.com). Among the proteins analysed, the three antigens with the highest number of mapped epitopes were LcrV (seven epitopes; Figure 3A), YopB (five epitopes; Figure 3B), and YopD (six epitopes; Figure 3C), all components of the T3SS. At the tip of the T3SS injectisome, LcrV facilitates the formation of a translocon pore in the host cell membrane by promoting assembly of the YopB/YopD complex, enabling the injection of effector Yops into the host cytoplasm.[33] The pentameric ring structure of LcrV[34] is proposed to serve as a scaffold for the assembly of YopD and YopB into multimeric ring structures.[35] These multimeric structures have not yet been resolved, and the peptide mapping shown in Figure 3 is based on monomeric AlphaFold models predicted with low confidence (pLDDT scores values of 32·06 for YopB, and 34·38 for YopD). Nevertheless, since these three proteins are localised on the bacterial surface, the identified epitopes are of interest.

For the surface-exposed proteins anchored to the outer membrane such as Ail (pLDDT = 78·56), Pla (pLDDT = 93·62), and FyuA (pLDDT = 90·44), our analysis identified peptide epitopes likely positioned in the externally accessible parts of the protein. In FyuA, two epitopes (FyuA-Ep1 and FyuA-Ep2) mapped to the lumen of the β-barrel structure (Figure 3D). In Ail, two epitopes were detected, one of which (Ail-Ep2) localised to a partially exposed loop region (Figure 3E). For Pla, three peptide epitopes mapped in regions predicted to be hydrophobic and among them one partially external to the outer membrane (Figure 3F).

For PsaA (pLDDT = 86·62), subunit of Psa/pH6 antigen (fimbriae), a single epitope was identified (PsaA-Ep1) and mapped to the monomeric subunit, as structural models for the multimeric assembly are not available (Figure 3G). The Caf1(pLDDT = 85·62) subunit of the F1 antigen forms multimeric pseudo-pilus/capsular structures that extend outward from the bacterial surface (Figure 3H, left). One epitope (Caf1-Ep1) was identified and mapped onto the available structural model of Caf1 in complex with its Caf1M chaperone.[28] Taken together, our integrated approach combining epitope identification with structural mapping led to the identification of candidate epitopes for the non-anchored-membrane proteins (LcrV, YopB, YopD, Caf1, and PsaA) and for the outer membrane-anchored proteins (Ail, FyuA and Pla).

Supplementary Discussion: Limitations of the study, potential confounding factors, and their mitigation

It is important to consider both the potential confounding factors and the inherent limitations of the present study. The principal risk for such a study is the identification of peptide epitopes that do not represent genuine *Y. pestis*-specific epitopes. Conversely, a major limitation is the possibility of failing to identify the full repertoire of peptide epitopes elicited during *Y. pestis* infection. The following sections discuss these potential sources of bias and the arguments supporting their mitigation in the context of this epitope-mapping study.

1. Potential Confounding Factors Related to Patient Demographics and Clinical Management.

Given the rarity of plague cases and the logistical challenges associated with sample collection during outbreaks, the primary objective of the collection campaigns was to obtain blood samples from infected patients whenever possible. Consequently, potential confounding factors were difficult to anticipate during the study design, as clinical samples were collected opportunistically and were both scarce and difficult to access.

Differences in clinical management. such as variations in antibiotic regimens, treatment timing, or dosage, could potentially influence the host immune response and thus represent a source of confounding. However, this possibility is unlikely to have substantially affected our study. In Madagascar, the official first-line treatment for both bubonic and pneumonic plague is streptomycin, administered immediately after diagnosis in accordance with World Health Organization (WHO) recommendations (1). Therefore, treatment protocols are highly standardised. The interval between infection and initiation of antibiotic treatment could also influence the magnitude of the immune response, particularly if patients delayed seeking medical attention or experienced prolonged travel before receiving care. Unfortunately, this information was not available from the clinical records. Nevertheless, such variability would be expected primarily to reduce the statistical power to detect antigenic peptides rather than generate false-positive epitopes and should therefore be regarded as a limitation of the study rather than a confounding factor. Importantly, these sources of variability do not apply in the very standardised mice experiments, for which no antibiotics were used, and strictly identical conditions were applied to all mice. We are thus not surprised to have replicated in mice the antigenicity of most of the peptides reactive in humans (in fact mice exhibited many more epitopes than humans).

Inspection of the demographic characteristics of cases and controls revealed some differences in age distribution and, to a lesser extent, in sex distribution. The largest age difference was observed between patients with bubonic and pneumonic plague (mean age: 17·6 vs. 42·4 years). However, this difference is unlikely to represent a major source of confounding because it reflects the distinct epidemiology of the two clinical forms of plague. Bubonic plague is typically acquired through flea bites or contact with infected animals during outdoor activities and therefore predominantly affects younger individuals, whereas pneumonic plague is primarily transmitted through person-to-person respiratory contact and more frequently affects older individuals remaining within households (2). A marked age difference was also observed between convalescent patients and uninfected controls (mean age: 24·4 vs. 48·3 years). This difference was largely driven by the younger age of patients with bubonic plague, whereas the mean age of patients with pneumonic plague (42·4 years) was much closer to that of the control group. Although immune function undergoes changes across the lifespan, immunocompetent young and older adults generally mount comparable adaptive immune responses to infectious agents. Consequently, it appears unlikely that the differential peptide reactivity observed between cases and controls can be explained solely by age differences. Because both cases and controls encompassed a broad age range, the potential confounding effects of age and sex on peptide reactivity were evaluated using multiple linear regression. Log_{10}-transformed mean fluorescence intensity (MFI) values were used as the dependent variable, with exposure status (*Y. pestis*-infected versus uninfected), age, and sex included as explanatory variables (see Methods section for details). Analyses were performed both on the complete dataset of 1,079 peptides and on the subset of the 32 peptides identified as significantly reactive in humans. Exposure to *Y. pestis* was strongly associated with peptide signal intensity when the analysis was restricted to the 32 positive peptides ($p = 6{\cdot}12 \times 10^{-9}$), whereas no association was detected when all peptides were analysed together ($p = 0{\cdot}497$). In contrast, neither age nor sex showed a significant association with peptide reactivity in either analysis. These results indicate that the differential responses observed for the positive peptides are primarily attributable to prior *Y. pestis* exposure rather than to demographic differences between cases and controls (Supplementary Figure S9).

Peptide immobilisation represents a potential technical confounder, as differences in peptide accessibility following immobilisation could influence antibody binding and, consequently, the measured MFI. In this study, however, all peptides were immobilised using the same Ttds (tandem/thiol-containing disulfide linker) linker, which is commonly used to conjugate peptides to other moieties and has been successfully employed in numerous peptide-based serological screening studies. The use of a common linker and a standardised immobilisation procedure reduces variability attributable to differences in conjugation chemistry across peptides. Furthermore, peptides were immobilised through a reactive moiety located at the end of the linker, leaving their side chains available for antibody binding and thereby promoting peptide accessibility. Nevertheless, peptide-specific differences in accessibility following immobilisation cannot be completely excluded. Finally, each peptide was represented by three replicate spots on each slide, and the corresponding MFI values were combined, thereby reducing the influence of spot-to-spot technical variability.

2. Other potential confounding factors.

Importantly, the control samples originated from the same geographic region as the convalescent samples but were collected in different years (Table S1). Diseases co-circulating with plague in Madagascar are numerous and diverse, as is typically observed in tropical regions, regardless of the presence of plague. Consequently, it cannot be completely excluded that previous exposure to other microorganisms elicited antibodies cross-reactive with some *Y. pestis* peptides. However, several lines of evidence strongly support that the observed responses reflect a specific anti-*Y. pestis* antibody response:

1. patients with plague tested positive for the F1 antigen, which is specific to *Y. pestis,* and this strongly indicates that the infected group was indeed infected with this pathogen.
2. for several patients, paired serum samples collected at 11 days and 3 months post-infection were available. Comparative Western blot analyses performed on these samples using *Y. pestis* antigens revealed highly similar antibody reactivity profiles, although with reduced intensity at 3 months (see Figure S10). This indicates that the anti-*Y. pestis* antibody response detected at 3 months is consistent with that observed at 11 days post-infection.
3. both study groups (confirmed infected individuals and controls without prior infection) originated from the same geographic region (the central highlands of Madagascar). Therefore, it is reasonable to assume that cases and controls were exposed to broadly similar environmental microorganisms aside from *Y. pestis*. To further increase confidence in peptide specificity, a stringent Bonferroni correction was applied to define positive signals.
4. the results obtained in humans are confirmed by results obtained from immunised mice which were exposed exclusively to the plague vaccine as the sole antigen source. These mice were seven weeks old at the time of immunisation, and their naïve sera (day 0) showed only low background reactivity in the peptide array.
5. the last argument for the specificity of our results is an a posteriori validation by comparison with the literature. The 8 proteins identified with positive peptide responses in our study have been reported to be immunogenic in the literature from independent studies.

3. Limitations of the study

Epitope-mapping studies generally involve relatively small patient cohorts because of their cost and complexity. Furthermore, access to patients with plague is inherently restricted. Consequently, the sample size in the present study was determined by the availability of clinically well-characterised serum samples. Our cohort comprised 51 patients (37 with bubonic plague, 14 with pneumonic plague) and 26 controls, this represents a relatively large cohort and is comparable to some of the largest human epitope-mapping studies reported to date, including those by Tornesello *et al*. (3) (control $n = 30$, test $n = 79$), Henze *et al*. (4) (control $n = 21$, test $n = 105$), and Oliveira *et al*. (5) (control $n = 18$, test $n = 71$). Many other epitope-mapping studies have been conducted with much smaller cohorts, such as Bihani *et al*. (6) (control $n = 10$, test $n = 19$), Holenya *et al*. (7) (control $n = 12$, test $n = 24$), and Hotop *et al*. (8) (control $n = 12$, test $n = 24$).

The combination of stringent statistical analyses, including Bonferroni correction, and independent validation in the mouse immunisation model enabled the robust identification of a set of reproducible putative B-cell epitopes (Table 2 and Table S6). indicating that the study was sufficiently powered to detect the strongest antibody targets. Nevertheless, additional *Y. pestis* epitopes may have remained undetected because of the limited sample size and the conservative statistical thresholds applied. As discussed above, the eight proteins identified in the present study are also the only *Y. pestis* proteins that have previously been reported to contain B-cell epitopes in independent studies. Although this concordance does not constitute formal statistical validation, it provides strong independent support for the biological relevance and robustness of our findings (the probability of identifying exactly these 8 proteins out of the 18 examined purely by chance is exceedingly low: $1/2^{18} = 3 \cdot 8 \times 10^{-6}$).

The functional characterisation of the identified epitopes was beyond the scope of the present study. Among the 27 human epitopes identified, only two have previously been described in the literature, and one overlaps a region known to contain a neutralising epitope. We do not infer that the remaining 25 identified epitopes are neutralising. However, it is plausible that a subset, particularly those located within major virulence factors, may contribute to protective antibody responses. To facilitate future functional investigations, we provide complementary information that may help prioritize candidate epitopes for neutralisation studies. Notably, the previously reported neutralising epitope (9) ranked among the highest-scoring epitopes identified in our analysis (Table 3).

Finally, no statistically significant differences were detected between patients with bubonic and pneumonic plague after correction for multiple testing. This finding may indicate that the two clinical forms elicit broadly similar humoural immune responses. Alternatively, it may reflect the limited statistical power associated with the relatively small number of pneumonic plague cases included in the cohort. Additional studies involving larger independent cohorts will therefore be important to determine whether subtle differences in epitope recognition exist between the two clinical presentations.

References

1. World Health Organization. Operational guidelines on plague surveillance, diagnosis, prevention and control. 14 June 2010.
2. Boisier P, Rahalison L, Rasolomaharo M, Ratsitorahina M, Mahafaly M, Razafimahefa M, et al. Epidemiologic features of four successive annual outbreaks of bubonic plague in Mahajanga, Madagascar. Emerg Infect Dis. 2002 Mar;8(3):311-6.
3. Tornesello AL, Botti C, Micillo A, Labonia F, Isgrò MA, Meola S, Russo L, Cavalcanti E, Sale S, Nicastro C, Atripaldi L, Starita N, Cerasuolo A, Reimer U, Holenya P, Buonaguro L, Buonaguro FM, Tornesello ML. Immune profiling of SARS-CoV-2 epitopes in asymptomatic and symptomatic pediatric and adult patients. J Transl Med. 2023;21(1):123. doi:10.1186/s12967-023-03963-5.
4. Henze L, Braun J, Meyer-Arndt L, Jürchott K, Schlotz M, Michel J, Grossegesse M, Mangold M, Dingeldey M, Kruse B, Holenya P, Mages N, Reimer U, Eckey M, Schnatbaum K, Wenschuh H, Timmermann B, Klein F, Nitsche A, Giesecke-Thiel C, Loyal L, Thiel A. Primary ChAdOx1 vaccination does not reactivate pre-existing, cross-reactive immunity. Front Immunol. 2023;14:1056525.
5. Oliveira JR, Ruiz CMR, Machado RRG, Magawa JY, Daher IP, Urbanski AH, Schmitz GJH, Arcuri HA, Ferreira MA, Sasahara GL, de Medeiros GX, Júnior RCVS, Durigon EL, Boscardin SB, Rosa DS, Schechtman D, Nakaya HI, Cunha-Neto E, Gadermaier G, Kalil J, Coelho V, Santos KS. Immunodominant antibody responses directed to SARS-CoV-2 hotspot mutation sites and risk of immune escape. Front Immunol. 2023;13:1010105.
6. Bihani S, Ray A, Borishetty D, Tuckley C, Salkar A, Acharjee A, Shrivastav P, Shrivastav O, Shastri J, Agrawal S, Duttagupta S, Srivastava S. Investigation of immunoreactivity profiles and epitope landscape in divergent COVID-19 trajectories and SARS-CoV-2 variants. J Proteome Res. 2025;24(2):762-776.
7. Holenya P, Lange PJ, Reimer U, Woltersdorf W, Panterodt T, Glas M, Wasner M, Eckey M, Drosch M, Hollidt JM, Naumann M, Kern F, Wenschuh H, Lange R, Schnatbaum K, Bier FF. Peptide microarray-based analysis of antibody responses to SARS-CoV-2 identifies unique epitopes with potential for diagnostic test development. Eur J Immunol. 2021;51(7):1839-1849.
8. Hotop SK, Reimering S, Shekhar A, Asgari E, Beutling U, Dahlke C, Aschenbrenner D, Br√∂nstrup M. Peptide microarrays coupled to machine learning reveal individual epitopes from human antibody responses with neutralizing capabilities against SARS-CoV-2. Emerg Microbes Infect. 2022;11(1):1037-1048.
9. Quenee LE, Berube BJ, Segal J, Elli D, Ciletti NA, Anderson D, et al. Amino acid residues 196–225 of LcrV represent a plague protective epitope. Vaccine. 2010 Feb 17;28(7):1870-1876.

Supplementary Table S1. Demographic and clinical characteristics of individuals included in the IgG peptide array analysis.

(A) Demographic characteristics of study participants in terms of age and gender. Values are presented as *n* (%) for categorical variables and mean age (years) for continuous variables. Healthy controls (*n* = 26), convalescent individuals (*n* = 51). The convalescent group (*n* = 51) is divided into two groups of patients with bubonic (*n* = 37) or pneumonic (*n* = 14) plague. The younger age observed in patients with bubonic plague may reflect greater outdoor exposure to infected fleas among younger individuals (Boisier *et al.,* 2002).

(B) Demographic and clinical characteristics of individual donors included in the study. Each row corresponds to a single participant. NA: not applicable.

Table S1A

Group	Total	Males *n*,%	Females *n*,%	Age Mean (years)	Male Age Mean (years)	Female Age Mean (years)
Healthy	26	8 (30·8%)	18 (69·2%)	48·3	45·8	49·4
Convalescent	51	25 (45%)	26 (51%)	24·4	21·8	26·85
Bubonic	37	17 (45·9%)	20 (54·1%)	17·6	15·8	19·1
Pneumonic	14	8 (57·1%)	6 (42·9%)	42·4	34·6	52·7

Table S1B

	Type of patient	Type of plague	Year of blood collection	Sex	Age (years)	Origin of sample	Time of sample collection post exposure to *Y. pestis*	Specie
1	Healthy Donor	NA	2019	female	43	Central highlands Madagascar	NA	human plasma
2	Healthy Donor	NA	2019	male	19	Central highlands Madagascar	NA	human plasma
3	Healthy Donor	NA	2020	male	65	Central highlands Madagascar	NA	human plasma
4	Healthy Donor	NA	2020	female	82	Central highlands Madagascar	NA	human plasma
5	Healthy Donor	NA	2019	female	47	Central highlands Madagascar	NA	human plasma
6	Healthy Donor	NA	2019	female	66	Central highlands Madagascar	NA	human plasma
7	Healthy Donor	NA	2020	female	64	Central highlands Madagascar	NA	human plasma
8	Healthy Donor	NA	2019	female	60	Central highlands Madagascar	NA	human plasma
9	Healthy Donor	NA	2019	female	46	Central highlands Madagascar	NA	human plasma
10	Healthy Donor	NA	2020	female	64	Central highlands Madagascar	NA	human plasma
11	Healthy Donor	NA	2019	female	60	Central highlands Madagascar	NA	human plasma
12	Healthy Donor	NA	2019	female	46	Central highlands Madagascar	NA	human plasma
13	Healthy Donor	NA	2019	male	37	Central highlands Madagascar	NA	human plasma
14	Healthy Donor	NA	2020	female	58	Central highlands Madagascar	NA	human plasma
15	Healthy Donor	NA	2019	male	43	Central highlands Madagascar	NA	human plasma
16	Healthy Donor	NA	2020	male	31	Central highlands Madagascar	NA	human plasma
17	Healthy Donor	NA	2019	female	69	Central highlands Madagascar	NA	human plasma
18	Healthy Donor	NA	2020	female	65	Central highlands Madagascar	NA	human plasma
19	Healthy Donor	NA	2019	male	51	Central highlands Madagascar	NA	human plasma
20	Healthy Donor	NA	2020	female	13	Central highlands Madagascar	NA	human plasma
21	Healthy Donor	NA	2019	male	61	Central highlands Madagascar	NA	human plasma
22	Healthy Donor	NA	2020	female	10	Central highlands Madagascar	NA	human plasma
23	Healthy Donor	NA	2019	female	7	Central highlands Madagascar	NA	human plasma
24	Healthy Donor	NA	2020	female	4	Central highlands Madagascar	NA	human plasma
25	Healthy Donor	NA	2020	male	59	Central highlands Madagascar	NA	human plasma
26	Healthy Donor	NA	2019	female	85	Central highlands Madagascar	NA	human plasma
27	Convalescent	Bubonic	2021	female	15	Central highlands Madagascar	month 3	human plasma
28	Convalescent	Bubonic	2022	male	9	Central highlands Madagascar	month 3	human plasma
29	Convalescent	Bubonic	2021	female	15	Central highlands Madagascar	month 3	human plasma
30	Convalescent	Bubonic	2021	female	18	Central highlands Madagascar	month 3	human plasma
31	Convalescent	Bubonic	2021	male	10	Central highlands Madagascar	month 3	human plasma
32	Convalescent	Bubonic	2021	male	10	Central highlands Madagascar	month 3	human plasma
33	Convalescent	Bubonic	2020	male	14	Central highlands Madagascar	month 3	human plasma
34	Convalescent	Bubonic	2021	female	11	Central highlands Madagascar	month 3	human plasma
35	Convalescent	Bubonic	2021	male	8	Central highlands Madagascar	month 3	human plasma
36	Convalescent	Bubonic	2021	male	7	Central highlands Madagascar	month 3	human plasma
37	Convalescent	Bubonic	2020	female	11	Central highlands Madagascar	month 3	human plasma
38	Convalescent	Bubonic	2021	male	16	Central highlands Madagascar	month 3	human plasma
39	Convalescent	Bubonic	2021	male	12	Central highlands Madagascar	month 3	human plasma
40	Convalescent	Bubonic	2021	female	40	Central highlands Madagascar	month 3	human plasma
41	Convalescent	Bubonic	2020	female	7	Central highlands Madagascar	month 3	human plasma
42	Convalescent	Bubonic	2021	female	12	Central highlands Madagascar	month 3	human plasma
43	Convalescent	Bubonic	2022	female	8	Central highlands Madagascar	month 3	human plasma
44	Convalescent	Bubonic	2021	male	11	Central highlands Madagascar	month 3	human plasma
45	Convalescent	Bubonic	2021	female	58	Central highlands Madagascar	month 3	human plasma
46	Convalescent	Bubonic	2021	male	2	Central highlands Madagascar	month 3	human plasma
47	Convalescent	Bubonic	2021	male	30	Central highlands Madagascar	month 3	human plasma
48	Convalescent	Bubonic	2022	female	9	Central highlands Madagascar	month 3	human plasma
49	Convalescent	Bubonic	2021	female	11	Central highlands Madagascar	month 3	human plasma
50	Convalescent	Bubonic	2021	female	20	Central highlands Madagascar	month 3	human plasma
51	Convalescent	Bubonic	2021	female	9	Central highlands Madagascar	month 3	human plasma
52	Convalescent	Bubonic	2021	female	8	Central highlands Madagascar	month 3	human plasma
53	Convalescent	Bubonic	2021	male	12	Central highlands Madagascar	month 3	human plasma
54	Convalescent	Bubonic	2021	female	13	Central highlands Madagascar	month 3	human plasma
55	Convalescent	Bubonic	2022	female	36	Central highlands Madagascar	month 3	human plasma
56	Convalescent	Bubonic	2021	male	23	Central highlands Madagascar	month 3	human plasma
57	Convalescent	Bubonic	2021	male	18	Central highlands Madagascar	month 3	human plasma
58	Convalescent	Bubonic	2022	male	44	Central highlands Madagascar	month 3	human plasma
59	Convalescent	Bubonic	2022	male	16	Central highlands Madagascar	month 3	human plasma
60	Convalescent	Bubonic	2022	female	19	Central highlands Madagascar	month 3	human plasma
61	Convalescent	Bubonic	2022	male	27	Central highlands Madagascar	month 3	human plasma
62	Convalescent	Bubonic	2022	female	30	Central highlands Madagascar	month 3	human plasma
63	Convalescent	Bubonic	2022	female	32	Central highlands Madagascar	month 3	human plasma
64	Convalescent	Pneumonic	2021	female	56	Central highlands Madagascar	month 3	human plasma
65	Convalescent	Pneumonic	2021	female	30	Central highlands Madagascar	month 3	human plasma
66	Convalescent	Pneumonic	2021	male	12	Central highlands Madagascar	month 3	human plasma
67	Convalescent	Pneumonic	2021	female	65	Central highlands Madagascar	month 3	human plasma
68	Convalescent	Pneumonic	2021	female	21	Central highlands Madagascar	month 3	human plasma
69	Convalescent	Pneumonic	2021	male	74	Central highlands Madagascar	month 3	human plasma
70	Convalescent	Pneumonic	2021	female	74	Central highlands Madagascar	month 3	human plasma
71	Convalescent	Pneumonic	2021	male	29	Central highlands Madagascar	month 3	human plasma
72	Convalescent	Pneumonic	2021	male	23	Central highlands Madagascar	month 3	human plasma
73	Convalescent	Pneumonic	2021	female	70	Central highlands Madagascar	month 3	human plasma
74	Convalescent	Pneumonic	2021	male	7	Central highlands Madagascar	month 3	human plasma
75	Convalescent	Pneumonic	2021	male	54	Central highlands Madagascar	month 3	human plasma
76	Convalescent	Pneumonic	2021	male	32	Central highlands Madagascar	month 3	human plasma
77	Convalescent	Pneumonic	2021	male	46	Central highlands Madagascar	month 3	human plasma

Supplementary Table S2: Antigens of *Y. pestis* (18) included in the peptide array.
Presentation of the selected proteins including several features such as localisation or function, and relevant information such as Uniprot[1] number (RRID:SCR_002380) at https://www.uniprot.org/ or Gene number in the *Y. pestis* CO92 annotation[2] (GenBank at https://www.ncbi.nlm.nih.gov/genbank/). Abbreviations: T3SS: Type III Secretion System. The pLDDT (predicted Local Distance Difference Test) scores from Alphafold2 are shown with values ranging from 0 - 100. Higher pLDDT values reflect greater local structural confidence: >90 = very high, 70–90 = good, 50–70 = low, <50 = very low and often disordered.

Protein	Uniprot Number [1]	Prediction confidence (pLDDT)	Family / structure	Macromolecular structure / oligomers (when known)	Function	Cellular Localisation	YPO locus [2]	DNA form	Reference (see below)
**YscF**	O68691	72·19	T3SS	Injectisome (needle) / polymer	Injectisome brick, immunomodulation	External	RS00320 / YPCD1.55	pYV / pCD1 plasmid	1
**YopB**	O68698	32·06	T3SS	Translocon /(putative) pentamer	Pore for Yops injection	External	RS00190 / YPCD1.29.c	pYV / pCD1 plasmid	2
**YopD**	O68699	34·38	T3SS	Translocon /(putative) pentamer	Pore for Yops injection	External	RS00185 / YPCD1.28.c	pYV / pCD1 plasmid	2
**LcrV**	P0C7U7	85·38	T3SS	Injectisome tip / pentamer	Injectisome-translocon connection	External	RS00200 / YPCD1.31.c	pYV / pCD1 plasmid	3
**Caf1**	P26948	85·62	Pseudo capsule	subunit of F1 pseudocapsule / multimeric filaments	Protection against antimicrobial peptides & phagocytosis	External	RS00975 / YPMT1.84	pFra / pMT1 plasmid	4
**Caf1M**	P26926	86·25	Pseudo capsule	F1 pseudocapsule basement	Pseudocapsule anchor	Periplasm	RS00965 / YPMT1.82	pFra / pMT1 plasmid	4
**PsaA**	P31522	86·62	Fimbriae	subunit of Psa / pH6 fimbriae (multimeric filaments)	Adhesion, resistance to phagocytosis	External	RS07495 / YPO1303	Chromosome	5
**YadC**	A0A7Y8RFP 1	76·38	Autotranspo rter adhesin (YadA-like)	Filaments / trimer	Adhesion	Outer membrane	RS07930 / YPO1388	pYV / pCD1 plasmid	6
**Ail**	A0A5P8YI02	78·56	Ail family	8-strand β-barrel / monomer	Adhesion, complement inhibition, anti-inflammatory	Outer membrane	RS15495 / YPO2905	Chromosome	7
**OmpX**	A0A5P8YHJ5	67·19	Ail family	8-strand β-barrel / monomer	Adhesion, complement inhibition	Outer membrane	RS13525 / YPO2506	Chromosome	8
**OmpA**	Q8ZG77	78·56	Ail family	8-strand β -barrel + intracellular tail / monomer	Intracellular survival	Outer membrane	RS08165 / YPO1435	Chromosome	9
**Pla**	P17811	93·62	Omptin	10-strand β-barrel	Protease, plasminogen activator, adhesin	Outer membrane	RS00035 / YPPCP1.07	pPla / pPCP1 plasmid	10
**OmpT (Pla2 / YcoA)**	A0A2U2H416	90·38	Omptin	10-strand β-barrel	Non-peptidase Pla homolog	Outer membrane	RS07155 / YPO1231	Chromosome	11
**OmpC**	A0A5P8YDT 4	92·81	Porin	16-strand β -barrel + tail / Homotrimer	Nutriments capture, bile resistance	Outer membrane	RS07115 / YPO1222	Chromosome	12
**OmpF2 (OmpC2)**	A0A2U2H458	93·75	Porin	16-strand β -barrel + plug / Homotrimer	unknown	Outer membrane	RS07030 / YPO1205	Chromosome	13
**Maltoporin**	A4TIF8	93·56	Sugar Transporter	18-strand β -barrel	Maltose transport	Outer membrane	RS05180 / YPO0850	Chromosome	14
**FyuA (Psn)**	P46359	90·44	Siderophore receptor	22-strand β -barrel + plug	Iron, Zinc & pigments capture / yersiniabactin & pesticin receptor	Outer membrane	RS10515 / YPO1906	Chromosome	15
**FhuE (TonB dependent)**	Q0WGN5	88·44	Siderophore receptor	22-strand β - barrel + plug	TonB-dependent iron-siderophore receptor	Outer membrane	RS08665 / YPO1537	Chromosome	16

References

1 Osei-Owusu P, Jessen Condry DL, Toosky M, Roughead W, Bradley DS, Nilles ML. The N terminus of type III secretion needle protein YscF from *Yersinia pestis* functions to modulate innate immune responses. *Infect Immun*. 2015;83(4):1507-22.

2 Håkansson S, Bergman T, Vanooteghem J-C, Cornelis G, Wolf-Watz H. YopB and YopD constitute a novel class of *Yersinia* Yop proteins. *Infect Immun*. 1993;61(1):71-80.

3 Mueller CA, Broz P, Müller SA, Ringler P, Erne-Brand F, Sorg I, et al. The V-antigen of *Yersinia* forms a distinct structure at the tip of injectisome needles. *Science*. 2005;310(5748):674-6.

4 Knight SD. Structure and assembly of *Yersinia pestis* F1 antigen. In: Carniel E, Hinnebusch BJ, editors. *Yersinia: Molecular and cellular biology*. Vol. 603. Springer; 2007. p. 74-87.

5 Bao R, Nair MKM, Tang W, Esser L, Sadhukhan A, Holland RL, et al. Structural basis for the specific recognition of dual receptors by the homopolymeric pH 6 antigen (Psa) fimbriae of *Yersinia pestis*. *Proc Natl Acad Sci U S A*. 2013;110(3):1065-70.

6 Forman S, Wulff CR, Myers-Morales T, Cowan C, Perry RD, Straley SC. *yadBC* of *Yersinia pestis*, a new virulence determinant for bubonic plague. *Infect Immun*. 2008;76(2):578-87.

7 Kolodziejek AM, Hovde CJ, Minnich SA. *Yersinia pestis* Ail: Multiple roles of a single protein. *Front Cell Infect Microbiol*. 2012;2:103.

8 Kolodziejek AM, Sinclair DJ, Seo KS, Schnider DR, Deobald CF, Rohde HN, et al. Phenotypic characterization of OmpX, an Ail homologue of *Yersinia pestis* KIM. *Microbiology*. 2007;153(9):2941-51.

9 Chen Y, Duan R, Li X, Li K, Liang J, Liu C, et al. Homology analysis and cross-immunogenicity of OmpA from pathogenic *Yersinia enterocolitica*, *Yersinia pseudotuberculosis*, and *Yersinia pestis*. *Mol Immunol*. 2015;68:290-9.

10 Sebbane F, Uversky VN. *Yersinia pestis* plasminogen activator. *Biomolecules*. 2020;10(11):1554.

11 Järvinen HM, Laakkonen L, Haiko J, Johansson T, Juuti K, Suomalainen M, et al. Human single-chain urokinase is activated by the omptins PgtE of *Salmonella enterica* and Pla of *Yersinia pestis* despite mutations of active site residues. *Mol Microbiol*. 2014;89(3):507-17.

12 Stenkova AM, Bystritskaya EP, Guzev KV, Rakin AV, Isaeva MP. Molecular evolution of the *Yersinia* major outer membrane protein C (OmpC). *Evol Bioinform Online*. 2016;12:185-91.

13 Stenkova AM, Isaeva MP, Shubin FN, Rasskazov VA, Rakin AV. Trends of the major porin gene (*ompF*) evolution: Insight from the genus *Yersinia*. *PLoS One*. 2011;6(5):e20546.

14 Brzostek K, Heleszko H, Hrebenda J. Maltoporins and maltose-binding proteins of *Yersinia enterocolitica*. *J Gen Microbiol*. 1993;139(2):195-201.

15 Fetherston JD, Lillard JW Jr, Perry RD. Analysis of the pesticin receptor from *Yersinia pestis*: Role in iron-deficient growth and possible regulation by its siderophore. *J Bacteriol*. 1995;177(7):1824-33.

16 Sauer M, Hantke K, Braun V. Ferric-coprogen receptor FhuE of *Escherichia coli*: Processing and sequence common to all TonB-dependent outer membrane receptor proteins. *J Bacteriol*. 1987;169(5):2044-9.

Supplementary Table S3: Amino acid sequences of the 18 *Yersinia pestis* proteins used in this study.
The blue sequences indicate protein regions included in the preparation of the peptide array. Regions shown in black and barred correspond to segments excluded since they were identified as signal peptides or transmembrane domains, leading to the experimental protein sequence and length (1) used for the peptide array preparation. The final number of 15-mer peptides used for the coverage of the target protein is also provided (2). Abbreviations: aa = amino acids.

Protein	Experimental protein lengths (aa)[1]	Final peptide number[2]	Protein Sequences
YscF	78	17	~~MSNFSGFTK~~GTDIADLDAVAQTLKKPADDANKAVNDSIAALKDKPD<br>NPALLADLQHSINKWSVIYNINSTIVRSMKDLMQGILQKFP
YopB	355	86	~~MSALITHDRSTPVTGSLLPYVETPAPAPLQTQQVAGELKDKNGGV~~<br>~~S~~SQGVQLPAPLAVVASQVTEGQQQEVTKLLESVTRGAAGSQLISNYV<br>SVLTKFTLASPDTFEIELGKLVSNLEEVRKDIKIADIQRLHEQNMKKIE<br>ENQEKIKETEENAKQVKKSGIASKIFGWLSAIASVIVGAIMVASGVGA<br>VAGAMMVASGVIGMANMAVKQAAEDGLISQEAMKILGPILTAIEVA<br>LTVVSTVMTFGGSALKCLANIGAKLGANTASLVAKGAEFSAKVAQIS<br>TGISNTVGSAVTKLGGSFAGLTMSHAIRTGSQATQVAVGVGSGITQTI<br>NNKKQADLQHNNADLALNKADMAALQSIIDRLKEELSHLSESHQQV<br>MELIFQMINAKGDMLHNLAGRPHTV
YopD	306	74	MTINIKTDSPIITTGSQLDAITTETVKQSGEIKKTEDTRHEAQAIKSSEAS<br>LSRSQVPELIKPSQGINVALLSKSQGDLNGTLSILLLLLELARKAREMG<br>LQQRDIENKATITAQKEQVAEMVSGAKLMIAMAVVSGIMAATSTVAS<br>AFSIAKEVKIVKQEQILNSNIAGREQLIDTKMQQMSNIGDKAVSREDIG<br>RIWKPEQVADQNKLALLDKEFRMTDSKANAFNAATQPLGQMANSAI<br>QVHQGYSQAEVKEKEVNASIAANEKQKAEEAMNYNDNFMKDVLRLI<br>EQYVSSHTHAMKAAFGVV
LcrV	326	79	MIRAYEQNPQHFIEDLEKVRVEQLTGHGSSVLEELVQLVKDKNIDISIK<br>YDPRKDSEVFANRVITDDIELLKKILAYFLPEDAILKGGHYDNQLQNGI<br>KRVKEFLESSPNTQWELRAFMAVMHFSLTADRIDDDILKVIVDSMNH<br>HGDARSKLREELAELTAELKIYSVIQAEINKHLSSSGTINIHDKSINLMD<br>KNLYGYTDEEIFKASAEYKILEKMPQTTIQVDGSEKKIVSIKDFLGSEN<br>KRTGALGNLKNSYSYNKDNNELSHFATTCSDKSRPLNDLVSQKTTQL<br>SDITSRFNSAIEALNRFIQKYDSVMQRLLDDTSGK
Caf1	149	35	~~MKKISSVIAIALFGTIATANA~~ADLTASTTATATLVEPARITLTYKEGA<br>PITIMDNGNIDTELLVGTLTLGGYKTGTTSTSVNFTDAAGDPMYLTFT<br>SQDGNNHQFTTKVIGKDSRDFDISPKVNGENLVGDDVVLATGSQDFF<br>VRSIGSKGGKLAAGKYTDAVTVTVSNQ
Caf1M	233	56	~~MILNRLSTLGIITFGMLSFAANSAQ~~PDIKFASKEYGVTIGESRIIYPLD<br>AAGVMVSVKNTQDYPVLIQSRIYDENKEKESEDPFVVTPPLFRLDAK<br>QQNSLRIAQAGGVFPRDKESLKWLCVKGIPPKDEDIWVDDATNKQKF<br>NPDKDVGVFVQFAINNCIKLLVRPNELKGTPIQFAENLSWKVDGGKLI<br>AENPSPFYMNIGELTFGGKSIPSHYIPPKSTWAFDLPKGLAGARNVSW<br>RIINDQGGLDRLYSKNVTL
PsaA	114	26	~~MKMKCFAKNALAVTTLMIAACGMANASTVINSKDVSGEVTVKQ~~<br>~~G~~NTFHVDFAPNTGEIFAGKQPGDVTMFTLTMGDTAPHGGWRLIPTGD<br>SKGGYMISADGDYVGLYSYMMSWVGIDNNWYINDDSPKDIKDHLYV<br>KAGTVLKPTTYKFTGRVEEYVF
YadC	495	121	~~MQRMQCIYIATWLIAVFTSPI~~VIANNTNINGSTNNNGNGTINIFDASS<br>NNDIHTLTGLGNEQLGGFSNHLIDSHNNTIDGGQSNNLVSSDGNMISA<br>ISLGDGLFYGAQNNTLINSNNNLLIVTQGSTIIDSDSNTVSGISNNLIESN<br>SNIIGNENSCYSDPASPSGAWCVDNQNTLIGSDNNTITGALNGLHNSH<br>NNDIIASSVNNLMDTHNNIIAGGHYNTISGGGNNDIFGSENNVTDSTD<br>ANINGSNNYVIDGNGIGRDDLTEDNSILGGSGNMGVGDSVTAITNSVV<br>FGGNTSGNSTGSTLTDSVSVSGNGTSGNNVVNIGGAANGNNSASLGT<br>GSVSSEGGIALGSGSIATRNDELNIGDRQITSVKKGVENTDTINVSQLN<br>DSFDDVLNLSNEYSDNSFSTVTENINNYTDASLDTVLNTTGEYTDNSI<br>LLVTNESNNYTDNGMESVSNYANIYADESLLAIYNEEANYMSNLIDV<br>TLNNANNYTDLSVNTIIYTGKQYTDSRINEYQRTFKNEFLT~~YSNGKFG~~<br>~~GFDKDINQKQKQLNAGIAATMAAAVIPQKSGSKVSIGVGLAGYSDQG~~<br>~~AGSVGAIWHVNQRITMNTTMTYDTQRGVSLLTGLSIGI~~

**Ail**	**155**	**36**	~~**MNKTLLVSSLIACLSIASVNVYAE**~~GESSISIGYAQSRVKEDGYKLDKNPRGFNLKYRYEFNNDWGVIGSFAQTRRGFEESVDGFKLIDGDFKYYSVTAGPVFRINEYVSLYGLLGAGHGKAKFSSIFGQSESRSKTSLAYGAGLQFNPHPNFVIDASYEYSKLDDVKVGTWMLGAGYRF
**OmpX**	**127**	**29**	~~**MKKIACLSAVAACVLAVTAGSAFAGQ**~~STVSGGYAQSDYQGVANKSSGFNLKYRYEWSDSQLGYITSFTHTEKSGFGDEAVYNKAQYNAIT~~GGPAYRINDWASIYG~~LVGVGHGRFTQNESAFVGDKHSTSDYGFTYGAGLQFNPAENVALDVSYEQSRIRNVDVGTWVA~~GVGYTF~~
**OmpA**	**100**	**23**	~~**MKKTAIALAVALVGFATVAQA**APKDNTWYTGG~~KLGWSQYQDTGSIINNDGPTHKDQLGA~~GAFFGYQANQYLGFEMG~~YDWLGRMPYKGDINNGAFKAQGVQ~~LAAKLSYPVAQDLDVYTRLGGL~~VWRADAKGSFDGGLDRASGHDTGVS~~PLVALGAEYAWTKNWATRM~~EYQWVNNIGDRETVGARPDNGLLSV~~GVSYRFGQEDAAAPIVAPTPAPAPIVDTKRFTLKSDVLFGFNKANLKPEGQQALDQLYAQLSSIDPKDGSVVVLGFADRIGQPAPNLALSQRRADSVRDYLVSKGIPADKITARGEGQANPVTGNTCDNVKPRAALIECLAPDRRVEIEVKGYKEVVTQPQA~~
**Pla**	**291**	**70**	~~**MKKSSIVATIITILSGSANAA**~~SSQLIPNISPDSFTVAASTGMLSGKSHEMLYDAETGRKISQLDWKIKNVAILKGDISWDPYSFLTLNARGWTSLASGSGNMDDYDWMNENQSEWTDHSSHPATNVNHANEYDLNVKGWLLQDENYKAGITAGYQETRFSWTATGGSYSYNNGAYTGNFPKGVRVIGYNQRFSMPYIGLAGQYRINDFELNALFKFSDWVRAHDNDEHYMRDLTFREKTSGSRYYGTVINAGYYVTPNAKVFAEFTYSKYDEGKGGTQTIDKNSGDSVSIGGDAAGISNKNYTVTAGLQYRF
**OmpT (Pla2 / YcoA)**	**214**	**51**	~~**MNKEALAKKGAGALALLILSHPAVANNNT**DTAGNVTVS~~TSLGLLNAESIESVYQSSEPNQKLSQLNWEANNTPIVKM~~DIAWDALPRLTLTA~~KGWSTLSSGQGVMDNYDWLTPQQTQWSDWSHHEKTNLNYANEIDIN~~AKIWFLKQHNYRVGP~~ILGYQENNQSWTAYGGNFNYNNGANIFTAPDGIVGVGYKQKFDMLYIGL~~AGSYRYQQLEF~~NALLKYSNWVNANAYDNHYNRKVSFKDSSKNSSYYSAVI~~DVGYYFTENTKLF~~VEAAWNQYSEGRGETQTLNYATGNVGSSKSGIEYQSQTLTV~~GFQYKF~~
**OmpC**	**185**	**44**	~~**MKLRVLSFIIPALLVAGSASA**AEIYNKDGNKLDLYGKIDGLHYFSDNKNLDGDQSYMRFGLKGETQITDQLTGYGQWE~~YQVNLNKAENEDGNHDSFTRVGFA~~GLKFADYGSLDYGRNYGVLYDVTSWTDVLPEFGGDTYGADNFLSQRGNGMLTYRNTNFFGLVDGLNF~~ALQYQGKNGSSSETNNGRGVADQNGDGYGMSL~~SYDLGWGVSA~~SAAMASSLRTTAQNDLQYGQGKRANAYTGG~~LKYDANNVYLAA~~NYTQTYNLTRFGDFSNRSSDAAFGFADKAHNIEVVAQY~~QFDFGLR~~PSVAYLQSKGKDIGIYGDQDLLKYVDIGA~~TYFFNKNMS~~TYVDYKINLLDKNDFTKNARINTDDIVAVGMV~~YQF~~
**OmpF2 (OmpC2)**	**227**	**55**	~~**MKRNILAILIPTLLVATTSHAA**EIYNKDGNKLDLY~~GKVKALHYFSDNTKSDGDKSYVRLGF~~KGVTQITDELSGYGQW~~EYNFAANYAESQEAKDNKTRL~~AFAGLRYGNLGSIDYGRNYG~~VLYDIAAWTDMLPEFGNDSYTRTD~~NFMTGRTTGVATYRNTDFFGLVDGLKFS~~LQYQGKNGAEGETNNGRTDTSKQNGDGFGLSS~~SYEIGAGVSV~~GAAYASSNRTLAQKNSTFGKGDKADAWTTG~~LKYSDNGVY~~LAANYAETRNMTPISGTAVINNVSTSVSGFANKTQNIELV~~AQYLFDFGLK~~PSIAYIQSKGKDIEGIGDTDLVKYVDIG~~ATYYFNKNMST~~YVDYKINQLNDDNKLKLNTDNVVALG~~LVYQF~~

**Maltoporin**	**182**	**43**	~~**MKTSLRTLSVALAAALVSPSVLAIEK**IDFHGYMRAGVGVSSDGGLAEWQKTMVGRLGNESDTYGEIGLGAEVYKKEDVSFYLD~~SMVSMLSDGSNDSETTIGDDAQFGLRQ~~LNLQIKGLIPGDKEAVIWGGKRYYQRHDLHIIDTKYWNISGSGAGIENYTVGPGAVSV~~AWVRGDANDVDTRITGDSDVNINYID~~VRYAGFKPWAGSWTEVG~~IDYAMPNPTKQQKEYGGLYDADNAVM~~LTGEISQDMFGGYNKLVLQYANKGLAQN~~MISQGGGWYDMWHKTDEAKGYRVIN~~TGLIPITDKFSFNHV~~LTWGSANDITEYTDKTNLISLV~~GRAQYQFTQYVR~~AIGEVGGFYQKDTYHNGSNYKQGGEKYT~~IALGLAEGPDFLSRP~~ELRVFASYLNDSENGKPFEDGTSNDTWNF~~GVQVEAWW~~
**FyuA**<br>**(Psn)**	**673**	**118**	~~**MKMTRLYPLALGGLLLPAIANAQTSQQDESTLVV**~~TASKQSSRSASANNVSSTVVSAPELSDAGVTASDKLPRVLPGLNIENSGNMLFSTISLRGVSSAQDFYNPAVTLYVDGVPQLSTNTIQALTDVQSVELLRGPQGTLYGKSAQGGIINIVTQQPD~~STPRGYIEGGVSSRDSYRSKFNLSGPIQDGLLYGSVTL~~LRQVDDGDMINPATGSDDLGGTRASIGNV~~KLRLAPDDQPWEMG~~FAASRECTRATQDAYVGWNDIKGRKLSISDGSPDPYMRRCTDSQTLS~~GKYTTDDWVF~~NLISAWQQQHYSRTFPSGSLIVNMPQRWNQDVQE~~LRAATLGDARTVDM~~VFGLYRQNTREKLNSAYDMPTMPYLSSTGYTTAETLAA~~YSDLTWHLTDRFDIG~~GGVRFSHDKSSTQYHGSMLGNPFGDQGKSNDDQVLGQ~~LSAGYMLTDDWRVY~~TRVAQGYKPSGYNIVPTAGLDAKPFVAEKSINYEL~~GTRYETADVTLQA~~ATFYTHTKDMQLYSGPVRMQTLSNAGKADATGVELEAKWRFAPGWSWDINGNVIRSEFTNDSELYHGNRVPFVPRYGAGS~~SVNGVIDTRYGALM~~PRLAVNLVGPHYFDGDNQLRQGTYATLDS~~SLGWQATERMNIS~~VYVDNLFDRRYRTYGYMNGSSAVAQVNMGRTVGIN~~TRIDFF~~
**FhuE (TonB dependent)**	**473**	**116**	~~**MRILEKNHGKSRVRFNCTHPIKPAAWVLAANVSLLGCAYAATDENNSQKKERENNPANTTITVTASPLRHAGVTEDSGSYNTSSMST**ATGLNISARETPQSVSVLTKQRMRDQNLN~~SVESAVNNITGISVRQFDSDRFGFTSRGMAVNNVMRDGVATFYDTRFNYGDNTLDTDMFDRIEVVRGAAG~~LMAGPGNPSAVINLVRKRPTQDFRGSVSAGVGSWEKWRTALDISGPLNSEGSVRGRFVT~~AYEDKNSFVNRYDQSKNPFYG~~ILEIDVTPNTLFT~~FGADTQRTLTRGGMFGGLPLFNSAGGRTNYAQSATTASDWASAETRTQTLFSSLQHNFDNGWNIKGTFTFDNDKLRQDVMWPTGYPDPQTNIGMRPGSLSLIDGARRQQNYDIQVNGQYSLFGRQHQLGLGWNRQRQNIDNDYYLATCNATRTCPDLGDFTQPGWQYPKPVWSDKRAYGSKGRSDQSSQYVVT~~QLSLIDPLTLIL~~GGRLTTWETRGDNFGTPQNARYKNEFVPYS~~GLTYDINRDLSVYT~~SYTEIFNPENRRDRNNTLLAPVSGQNYEAGLK~~GVAFDNSLDYSL~~AVFEIRQNNMPVPDTTAPRLPDNSQPYFAVDGTKTRGF~~EAEVSGKMTEDWNISAG~~YTQYNVKLPSSNTQTPVTPVTPRKVLKLFT~~TYTLPGQLSDLTL~~GGGVNWQSQIARNLSSPIGIQQIGQGSFAIYSL~~MSRYQFTPQLSLTVNLDNL~~FNQHYYTQIGQYNQYLLGAPRNVDAT~~VRYTF~~

Supplementary Table S4: Epitope mapping of IgG responses to *Y. pestis* antigens in human and mouse groups using FDR.

(A) The number of peptides identified for each study group (human or mouse) is shown. For humans, the compared groups correspond to healthy individuals (n = 26) versus convalescent plague individuals (3 months post-infection; n = 51). For mice (n = 20), the naïve group (7 days before immunisation) was compared with the vaccinated (day 21 post immunisation) and challenged group (day 30 post challenge). The number of peptides identified using two statistical tests with FDR ranking is shown for humans and mice. The combined results of the two tests are shown for humans and for mice. For both datasets, "Reactivity (%)" corresponds to the percentage of positive peptides identified in both analyses with respect to the total number of peptides derived from each protein target tested in the peptide array. The total number of peptides identified in each analysis is shown at the bottom of the table.

(B) List of the additional peptides identified in humans using the FDR analysis in the comparison healthy versus convalescent individuals. Pep = peptide.

Table S4A

Antigens				Human (Healthy vs Convalescent)				Mouse (Naïve vs Vaccinated + Challenged)			
**Protein name**	**Length (aa)**	**Experimental size of protein (aa)**	**Peptide number in Array (15 aa each)***	**Mann Whitney (FDR)**	**Student's *t*-test (FDR)**	**Combination of peptides**	**Reactivity (%)**	**Wilcoxon (FDR)**	**Student's *t*-test (FDR)**	**Combination of peptides**	**Reactivity(%)**
**YscF**	87	78	17	0	0	0	0	6	2	6	35·3 (6/17)
**YopB**	401	355	86	7	4	10	11·6 (10/86)	31	22	31	36·1 (31/86)
**YopD**	306	306	74	10	8	13	17·6 (13/74)	27	14	27	36·5 (27/74)
**LcrV**	326	326	79	3	5	5	6·3 (5/79)	46	35	47	59·5 (47/79)
**Caf1**	170	149	35	3	4	6	17·1 (6/35)	13	11	13	37·1 (13/35)
**Caf1M**	258	233	56	0	0	0	0	38	32	38	67·9 (38/56)
**PsaA**	158	114	26	1	2	2	7·7 (2/26)	15	12	15	57·7 (15/26)
**YadC**	615	495	121	0	0	0	0	18	17	19	15·7 (19/121)
**Ail**	179	155	36	0	1	1	2·8 (1/36)	23	19	23	63·9 (23/36)
**OmpX**	174	127	29	0	0	0	0	11	10	11	37·9 (11/29)
**OmpA**	353	100	23	0	0	0	0	3	1	3	13·0 (3/23)
**Pla**	312	291	70	1	3	3	4·3 (3/70)	45	32	45	64·3 (45/70)
**OmpT**	311	214	51	0	0	0	0	17	13	17	33·3 (17/51)
**OmpC**	371	185	44	0	0	0	0	9	7	9	20·5 (9/44)
**OmpF2**	371	227	55	0	0	0	0	9	8	9	16·7 (9/54)
**Maltoporin850**	419	182	43	0	0	0	0	10	8	10	23·3 (10/43)
**FyuA**	673	481	118	1	3	3	2·5 (3/118)	27	19	27	22·9 (27/118)
**FhuE**	753	473	116	0	0	0	0	26	22	27	23·3 (27/116)
**Total**			1,079	26	30	43		374	284	377	

Table S4B

Additional peptides identified by the FDR analysis "Healthy vs Convalescent"		
**Antigen**	**Peptide**	**Sequence**
Caf1	Caf1_Pep_010	IDTELLVGTLTLGGY
	Caf1_Pep_024	SRDFDISPKVNGENL
	Caf1_Pep_034	LAAGKYTDAVTVTVS
FyuA	FyuA_Pep_019	NPAVTLYVDGVPQLS
LcrV	LcrV_Pep_006	VEQLTGHGSSVLEEL
YopB	YopB_Pep_012	YVSVLTKFTLASPDT
	YopB_Pep_016	EIELGKLVSNLEEVR
	YopB_Pep_043	GLISQEAMKILGPIL
	YopB_Pep_045	KILGPILTAIEVALT
YopD	YopD_Pep_006	ITTETVKQSGEIKKT
	YopD_Pep_064	VKEKEVNASIAANEK

Table S5. Epitope mapping of IgG responses to *Y. pestis* antigens in bubonic and pneumonic plague subgroups.

(A) Mean and median values of reactivity of the peptides of Table 2 derived from proteins of T3SS, namely YopB, YopD, and LcrV in the 3 subgroups healthy subjects, bubonic and pneumonic plague patients. In bold, the peptides identified as significant in the comparison of either bubonic or pneumonic subgroup with healthy controls (peptides from Table S5C). In red colour the peptide that appear significant in the subgroup analysis (healthy versus pneumonic) not present in Table 2. One can note that the largest difference is observed for the responses of the pneumonic subgroup to some specific peptides of YopB and YopD. T3SS = Type III Secretion System.

(B) This table presents the comparison of the IgG responses to *Y. pestis* peptides of either patients with bubonic plague (n = 37) or patients with pneumonic plague (n = 14) with Healthy controls (n = 26). The number of positive peptides identified by statistical analyses (Mann-Whitney or Student's t-test with Bonferroni correction, $p < 0{\cdot}0002$) for individual antigens is given in the blue columns for bubonic plague and in the red columns for pneumonic plaque. This table presents the comparison of the IgG responses to *Y. pestis* peptides of either patients with bubonic plague (n = 37) or patients with pneumonic plague (n = 14) with Healthy controls (n = 26). The number of peptides identified using statistical analyses (Mann-Whitney or Student's t-test with Bonferroni correction, $p < 0{\cdot}0002$) for individual antigens (in blue for bubonic plague, in red for pneumonic plaque). The combined number of peptides identified by the two statistical analyses is also shown. The reactivity (%) corresponds to the percentage of positive peptides identified with respect to the total number of peptides derived from each target protein tested in the peptide array. The total number of positive peptides identified in each analysis is provided at the bottom of the table.

(C) List of peptides identified as reactive (i.e. positive after Bonferroni correction) in comparisons between healthy controls and plague subgroups. The 13 peptides shown in black are among the 32 peptides previously identified in the comparison between healthy controls and convalescent patients, whereas the two peptides shown in red represent newly identified reactive peptides. P values highlighted in bold correspond to those that passed the Bonferroni correction threshold ($p < 0{\cdot}0002$) in each comparison (for either of the two statistical tests). aa = amino acid.

Table S5A

Antigen	Peptide	Sequence	Healthy		Bubonic		Pneumonic	
			Mean (MFI)	Median (MFI)	Mean (MFI)	Median (MFI)	Mean (MFI)	Median (MFI)
YopB	**YopB_Pep_001**	SQGVQLPAPLAVVAS	611,06	1,00	4726,34	162,33	**12812,26**	**2983,83**
	**YopB_Pep_020**	**DIKIADIQRLHEQNM**	154,12	94,17	1644,23	311,00	**12959,24**	**1776,17**
	YopB_Pep_030	ASKIFGWLSAIASVI	1858,19	1864,17	3016,65	1904,17	2686,21	1372,00
	**YopB_Pep_048**	**ALTVVSTVMTFGGSA**	1444,04	1183,33	2717,26	1864,17	**5462,21**	**4115,50**
	**YopB_Pep_071**	**TQTINNKKQADLQHN**	219,08	122,00	2389,33	384,67	**15020,24**	**948,50**
	**YopB_Pep_072**	**NNKKQADLQHNNADL**	245,71	193,00	1353,86	443,67	**13825,98**	**918,17**
	YopB_Pep_078	LQSIIDRLKEELSHL	1616,41	1601,00	2463,63	1965,33	1996,71	1535,83
YopD	YopD_Pep_011	EAQAIKSSEASLSRS	5834,15	4984,00	14379,09	11312,33	22694,24	10084,33
	YopD_Pep_019	SKSQGDLNGTLSILL	4993,00	3978,50	13885,95	8864,33	10907,00	8313,00
	**YopD_Pep_037**	**VASAFSIAKEVKIVK**	5109,82	5246,83	**10668,96**	**7425,67**	7760,12	7646,00
	YopD_Pep_041	EQILNSNIAGREQLI	852,83	708,50	4767,95	1215,33	6663,52	921,17
	**YopD_Pep_049**	**EDIGRIWKPEQVADQ**	1177,77	427,00	**3933,81**	**1955,67**	**24646,60**	**7549,33**
	**YopD_Pep_050**	**RIWKPEQVADQNKLA**	1073,17	167,33	**5494,08**	**2121,67**	**30107,31**	**23079,83**
	**YopD_Pep_061**	**AIQVHQGYSQAEVKE**	942,96	340,83	**2786,44**	**1229,00**	16629,10	1045,50
LcrV	LcrV_Pep_012	DISIKYDPRKDSEVF	4965,31	4672,83	10589,59	6820,17	4703,65	2555,83
	**LcrV_Pep_015**	**EVFANRVITDDIELL**	16222,81	8989,00	**36129,55**	**42666,17**	33733,36	37582,50
	LcrV_Pep_019	KILAYFLPEDAILKG	2939,55	2868,00	5645,52	4345,67	4949,33	2787,33
	LcrV_Pep_027	LESSPNTQWELRAFM	1411,12	1426,00	2235,40	1905,33	3225,50	1672,00
	LcrV_Pep_042	KIYSVIQAEINKHLS	4568,96	4664,17	7139,24	5827,00	6251,71	5202,67
	**LcrV_Pep_052**	**EIFKASAEYKILEKM**	8203,42	7479,17	**15182,38**	**13856,50**	13842,71	11125,00
	LcrV_Pep_076	AIEALNRFIQKYDSV	2236,36	1982,33	4777,51	3718,67	6395,43	2000,83

Table S5B

Antigens				Healthy vs Bubonic				Healthy vs Pneumonic			
				Bonferroni ($p < 0{\cdot}0002$)		Results		Bonferroni ($p < 0{\cdot}0002$)		Results	
**Protein name**	**Length (aa)**	**Experimental size of protein (aa)**	**Peptide number in Array (15 aa each)***	**Mann Whitney**	**Student's *t*-test**	**Combination of peptides**	**Reactivity (%)**	**Mann Whitney**	**Student's *t*-test**	**Combination of peptides**	**Reactivity (%)**
**YscF**	87	78	17	0	0	0	0	0	0	0	0
**YopB**	401	355	86	0	0	0	0	5	0	5	5·8 (5/86)
**YopD**	306	306	74	3	1	4	5·4 (4/74)	2	0	2	2·7 (2/74)
**LcrV**	326	326	79	2	2	2	2·5 (2/79)	0	0	0	0
**Caf1**	170	149	35	0	0	0	0	1	0	1	2·9(1/35)
**Caf1M**	258	233	56	0	0	0	0	0	0	0	0
**PsaA**	158	114	26	1	0	1	3·8 (1/26)	0	0	0	0
**YadC**	615	495	121	0	0	0	0	0	0	0	0
**Ail**	179	155	36	0	0	0	0	0	0	0	0
**OmpX**	174	127	29	0	0	0	0	0	0	0	0
**OmpA**	353	100	23	0	0	0	0	0	0	0	0
**Pla**	312	291	70	0	1	1	1·4 (1/76)	0	0	0	0
**OmpT**	311	214	51	0	0	0	0	0	0	0	0
**OmpC**	371	185	44	0	0	0	0	0	0	0	0
**OmpF2**	371	227	55	0	0	0	0	0	0	0	0
**Maltoporin850**	419	182	43	0	0	0	0	0	0	0	0
**FyuA**	673	481	118	1	1	1	0·8 (1/118)	0	0	0	0
**FhuE**	753	473	116	0	0	0	0	0	0	0	0
Total			1,079	7	5	9		8	0	8	

Table S5C

Reactive peptides identified in Healthy controls versus patients with Bubonic or Pneumonic plague						
**Antigen**	**Peptide**	**Sequence**	***p* value (Healthy vs Bubonic)**		***p* value (Healthy vs Pneumonic)**	
			t-test	Mann-Whitney	*t*-test	Mann-Whitney
Caf1	**Caf1_Pep_034**	LAAGKYTDAVTVTVS	2,20E-02	1,90E-02	1,00E-02	**1,20E-04**
FyuA	FyuA_Pep_023	NTIQALTDVQSVELL	**4,90E-06**	**1,30E-05**	2,50E-02	1,80E-02
LcrV	LcrV_Pep_015	EVFANRVITDDIELL	**9,20E-05**	1,30E-04	2,80E-02	1,40E-02
	LcrV_Pep_052	EIFKASAEYKILEKM	**2,40E-05**	1,60E-04	6,00E-02	6,00E-03
YopB	**YopB_Pep_001**	SQGVQLPAPLAVVAS	1,60E-02	4,00E-03	5,30E-02	**4,40E-05**
	YopB_Pep_020	DIKIADIQRLHEQNM	3,00E-02	5,00E-03	3,30E-02	**1,50E-04**
	YopB_Pep_048	ALTVVSTVMTFGGSA	1,10E-02	2,00E-03	2,00E-03	**1,80E-05**
	YopB_Pep_071	TQTINNKKQADLQHN	5,20E-02	9,00E-03	3,70E-02	**6,90E-06**
	YopB_Pep_072	NNKKQADLQHNNADL	5,00E-03	5,00E-03	5,00E-02	**1,83E-06**
Pla	Pla_Pep_060	NAKVFAEFTYSKYDE	**1,20E-04**	8,30E-04	1,39E-01	2,43E-01
PsaA	PsaA_Pep_022	DIKDHLYVKAGTVLK	1,00E-03	**1,10E-04**	2,60E-02	2,10E-02
YopD	YopD_Pep_037	VASAFSIAKEVKIVK	**7,80E-05**	4,40E-04	6,00E-03	2,00E-03
	YopD_Pep_049	EDIGRIWKPEQVADQ	1,20E-02	**1,51E-07**	5,00E-03	**1,36E-07**
	YopD_Pep_050	RIWKPEQVADQNKLA	3,00E-03	**5,55E-07**	2,00E-03	**4,03E-06**
	YopD_Pep_061	AIQVHQGYSQAEVKE	1,21E-01	**9,50E-05**	2,50E-02	8,00E-03

Supplementary Table S6. Analysis of the antibody response against overlapping peptides for the determination of the linear peptide epitopes.

This table provides detailed information regarding the identification of linear peptide epitopes shown in Table 2. The 32 reactive peptides identified in humans are listed in the "Peptide Name" column (highlighted in red), along with their corresponding overlapping peptides. For each peptide, the upstream overlapping peptide is shown on the line above, and the downstream overlapping peptide is shown on the line below. In humans, peptide epitope identification was defined based on overlapping peptides showing a statistically significant difference ($p < 0{\cdot}05$, *t*-test) in the comparison between convalescent individuals ($n = 51$) and healthy individuals ($n = 26$). Significant *p* values are shown in blue. For mice, the peptide epitope is defined by the overlapping region shared by the peptides identified significantly reactive ($p < 0{\cdot}05$, *t*-test) in the comparison between vaccinated + challenged mice ($n = 20$) and naïve mice ($n = 20$). Significant *p* values are shown in green. The linear epitopes, defined by overlapping positive peptides ($p < 0{\cdot}05$), are highlighted in red in both the human and mouse peptide sequences. In the last column, the putative core epitope, corresponding to the intersection of the human and mouse linear epitopes, is underlined within the human linear epitope sequence. Pep = peptide.

Reactive Peptides in humans		Humans (Healthy vs Convalescent)		Mice (Naïve vs Vaccinated + Challenged)		Peptide Epitope	
Protein	Peptide name	Peptide sequence	*t*-test ($p < 0{\cdot}05$)	Peptide sequence	*t*-test ($p < 0{\cdot}05$)	Name	Sequence
LcrV	LcrV_Pep_011	DKNIDISIKYDPRKD	0,026	DKNIDISIKYDPRKD	0,0028		
	LcrV_Pep_012	DISIKYDPRKDSEVF	0,01	DISIKYDPRKDSEVF	0,00082	LcrV-Ep1	DISIKYDPRKD
	LcrV_Pep_013	KYDPRKDSEVFANRV	0,15	KYDPRKDSEVFANRV	0,0186		
	LcrV_Pep_014	RKDSEVFANRVITDD	0,21	RKDSEVFANRVITDD	0,013		
	LcrV_Pep_015	EVFANRVITDDIELL	0,000062	EVFANRVITDDIELL	0,0038	LcrV-Ep2	NRVITDDIELL
	LcrV_Pep_016	NRVITDDIELLKKIL	0,013	NRVITDDIELLKKIL	0,002		
	LcrV_Pep_018	ELLKKILAYFLPEDA	0,032	ELLKKILAYFLPEDA	0,014		
	LcrV_Pep_019	KILAYFLPEDAILKG	0,000087	KILAYFLPEDAILKG	0,0019	LcrV-Ep3	KILAYFLPEDA
	LcrV_Pep_020	YFLPEDAILKGGHYD	0,23	YFLPEDAILKGGHYD	0,00088		
	LcrV_Pep_026	VKEFLESSPNTQWEL	0,0072	VKEFLESSPNTQWEL	0,1		
	LcrV_Pep_027	LESSPNTQWELRAFM	0,01	LESSPNTQWELRAFM	0,15	LcrV-Ep4	LESSPNTQWEL
	LcrV_Pep_028	PNTQWELRAFMAVMH	0,47	PNTQWELRAFMAVMH	0,027		
	LcrV_Pep_041	TAELKIYSVIQAEIN	0,055	TAELKIYSVIQAEIN	0,0072		
	LcrV_Pep_042	KIYSVIQAEINKHLS	0,00034	KIYSVIQAEINKHLS	0,002	LcrV-Ep5	VIQAEINKHLS
	LcrV_Pep_043	VIQAEINKHLSSSGT	0,017	VIQAEINKHLSSSGT	0,052		
	LcrV_Pep_051	YTDEEIFKASAEYKI	0,11	YTDEEIFKASAEYKI	0,0008		
	LcrV_Pep_052	EIFKASAEYKILEKM	0,0000081	EIFKASAEYKILEKM	0,00116	LcrV-Ep6	EIFKASAEYKILEKM
	LcrV_Pep_053	ASAEYKILEKMPQTT	0,17	ASAEYKILEKMPQTT	0,026		
	LcrV_Pep_075	RFNSAIEALNRFIQK	0,36	RFNSAIEALNRFIQK	0,8		
	LcrV_Pep_076	AIEALNRFIQKYDSV	0,02	AIEALNRFIQKYDSV	0,45	LcrV-Ep7	AIEALNRFIQKYDSV
	LcrV_Pep_077	LNRFIQKYDSVMQRL	0,27	LNRFIQKYDSVMQRL	0,095		
YopB	YopB_Pep_019	EVRKDIKIADIQRLH	0,0014	EVRKDIKIADIQRLH	0,0057		
	YopB_Pep_020	DIKIADIQRLHEQNM	0,0082	DIKIADIQRLHEQNM	0,021	YopB-Ep1	DIKIADIQRLH
	YopB_Pep_021	ADIQRLHEQNMKKIE	0,83	ADIQRLHEQNMKKIE	0,0047		
	YopB_Pep_029	KSGIASKIFGWLSAI	0,042	KSGIASKIFGWLSAI	0,24		
	YopB_Pep_030	ASKIFGWLSAIASVI	0,019	ASKIFGWLSAIASVI	0,11	YopB-Ep2	ASKIFGWLSAI
	YopB_Pep_031	FGWLSAIASVIVGAI	0,88	FGWLSAIASVIVGAI	0,0064		
	YopB_Pep_047	AIEVALTVVSTVMTF	0,00094	AIEVALTVVSTVMTF	0,0054		
	YopB_Pep_048	ALTVVSTVMTFGGSA	0,00011	ALTVVSTVMTFGGSA	0,34	YopB-Ep3	VSTVMTF
	YopB_Pep_049	VSTVMTFGGSALKCL	0,03	VSTVMTFGGSALKCL	0,021		
	YopB_Pep_070	GSGITQTINNKKQAD	0,071	GSGITQTINNKKQAD	0,057		
	YopB_Pep_071	TQTINNKKQADLQHN	0,0078	TQTINNKKQADLQHN	0,016	YopB-Ep4	NNKKQADLQHN
	YopB_Pep_072	NNKKQADLQHNNADL	0,019	NNKKQADLQHNNADL	0,0023		
	YopB_Pep_073	QADLQHNNADLALNK	0,65	QADLQHNNADLALNK	0,00074		
	YopB_Pep_077	DMAALQSIIDRLKEE	0,24	DMAALQSIIDRLKEE	0,023		
	YopB_Pep_078	LQSIIDRLKEELSHL	0,0074	LQSIIDRLKEELSHL	0,11	YopB-Ep5	LQSIIDRLKEELSHL
	YopB_Pep_079	IDRLKEELSHLSESH	0,8	IDRLKEELSHLSESH	0,00023		
YopD	YopD_Pep_010	DTRHEAQAIKSSEAS	0,28	DTRHEAQAIKSSEAS	0,3		
	YopD_Pep_011	EAQAIKSSEASLSRS	0,000037	EAQAIKSSEASLSRS	0,24	YopD-Ep1	IKSSEASLSRS
	YopD_Pep_012	IKSSEASLSRSQVPE	0,024	IKSSEASLSRSQVPE	0,2		
	YopD_Pep_018	VALLSKSQGDLNGTL	0,57	VALLSKSQGDLNGTL	0,011		
	YopD_Pep_019	SKSQGDLNGTLSILL	0,000022	SKSQGDLNGTLSILL	0,061	YopD-Ep2	SKSQGDLNGTLSILL
	YopD_Pep_020	GDLNGTLSILLLLLE	0,2	GDLNGTLSILLLLLE	0,0023		
	YopD_Pep_036	ATSTVASAFSIAKEV	0,003	ATSTVASAFSIAKEV	0,0011		
	YopD_Pep_037	VASAFSIAKEVKIVK	0,0000091	VASAFSIAKEVKIVK	0,028	YopD-Ep3	FSIAKEV
	YopD_Pep_038	FSIAKEVKIVKQEQI	0,0014	FSIAKEVKIVKQEQI	0,025		
	YopD_Pep_040	IVKQEQILNSNIAGR	0,17	IVKQEQILNSNIAGR	0,0055		
	YopD_Pep_041	EQILNSNIAGREQLI	0,014	EQILNSNIAGREQLI	0,094	YopD-Ep4	NSNIAGREQLI
	YopD_Pep_042	NSNIAGREQLIDTKM	0,04	NSNIAGREQLIDTKM	0,17		
	YopD_Pep_048	AVSREDIGRIWKPEQ	0,0026	AVSREDIGRIWKPEQ	0,000000006		
	YopD_Pep_049	EDIGRIWKPEQVADQ	0,0011	EDIGRIWKPEQVADQ	0,029	YopD-Ep5	RIWKPEQVADQ
	YopD_Pep_050	RIWKPEQVADQNKLA	0,00017	RIWKPEQVADQNKLA	0,128		
	YopD_Pep_051	PEQVADQNKLALLDK	0,047	PEQVADQNKLALLDK	0,0022		
	YopD_Pep_060	MANSAIQVHQGYSQA	0,12	MANSAIQVHQGYSQA	0,27		
	YopD_Pep_061	AIQVHQGYSQAEVKE	0,0088	AIQVHQGYSQAEVKE	0,16	YopD-Ep6	HQGYSQAEVKE
	YopD_Pep_062	HQGYSQAEVKEKEVN	0,044	HQGYSQAEVKEKEVN	0,26		
Ail	Ail_Pep_007	PRGFNLKYRYEFNND	0,33	PRGFNLKYRYEFNND	0,00087		
	Ail_Pep_008	NLKYRYEFNNDWGVI	0,02	NLKYRYEFNNDWGVI	0,0039	Ail-Ep1	RYEFNNDWGVI
	Ail_Pep_009	RYEFNNDWGVIGSFA	0,048	RYEFNNDWGVIGSFA	0,0032		
	Ail_Pep_015	VDGFKLIDGDFKYYS	0,062	VDGFKLIDGDFKYYS	0,0023		
	Ail_Pep_016	KLIDGDFKYYSVTAG	0,0025	KLIDGDFKYYSVTAG	0,0013	Ail-Ep2	KLIDGDFKYYSVTAG
	Ail_Pep_017	GDFKYYSVTAGPVFR	0,08	GDFKYYSVTAGPVFR	0,00011		

PsaA	PsaA_Pep_021	DSPKDIKDHLYVKAG	0,021	DSPKDIKDHLYVKAG	0,015	PsaA-Ep1	HLYVKAG
	PsaA_Pep_022	DIKDHLYVKAGTVLK	0,00015	DIKDHLYVKAGTVLK	0,0023		
	PsaA_Pep_023	HLYVKAGTVLKPTTY	0,0013	HLYVKAGTVLKPTTY	0,000026		
	PsaA_Pep_024	KAGTVLKPTTYKFTG	0,24	KAGTVLKPTTYKFTG	0,89		
FyuA	FyuA_Pep_012	PGLNIENSGNMLFST	0,17	PGLNIENSGNMLFST	0,002	FyuA-Ep1	IENSGNMLFSTISLR
	FyuA_Pep_013	IENSGNMLFSTISLR	0,00076	IENSGNMLFSTISLR	0,05		
	FyuA_Pep_014	GNMLFSTISLRGVSS	0,061	GNMLFSTISLRGVSS	0,56		
	FyuA_Pep_022	QLSTNTIQALTDVQS	0,15	QLSTNTIQALTDVQS	0,01	FyuA-Ep2	VQSVELL
	FyuA_Pep_023	NTIQALTDVQSVELL	0,0000089	NTIQALTDVQSVELL	0,00086		
	FyuA_Pep_024	ALTDVQSVELLRGPQ	0,054	ALTDVQSVELLRGPQ	0,024		
	FyuA_Pep_025	VQSVELLRGPQGTLY	0,014	VQSVELLRGPQGTLY	0,074		
	FyuA_Pep_026	ELLRGPQGTLYGKSA	0,67	ELLRGPQGTLYGKSA	0,075		
Pla	Pla_Pep_011	QLDWKIKNVAILKGD	0,012	QLDWKIKNVAILKGD	0,002	Pla-Ep1	VAILKGD
	Pla_Pep_012	KIKNVAILKGDISWD	0,000097	KIKNVAILKGDISWD	0,057		
	Pla_Pep_013	VAILKGDISWDPYSF	0,012	VAILKGDISWDPYSF	0,003		
	Pla_Pep_046	QYRINDFELNALFKF	0,028	QYRINDFELNALFKF	0,0005	Pla-Ep2	NDFELNALFKF
	Pla_Pep_047	NDFELNALFKFSDWV	0,011	NDFELNALFKFSDWV	0,052		
	Pla_Pep_048	LNALFKFSDWVRAHD	0,46	LNALFKFSDWVRAHD	0,00084		
	Pla_Pep_059	YVTPNAKVFAEFTYS	0,18	YVTPNAKVFAEFTYS	0,067	Pla-Ep3	TYSKYDE
	Pla_Pep_060	NAKVFAEFTYSKYDE	0,000063	NAKVFAEFTYSKYDE	0,00099		
	Pla_Pep_061	FAEFTYSKYDEGKGG	0,0025	FAEFTYSKYDEGKGG	0,0011		
	Pla_Pep_062	TYSKYDEGKGGTQTI	0,025	TYSKYDEGKGGTQTI	0,68		
Caf1	Caf1_Pep_002	ASTTATATLVEPARI	0,13	ASTTATATLVEPARI	0,099	Caf1-Ep1	LVEPARITLTY
	Caf1_Pep_003	ATATLVEPARITLTY	0,0019	ATATLVEPARITLTY	0,074		
	Caf1_Pep_004	LVEPARITLTYKEGA	0,0042	LVEPARITLTYKEGA	0,019		

Supplementary Table S7. Calculation of Solvent-Accessible Surface Area (SASA) for peptide epitopes.
Candidate peptide epitopes were analysed to calculate the Solvent-Accessible Surface Area (SASA) score. The putative core epitope sequence used for SASA calculation is indicated by the underlined amino acid sequence. SASA values per amino acid were used to rank all peptides (range: 96–140), and the distribution was divided into three groups: high score (>106), medium score (96–105), and low score (<95). Because the proteins YopD and YopB lack reliable three-dimensional structures, SASA could not be directly assessed (NA). These peptides were assigned a score of 1, a non-detrimental placeholder allowing comparison with the other peptide epitopes using the additional criteria. Amino acids shown in green correspond to sequences predicted (using SWISS-MODEL) to be located within transmembrane regions or buried in the resolved structure. These residues are therefore not considered surface-exposed or accessible. For these regions, SASA was not determined (ND), and an accessibility score of 0 was assigned.
NA. not available; ND. not determined. AA = amino acid. Å² (square ångströms).

Peptide Epitope		SASA score calculation			
Name	Sequence	Score total in Å²	Number of AA	SASA total divided by the number of AA	Final SASA score
LcrV-Ep1	DISIKYDPRKD	823	7	118	2
LcrV-Ep2	NRVITDDIELL	738	7	105	1
LcrV-Ep3	KILAYFLPEDA	766	7	109	2
LcrV-Ep4	LESSPNTQWEL	775	7	111	2
LcrV-Ep5	VIQAEINKHLS	717	7	102	1
LcrV-Ep6	EIFKASAEYKILEKM	706	7	101	1
LcrV-Ep7	AIEALNRFIQKYDSV	1611	15	107	2
YopB-Ep1	DIKIADIQRLH	NA*	7	NA*	1*
YopB-Ep2	ASKIFGWLSAI	NA*	7	NA*	1*
YopB-Ep3	VSTVMTF	NA*	7	NA*	1*
YopB-Ep4	NNKKQADLQHN	NA*	7	NA*	1*
YopB-Ep5	LQSIIDRLKEELSHL	NA*	7	NA*	1*
YopD-Ep1	IKSSEASLSRS	NA*	11	NA*	1*
YopD-Ep2	SKSQGDLNGTLSILL	NA*	7	NA*	1*
YopD-Ep3	FSIAKEV	NA*	7	NA*	1*
YopD-Ep4	NSNIAGREQLI	NA*	8	NA*	1*
YopD-Ep5	RIWKPEQVADQ	NA*	11	NA*	1*
YopD-Ep6	HQGYSQAEVKE	NA*	11	NA*	1*
Ail-Ep1	RYEFNNDWGVI	ND**	7	ND**	0
Ail-Ep2	KLIDGDFKYYSVTAG	695	6	116	2
PsaA-Ep1	HLYVKAG	719	7	103	1
FyuA-Ep1	IENSGNMLFSTISLR	1087	11	99	1
FyuA-Ep2	VQSVELL	718	7	103	1
Pla-Ep1	VAILKGD	ND**	1	ND**	0
Pla-Ep2	NDFELNALFKF	ND**	6	ND**	0
Pla-Ep3	TYSKYDE	701	6	117	2
Caf1-Ep1	LVEPARITLTY	1171	11	106	2

*Protein structure not available.
**region not accessible in the predicted structure.

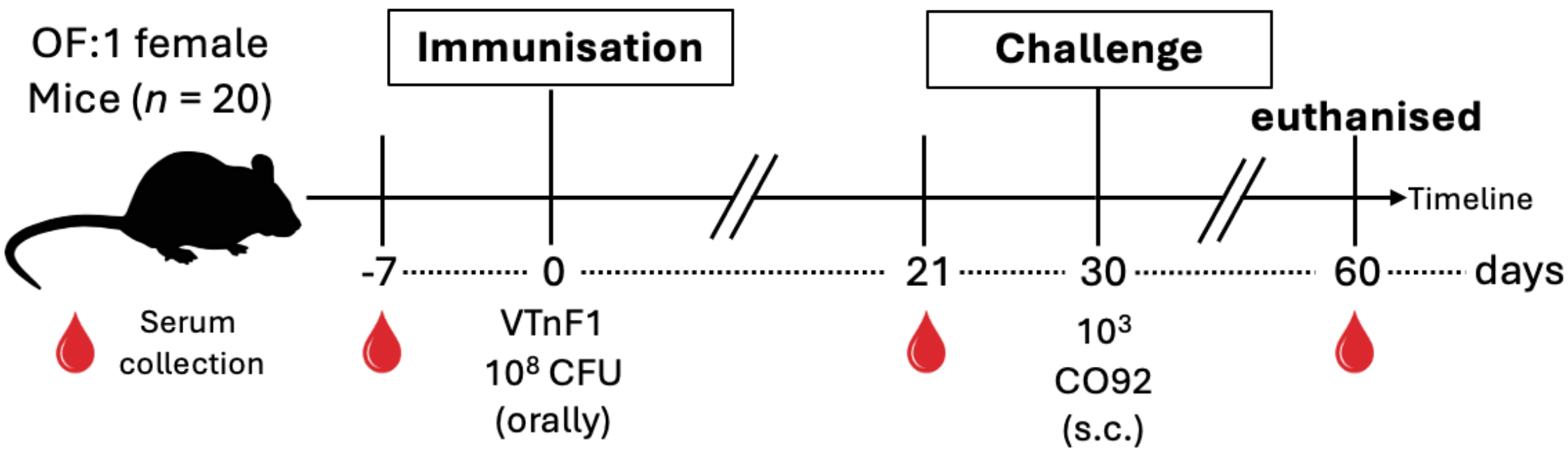

Supplementary Figure S1. Experimental design for mouse immunisation and challenge study.
Outbred female OF1 mice (n = 20) were enrolled in the study. Blood samples were collected for serum isolation on day −7 (naïve), day 21 (vaccinated), and day 60 (vaccinated and challenged). Mice were immunised orally on day 0 with 10^8 CFU of VTnF1 vaccine. On day 30 animals were challenged subcutaneously (s.c.) with 10^3 CFU of *Y. pestis* CO92 strain. Until day 60 all mice survived the challenge and mice were humanely euthanised for terminal sample collection. CFU: colony-forming units.

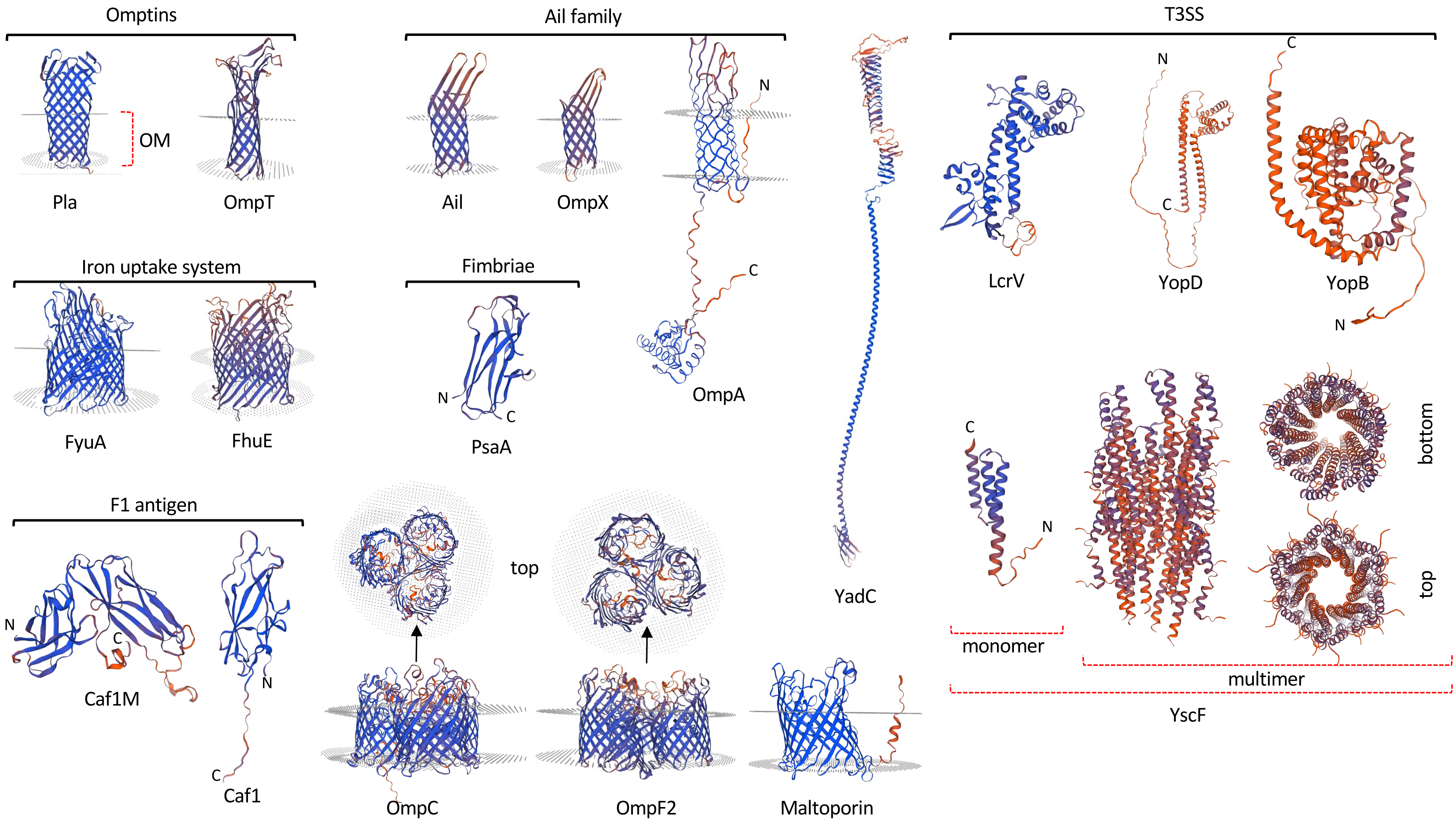
Omptins
OM
Pla
OmpT
Ail family
Ail
OmpX
N
C
OmpA
T3SS
C
N
C
LcrV
YopD
YopB
N
Iron uptake system
FyuA
FhuE
Fimbriae
N
C
PsaA
YadC
C
N
bottom
top
monomer
multimer
YscF
F1 antigen
N
C
Caf1M
N
C
Caf1
top
OmpC
OmpF2
Maltoporin

Supplementary Figure S2. 3D structural models of *Y. pestis* proteins included in this study.

Proteins are grouped based on their function: Omptins (OmpT and Pla protease); Ail family proteins (Ail, OmpX and OmpA); Type III secretion system (T3SS) components (LcrV, YopD, YscF, and YopB); subunits of F1 antigen (Caf1 and Caf1M); fimbriae subunit (PsaA); iron uptake systems (FhuE and FyuA); and additional structural/surface exposed proteins (OmpC, OmpF2, Maltoporin, and YadC). For proteins predicted to be embedded in the outer membrane (OM), transmembrane regions are shown in grey and indicate the membrane-spanning segment. marking the portion of the protein predicted to be inserted into the lipid bilayer. The protein models were built with the protein sequences of *Y. pestis* CO92 with the SWISS-MODEL online tool (https://swissmodel.expasy.org/interactive). Blue colour in the structures indicates high confidence in prediction, and orange colour indicates low confidence. For YscF, prediction of a multimeric structure was built using taking as reference the protein PrgI from *Salmonella enterica* (homo-18-mer). Views of the lumen of multimeric structure of some proteins are shown from the “top” and the “bottom”. “N” indicates the N-terminal and “C” the C-terminal region of the proteins, where these could be marked.

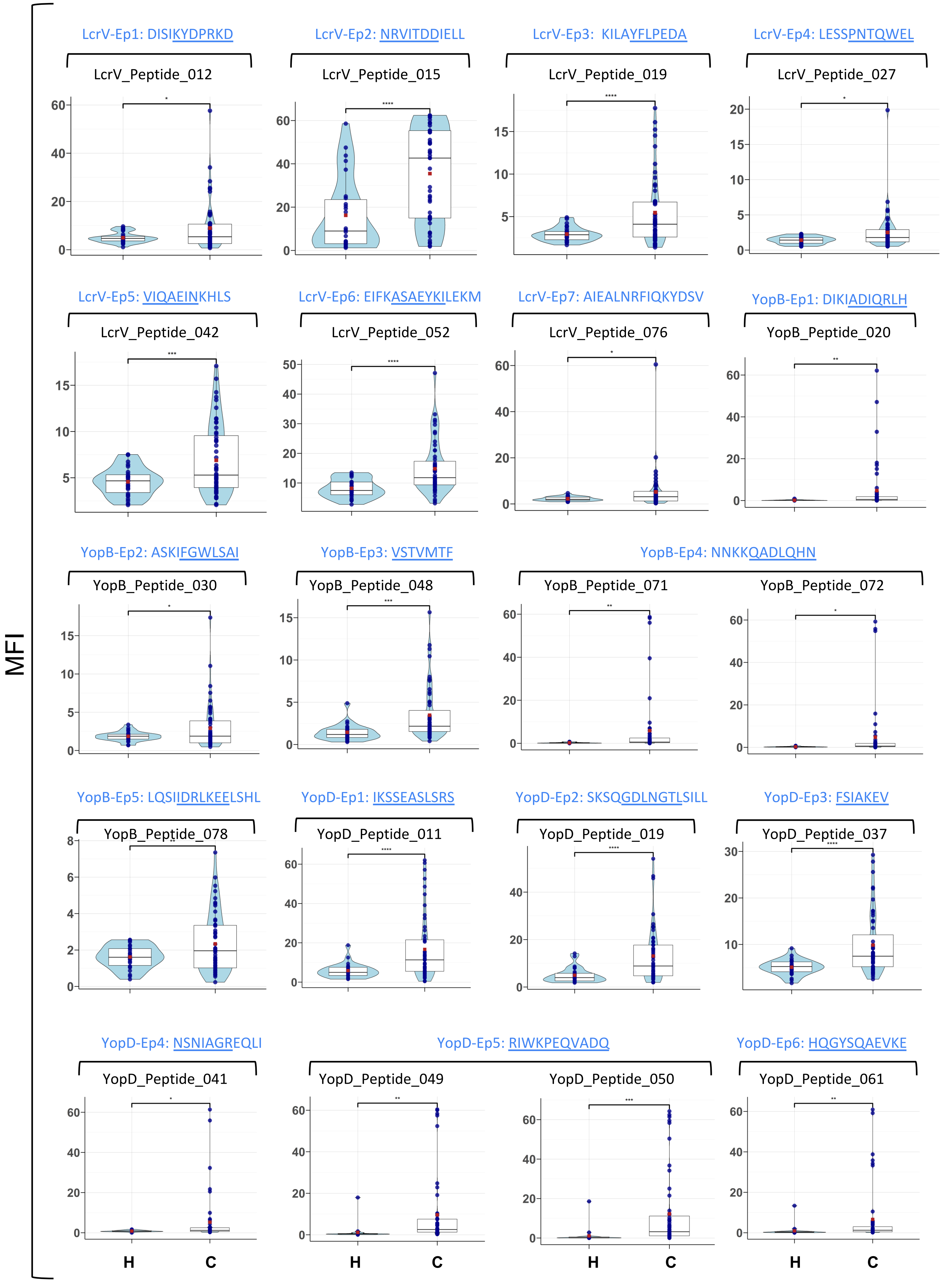
MFI
LcrV-Ep1: DISIKYDPRKD
LcrV_Peptide_012
LcrV-Ep2: NRVITDDIELL
LcrV_Peptide_015
LcrV-Ep3: KILAYFLPEDA
LcrV_Peptide_019
LcrV-Ep4: LESSPNTQWEL
LcrV_Peptide_027
LcrV-Ep5: VIQAEINKHLS
LcrV_Peptide_042
LcrV-Ep6: EIFKASAEYKILEKM
LcrV_Peptide_052
LcrV-Ep7: AIEALNRFIQKYDSV
LcrV_Peptide_076
YopB-Ep1: DIKIADIQRLH
YopB_Peptide_020
YopB-Ep2: ASKIFGWLSAI
YopB_Peptide_030
YopB-Ep3: VSTVMTF
YopB_Peptide_048
YopB-Ep4: NNKKQADLQHN
YopB_Peptide_071
YopB_Peptide_072
YopB-Ep5: LQSIIDRLKEELSHL
YopB_Peptide_078
YopD-Ep1: IKSSEASLSRS
YopD_Peptide_011
YopD-Ep2: SKSQGDLNGTLSILL
YopD_Peptide_019
YopD-Ep3: FSIAKEV
YopD_Peptide_037
YopD-Ep4: NSNIAGREQLI
YopD_Peptide_041
YopD-Ep5: RIWKPEQVADQ
YopD_Peptide_049
YopD_Peptide_050
YopD-Ep6: HQGYSQAEVKE
YopD_Peptide_061
H
C

Figure S3

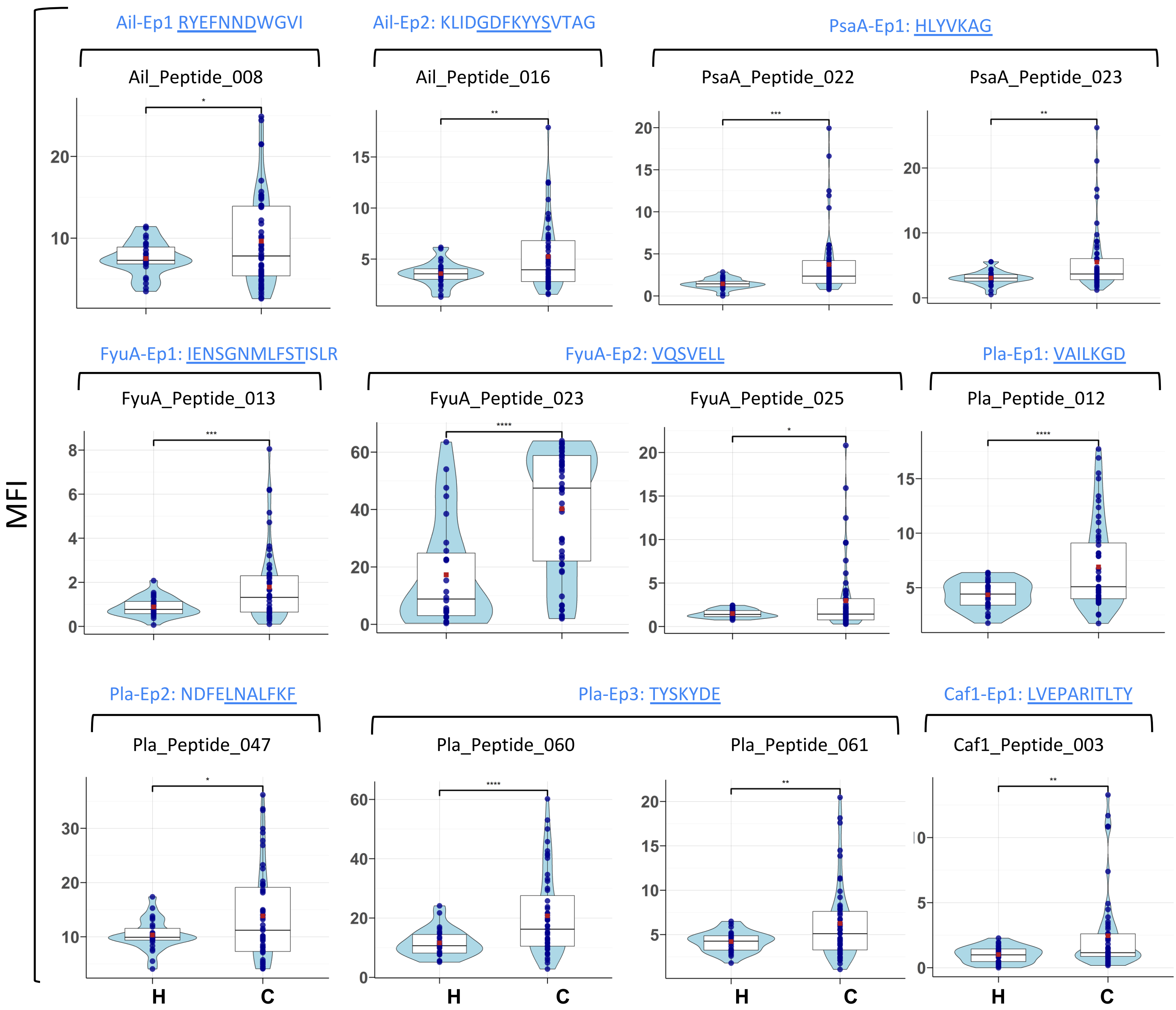

Supplementary Figure S3. Boxplots of IgG signals for the 32 peptides identified in humans.
Each panel shows the comparison of IgG responses against each peptide in the Healthy (n = 26) and Convalescent (n = 51) groups. Each dot represents the MFI value from an individual within the group. A Student's t-test was applied treating the groups as un-paired, with a statistical significance of p < 0·05 (*), < 0·005 (**), < 0·0005 (***) and < 0·00005 (****), ns: not significant. Values in the Y axis must be multiplied by 1000. The red dot in each box represents the mean value of each group. The horizontal black bar in the plot represents the median, and the square indicates the interquartile range (IQR; 25th - 75th percentile). The corresponding peptide epitope as presented in Table 2 is provided at the top of each panel. MFI: mean fluorescence intensity. H: Healthy. C: Convalescent. Refer to Table 2 for peptide sequences.

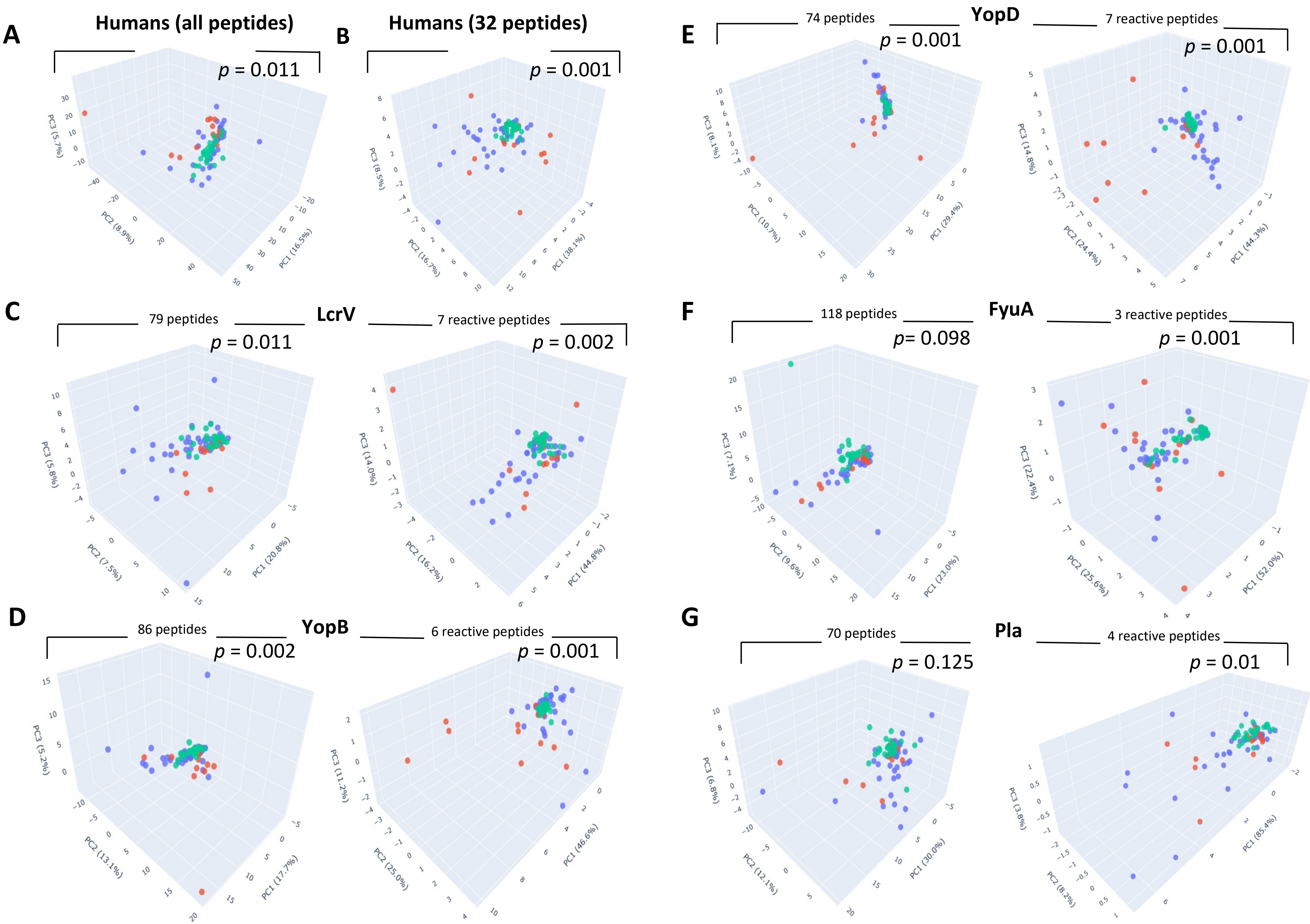
A
Humans (all peptides)
p = 0.011
PC1 (16.5%)
PC2 (8.9%)
PC3 (5.7%)
B
Humans (32 peptides)
p = 0.001
PC1 (38.1%)
PC2 (16.7%)
PC3 (8.5%)
C
79 peptides
p = 0.011
PC1 (20.8%)
PC2 (7.5%)
PC3 (5.8%)
LcrV
7 reactive peptides
p = 0.002
PC1 (44.8%)
PC2 (16.2%)
PC3 (14.0%)
D
86 peptides
p = 0.002
PC1 (17.7%)
PC2 (13.1%)
PC3 (5.2%)
YopB
6 reactive peptides
p = 0.001
PC1 (46.6%)
PC2 (25.0%)
PC3 (11.2%)
E
74 peptides
p = 0.001
PC1 (29.4%)
PC2 (10.7%)
PC3 (8.1%)
YopD
7 reactive peptides
p = 0.001
PC1 (44.3%)
PC2 (24.4%)
PC3 (14.8%)
F
118 peptides
p= 0.098
PC1 (23.0%)
PC2 (9.6%)
PC3 (7.1%)
FyuA
3 reactive peptides
p = 0.001
PC1 (52.0%)
PC2 (25.6%)
PC3 (22.4%)
G
70 peptides
p = 0.125
PC1 (30.0%)
PC2 (12.1%)
PC3 (6.8%)
Pla
4 reactive peptides
p = 0.01
PC1 (85.4%)
PC2 (8.2%)
PC3 (3.8%)

Figure S4

Supplementary Figure S4. Principal component analysis (PCA) of the reactive peptides in humans.

A) Principal Component Analysis with all MFI values from all 1,079 peptides tested.

B) PCA analysis with only the 32 peptides (from 8 antigens) identified as reactive. (C-G) PCAs comparing the complete set of peptides per antigen (right) versus the peptides identified as reactive for each antigen (left). The analysis was performed, when possible, with at least two reactive peptides per antigen. For all the PCAs, the dots represent single individuals in the study and are grouped as follows: green colour for healthy, blue colour for bubonic plague and red for colour for pneumonic plague. The p values obtained by the PERMANOVA test measure the degree of group discrimination between healthy (n = 26), bubonic (n = 37), and pneumonic (n = 14) infected individuals. with significance defined as $p < 0{\cdot}05$.

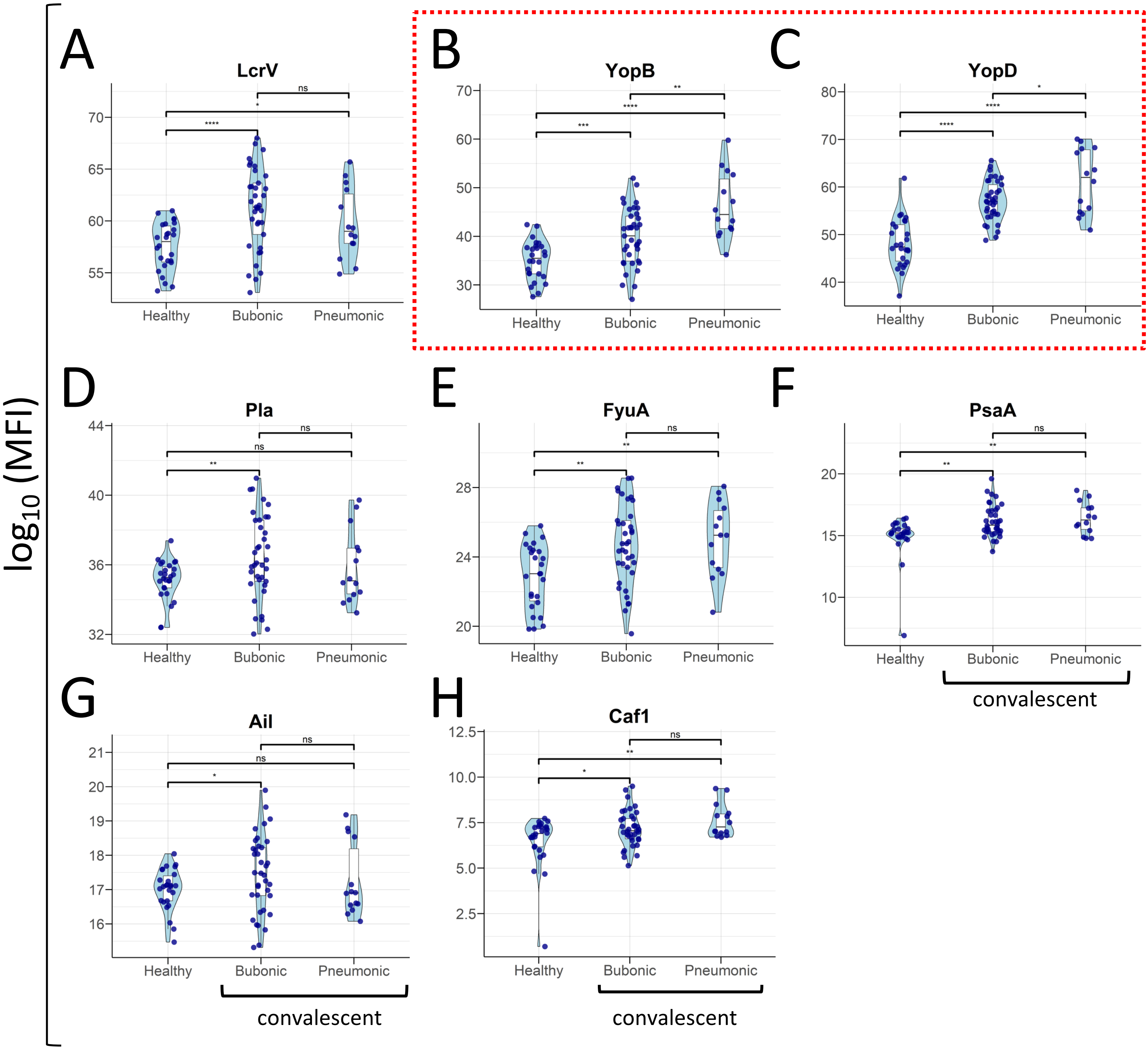

Supplementary Figure S5. Boxplots showing human IgG responses to the eight *Y. pestis* proteins for which reactive peptides were identified.

The antigens with reactive peptides shown in Table 2 are LcrV, YopD, YopB, Pla, FyuA, PsaA, Ail, and Caf1 (A-H). Each panel shows the three human groups, Healthy (n = 26), and Convalescent with either bubonic (n = 37) or pneumonic (n = 14) plague. Each data point represents, for a given individual, the sum of log10-transformed mean fluorescence intensity $\log_{10}$(MFI) of all the peptides found positive in humans: LcrV (n = 7), YopD (n = 7), YopB (n = 6), Pla (n = 4), FyuA (n = 3), PsaA (n = 2), Ail (n = 2), and Caf1 (n = 1) (peptides of Table 2). Statistical analysis between the group pairs was performed using Student's t-test and were treated as un-paired. Significance levels are denoted as follows: $p < 0{\cdot}05$ (*), $p < 0{\cdot}005$ (**), $p < 0{\cdot}0005$ (***), and $p < 0{\cdot}00005$ (****); ns: not significant. The red dotted line frames the antigens YopB and YopD in panels B and C, which showed a statistically significant difference between patients with bubonic and pneumonic plague. The black bar inside each plot represents the median and the bar ends are the 25th and 75th percentiles indicating the interquartile ranges (IQR). The number in parentheses indicates the number of peptides analysed for each protein (n). MFI, mean fluorescence intensity.

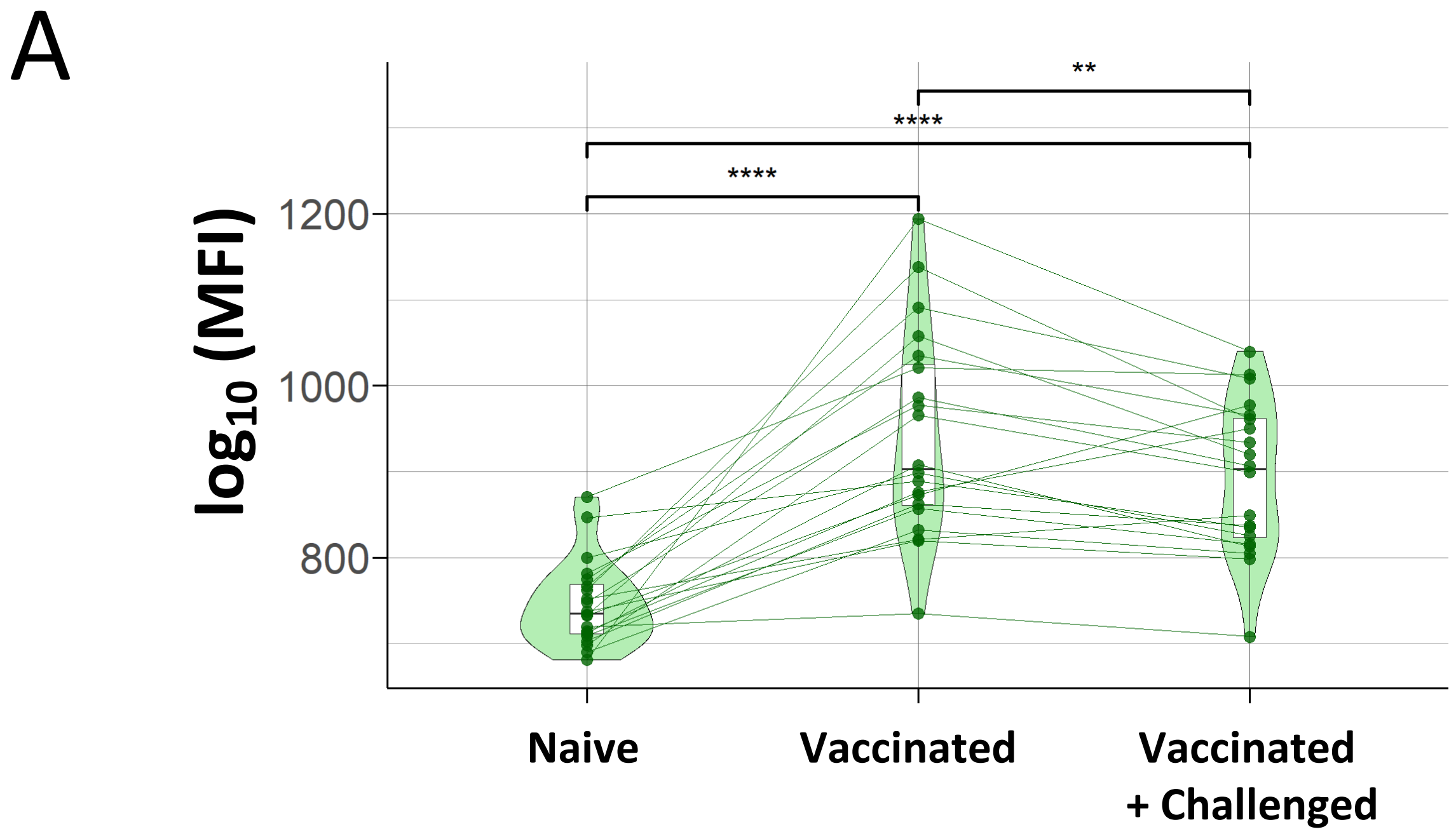

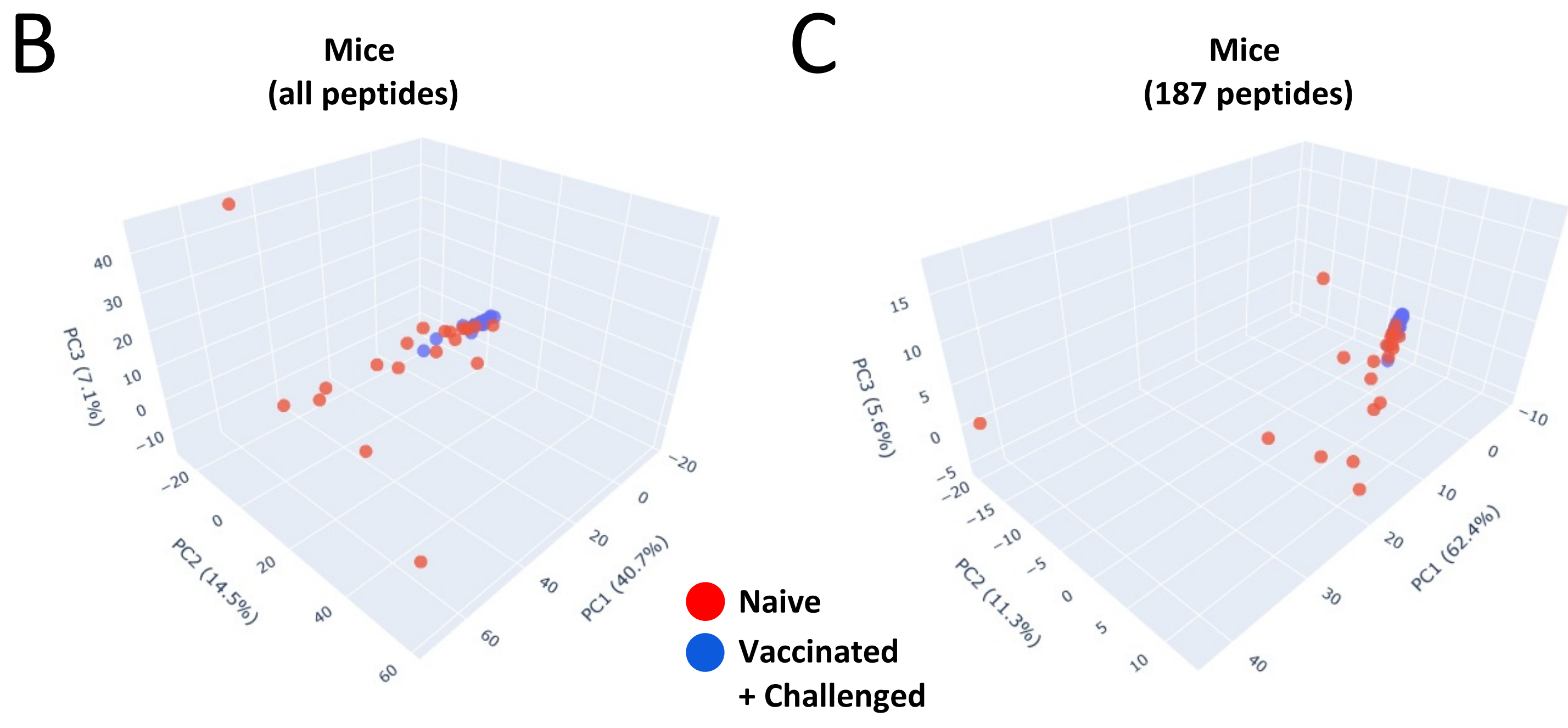

Supplementary Figure S6. IgG responses of mice to *Y. pestis* peptide antigens in the peptide array.
A) Comparison of IgG responses in the peptide array to *Y. pestis* antigens in the mouse model of vaccination and challenge. A total of 20 mice were sampled at three different time points, thus corresponding to 3 groups: 7 days before vaccination (naïve group), 21 days post vaccination (vaccinated group), and 30 days post challenge (vaccinated + challenge group). The graph shows boxplots presenting each mouse (single dot) in the three different groups, displaying per single mouse the sum of $\log_{10}$(MFI) of the 292 linear reactive peptides (coming from the 18 tested antigens) identified in the analysis naïve vs vaccinated. The horizontal black bar in the box plot represents the median of MFI signals in the corresponding group, and the square indicates the interquartile range (IQR; 25th – 75th percentile). The *p* values resulted from a paired Student's *t*-test between the groups *p* value < 0·05 (**), and < 0·0005 (****). B) PCA analysis performed with mice dataset with all the peptides tested. C) PCA analysis with only the MFI values of 187 peptides found as reactive in the comparison between naïve vs vaccinated + challenged. The naïve group is shown in blue (n = 20); and the vaccinated + challenged is shown in red (n = 20). MFI: mean fluorescence intensity.

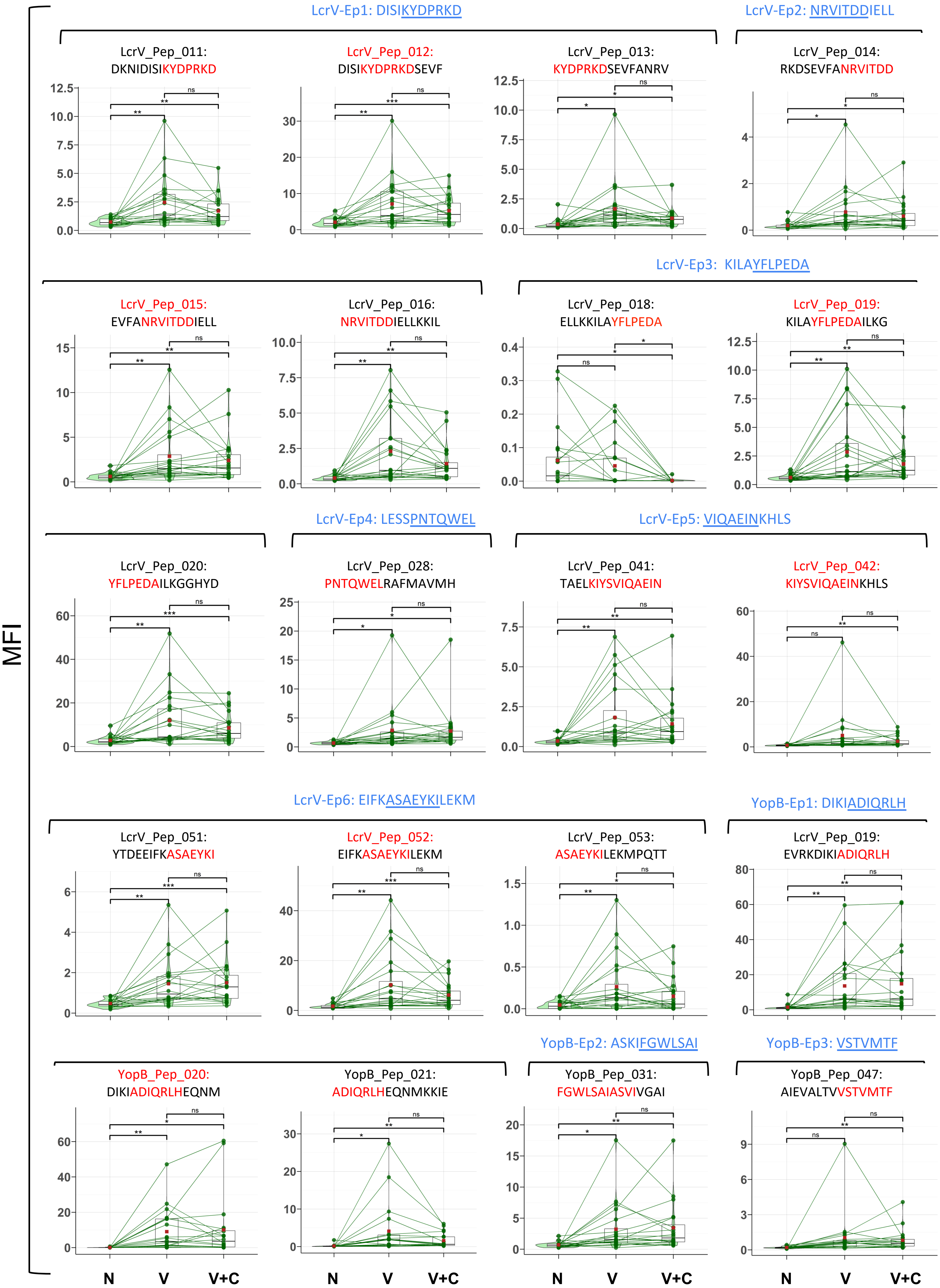

MFI
LcrV-Ep1: DISIKYDPRKD
LcrV-Ep2: NRVITDDIELL
LcrV_Pep_011: DKNIDISIKYDPRKD
LcrV_Pep_012: DISIKYDPRKDSEVF
LcrV_Pep_013: KYDPRKDSEVFANRV
LcrV_Pep_014: RKDSEVFANRVITDD
LcrV-Ep3: KILAYFLPEDA
LcrV_Pep_015: EVFANRVITDDIELL
LcrV_Pep_016: NRVITDDIELLKKIL
LcrV_Pep_018: ELLKKILAYFLPEDA
LcrV_Pep_019: KILAYFLPEDAILKG
LcrV-Ep4: LESSPNTQWEL
LcrV-Ep5: VIQAEINKHLS
LcrV_Pep_020: YFLPEDAILKGGHYD
LcrV_Pep_028: PNTQWELRAFMAVMH
LcrV_Pep_041: TAELKIYSVIQAEIN
LcrV_Pep_042: KIYSVIQAEINKHLS
LcrV-Ep6: EIFKASAEYKILEKM
YopB-Ep1: DIKIADIQRLH
LcrV_Pep_051: YTDEEIFKASAEYKI
LcrV_Pep_052: EIFKASAEYKILEKM
LcrV_Pep_053: ASAEYKILEKMPQTT
LcrV_Pep_019: EVRKDIKIADIQRLH
YopB-Ep2: ASKIFGWLSAI
YopB-Ep3: VSTVMTF
YopB_Pep_020: DIKIADIQRLHEQNM
YopB_Pep_021: ADIQRLHEQNMKKIE
YopB_Pep_031: FGWLSAIASVIVGAI
YopB_Pep_047: AIEVALTVVSTVMTF
N
V
V+C

Figure S7

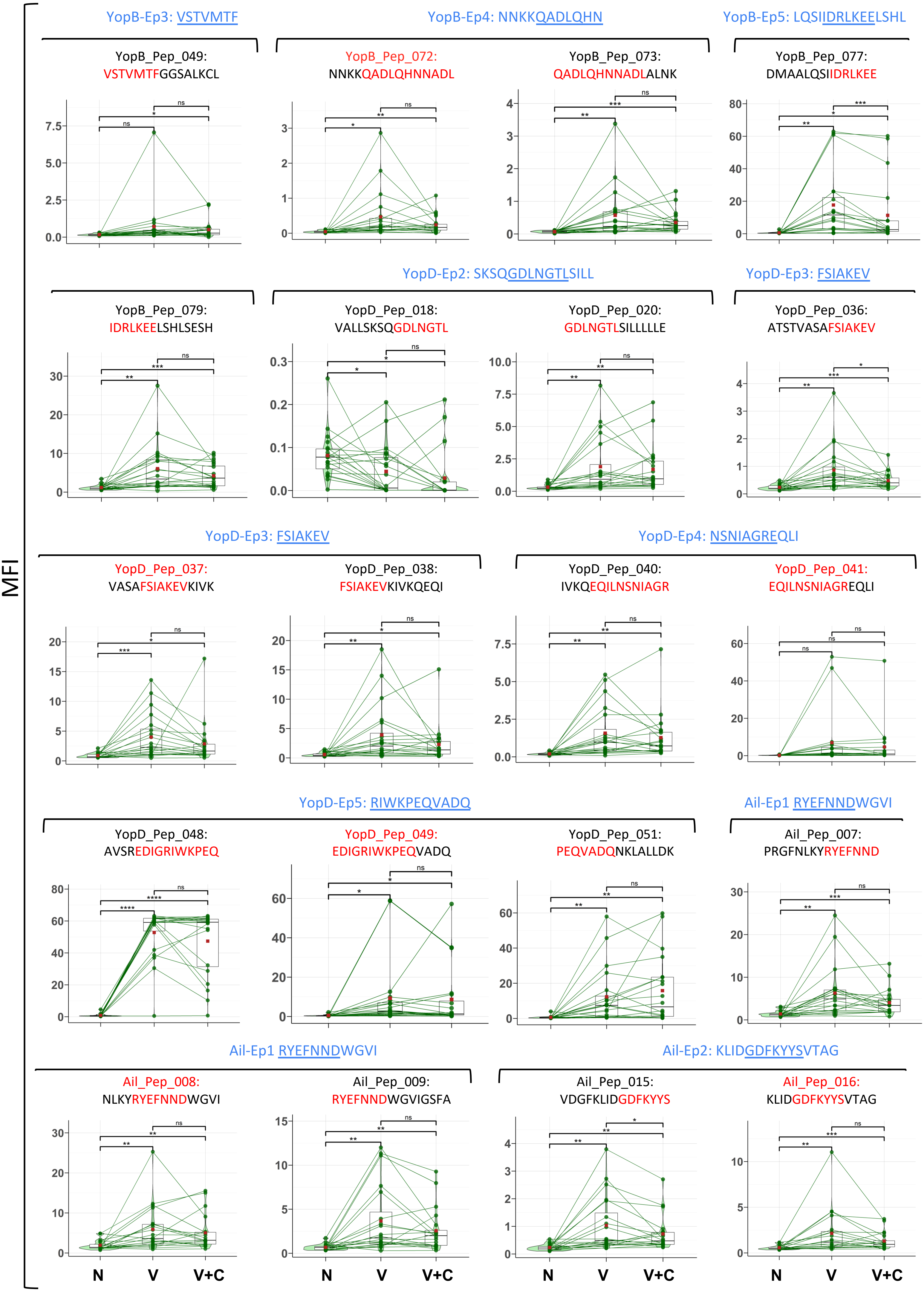

MFI
YopB-Ep3: VSTVMTF
YopB-Ep4: NNKKQADLQHN
YopB-Ep5: LQSIIDRLKEELSHL
YopB_Pep_049: VSTVMTFGGSALKCL
YopB_Pep_072: NNKKQADLQHNNADL
YopB_Pep_073: QADLQHNNADLALNK
YopB_Pep_077: DMAALQSIIDRLKEE
YopD-Ep2: SKSQGDLNGTLSILL
YopD-Ep3: FSIAKEV
YopB_Pep_079: IDRLKEELSHLSESH
YopD_Pep_018: VALLSKSQGDLNGTL
YopD_Pep_020: GDLNGTLSILLLLLE
YopD_Pep_036: ATSTVASAFSIAKEV
YopD-Ep3: FSIAKEV
YopD-Ep4: NSNIAGREQLI
YopD_Pep_037: VASAFSIAKEVKIVK
YopD_Pep_038: FSIAKEVKIVKQEQI
YopD_Pep_040: IVKQEQILNSNIAGR
YopD_Pep_041: EQILNSNIAGREQLI
YopD-Ep5: RIWKPEQVADQ
Ail-Ep1 RYEFNNDWGVI
YopD_Pep_048: AVSREDIGRIWKPEQ
YopD_Pep_049: EDIGRIWKPEQVADQ
YopD_Pep_051: PEQVADQNKLALLDK
Ail_Pep_007: PRGFNLKYRYEFNND
Ail-Ep1 RYEFNNDWGVI
Ail-Ep2: KLIDGDFKYYSVTAG
Ail_Pep_008: NLKYRYEFNNDWGVI
Ail_Pep_009: RYEFNNDWGVIGSFA
Ail_Pep_015: VDGFKLIDGDFKYYS
Ail_Pep_016: KLIDGDFKYYSVTAG
N
V
V+C

Figure S7

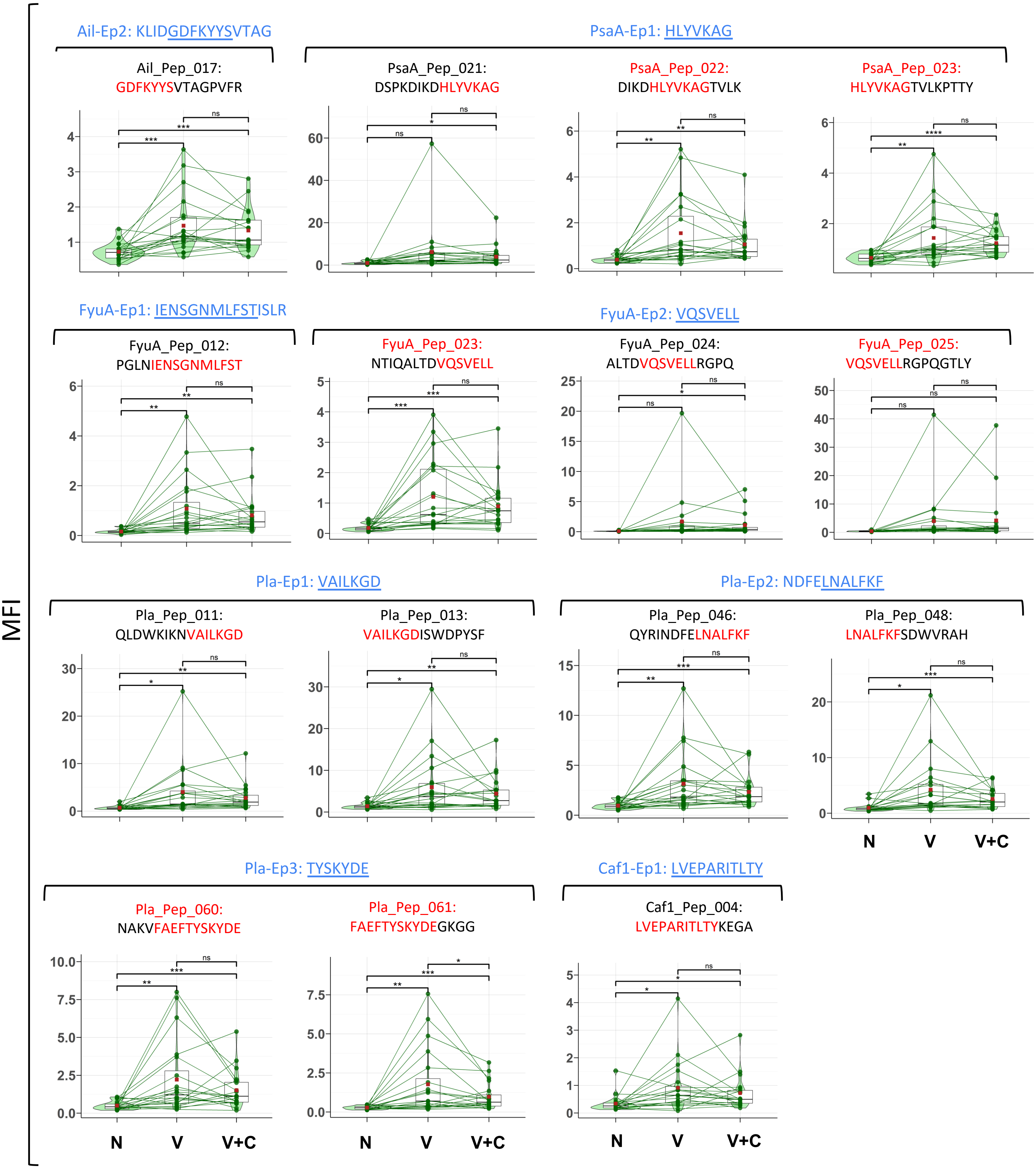

Supplementary Figure S7. Boxplots of mouse IgG responses to peptides overlapping with the 32 peptides identified in humans. The figure shows individual peptides in the mouse dataset that were used to map the peptide epitopes taking as reference the list of 32 reactive peptides identified in humans (peptide names in red). Panels are grouped according to the corresponding peptide epitopes, resulting in a total of 25 epitopes (epitope sequences shown in blue) out of the 27 epitopes found in humans. Indeed, no reactive peptides were identified in mice for the peptide epitopes LcrV-Ep7 and YopD-Ep1. The respective overlapping peptides, when found to be reactive by the *t*-test, $p < 0{\cdot}05$ in the comparison between naïve vs vaccinated + challenged are shown with their sequences highlighted in red to indicate the overlapping sequence identified. Refer to Table S6 for details of peptide sequences and peptide epitopes. Each panel shows the antibody responses for each mouse group (naïve, vaccinated, and vaccinated + challenged) with each dot representing the MFI value for an individual mouse ($n = 20$ per group). Comparison of reactivity between groups were performed using paired Student's *t*-test with statistical significance defined as $p < 0{\cdot}05$ (*), $< 0{\cdot}005$ (**), $< 0{\cdot}0005$ (***) and $< 0{\cdot}00005$ (****), ns: not significant. Values on the Y-axis should be multiplied by 1000. The red square represents the mean value for each group in each panel. The horizontal black bar represents the median. and the square indicates the interquartile range (IQR; 25th - 75th percentile). MFI: mean fluorescence intensity. N: naïve. V: vaccinated. V+C: vaccinated + challenged.

LcrV

2
1 MIRAYEQNPQHFIEDLEKVRVEQLTGHGSSVLEELVQLVKDKNIDISIKYDPRKDSEVFANRVITDDIELLKKILAYFLPEDAILKGGHYD 91

LcrV-Ep1—DISIKYDPRKD

LcrV-Ep2—NRVITDDIELL KILAYFLPEDA—LcrV-Ep3

MIRAYEQNPQHFIEDLEKVRVEQLTGHGSS—(Pullen *et al.*, 1998)

92 NQLQNGIKRVKEFLESSPNTQWELRAFMAVMHFSLTADRIDDDILKVIVDSMNHHGDARSKLREELAELTAELKIYSVIQAEINKHLSSSG 182

LcrV-Ep4—LESSPNTQWEL

135

LcrV-Ep5—VIQAEINKHLS

183 TINIHDKSINLMDKNLYGYTDEEIFKASAEYKILEKMPQTTIQVDGSEKKIVSIKDFLGSENKRTGALGNLKNSYSYNKDNNELSHFATTC 273

LcrV-Ep6—EIFKASAEYKILEKM

KNLYGYTDEEIFKASAEYKILEKMPQTTIQ — P. E. for mAb BA5 (Quenee *et al.*, 2010)

SDKSRPLNDLVSQKTTQLSDITSRFNSAIEALNRFIQKYDSVMQRLLDDTSGK

275 326

LcrV-Ep7—AIEALNRFIQKYDSV

-Motin *et al.*, 1994, region containing P.E.: XXXX.
-Hill *et al.*, 1997: minor and major epitopes. Major epitope generated the mAb7.3.
-Hill *et al.*, 2009: N255 essential for mAb7.3 activity.

YopB

YopB-Ep1 YopB-Ep3 YopB-Ep2

~~MSALITHDRSTPVTGSLLPYVETPAPAPLQTQQVAGELKDKNGGVS~~SQGVQLPAPlaVVASQVTEGQQQEVTKLLESVTRGAAGSQLISNY
VSVLTKFTLASPDTFEIELGKLVSNLEEVRKDIKIADIQRLHEQNMKKIEENQEKIKETEENAKQVKKSGIASKIFGWLSAIASVIVGAIM
VASGVGAVAGAMMVASGVIGMANMAVKQAAEDGLISQEAMKILGPILTAIEVALTVVSTVMTFGGSALKCLANIGAKLGANTASLVAKGAE
FSAKVAQISTGISNTVGSAVTKLGGSFAGLTMSHAIRTGSQATQVAVGVGSITQTINNKKQADLQHNGNADLALNKADMAALQSIIDRLKE
ELSHLSESHQQVMELIFQMINAKGDMLHNLAGRPHTV

YopB-Ep4 YopB-Ep5

YopD

YopD-Ep1 YopD-Ep2

MTINIKTDSPIITTGSQLDAITTETVKQSGEIKKTEDTRHEAQAIKSSEASLSRSQVPELIKPSQGINVALLSKSQGDLNGTLSILLLLE
LARKAREMGLQQRDIENKATITAQKEQVAEMVSGAKLMIAMAVVSGIMAATSTVASAFSIAKEVKIVKQEQILNSNIAGREQLIDTKMQQM
SNIGDKAVSREDIGRIWKPEQVADQNKLALLDKEFRMTDSKANAFNAATQPLGQMANSAIQVHQGYSQAEVKEKEVNASIAANEKQKAEEA
MNYNDNFMKDVLRLIEQYVSSHTHAMKAAFGV

YopD-Ep5 YopD-Ep3 YopD-Ep6 YopD-Ep4

Ail

Ail-Ep1 Ail-Ep2

~~MNKTLLVSSLIACLSIASVNVYAE~~GESSISIGYAQSRVKEDGYKLDKNPRGFNLKYRYEFNNDWGVIGSFAQTRRGFEESVDGFKLIDGDF
RRGFEESVDGFK
KYYSVTAGPVFRINEYVSLYGLLGAGHGKAKFSSIFGQSESRSKTSLAYGAGLQFNPHPNFVIDASYEYSKLDDVKVGTWMLGAGYRF

(EL2-Kolodziejeck *et al.*, 2023)

PsaA

~~MKMKCFAKNALAVTTLMIAACGMANASTVINSKDVSGEVTVKQG~~NTFHVDFAPNTGEIFAGKQPGDVTMFTLTMGDTAPHGGWRLIPTGDS
KGGYMISADGDYVGLYSYMMSWVGIDNNWYINDDSPKDIKDHLYVKAGTVLKPTTYKFTGRVEEYVF

PsaA-Ep1

FyuA

FyuA-Ep2 FyuA-Ep1

~~MKMTRLYPlaLGGLLLPAIANAQTSQQDESTLVV~~TASKQSSRSASANNVSSTVVSAPELSDAGVTASDKLPRVLPGLNIENSGNMLFSTIS
LRGVSSAQDFYNPAVTLYVDGVPQLSTNTIQALTDVQSVELLRGPQGTLYGKSAQGGIINIVTQQPD~~STPRGYIEGGVSSRDSYRSKFNLS~~
~~GPIQDGLLYGSVTL~~LRQVDDGDMINPATGSDDLGGTRASIGNV~~KLRLAPDDQPWEMG~~FAASRECTRATQDAYVGWNDIKGRKLSISDGSPD
PYMRRCTDSQTLS~~GKYTTDDWVF~~NLISAWQQQHYSRTFPSGSLIVNMPQRWNQDVQE~~LRAATLGDARTVDM~~VFGLYRQNTREKLNSAYDMP
TMPYLSSTGYTTAETLAA~~YSDLTWHLTDRFDIG~~GGVRFSHDKSSTQYHGSMLGNPFGDQGKSNDDQVLGQ~~LSAGYMLTDDWRVY~~TRVAQGY
KPSGYNIVPTAGLDAKPFVAEKSINYEL~~GTRYETADVTLQA~~ATFYTHTKDMQLYSGPVRMQTLSNAGKADATGVELEAKWRFAPGWSWDIN
GNVIRSEFTNDSELYHGNRVPFVPRYGAGS~~SVNGVIDTRYGALM~~PRLAVNLVGPHYFDGDNQLRQGTYATLDS~~SLGWQATERMNIS~~VYVDN
LFDRRYRTYGYMNGSSAVAQVNMGRTVGIN~~TRIDFF~~

Pla

Pla-Ep1

~~MKKSSIVATIITILSGSANAA~~SSQLIPNISPDSFTVAASTGMLSGKSHEMLYDAETGRKISQLDWKIKNVAILKGDISWDPYSFLTLNARG
WTSLASGSGNMDDYDWMNENQSEWTDHSSHPATNVNHANEYDLNVKGWLLQDENYKAGITAGYQETRFSWTATGGSYSYNNGAYTGNFPKG
VRVIGYNQRFSMPYIGLAGQYRINDFELNALFKFSDWVRAHDNDEHYMRDLTFREKTSGSRYYGTVINAGYYVTPNAKVFAEFTYSKYDEG
KGGTQTIDKNSGDSVSIGGDAAGISNKNYTVTAGLQYRF

(Feodorova *et al.*, 2018) — TPNAKVFAEFTYSKY

Pla-Ep2 Pla-Ep3

Caf1

Caf1-Ep1

~~MKKISSVIAIALFGTIATANA~~ADLTASTTATATLVEPARITLTYKEGAPITIMDNGNIDTELLVGTLTLGGYKTGTTSTSVNFTDAAGDPM
YLTFTSQDGNNHQFTTKVIGKDSRDFDISPKVNGENLVGDDVVLATGSQDFFVRSIGSKGGKLAAGKYTDAVTVTVSNQ

FFVRSIGSKGGKLAAGKYTDAVTVTV—(Sabhnani and Rao, 2000)

Figure S8

Supplementary Figure S8. Sequence alignment and mapping of the 27 peptides epitopes onto their corresponding *Y. pestis* protein sequences.

The barred amino acid residues in the protein sequences correspond to peptide regions that were removed and not included in the epitope mapping analysis. Epitopes identified in the present study are highlighted in blue. For LcrV, a detailed representation also includes previously described protective epitope-containing regions (Pullen *et al*., 1998) and a protective epitope (P.E.) corresponding to the binding site of the protective monoclonal antibody BA5 (Quenee *et al.*, 2010). Regions described as the major and minor epitopes characterised by Hill *et al*., 1997 are also shown; the major epitope is the target of the protective monoclonal antibody mAb 7.3. In yellow the amino acid N255 necessary for protective activity of mAb7.3 is shown (Hill *et al*., 2009). For Ail, the extracellular loop 2 (EL2) is highlighted in red that has been reported to be involved in cell adhesion and ECM binding (Kolodziejeck *et al*., 2023). ECM: extracellular matrix. For Pla, the B cell epitope identified in a previous study is shown in red colour (Feodorova *et al*., 2018). For Caf1, the immunodominant epitope reported by Sabhnani and Rao (2000) is shown. For the full bibliographic details of the references cited in this figure legend, please refer to the reference list in the main manuscript.

A

1,079 peptides

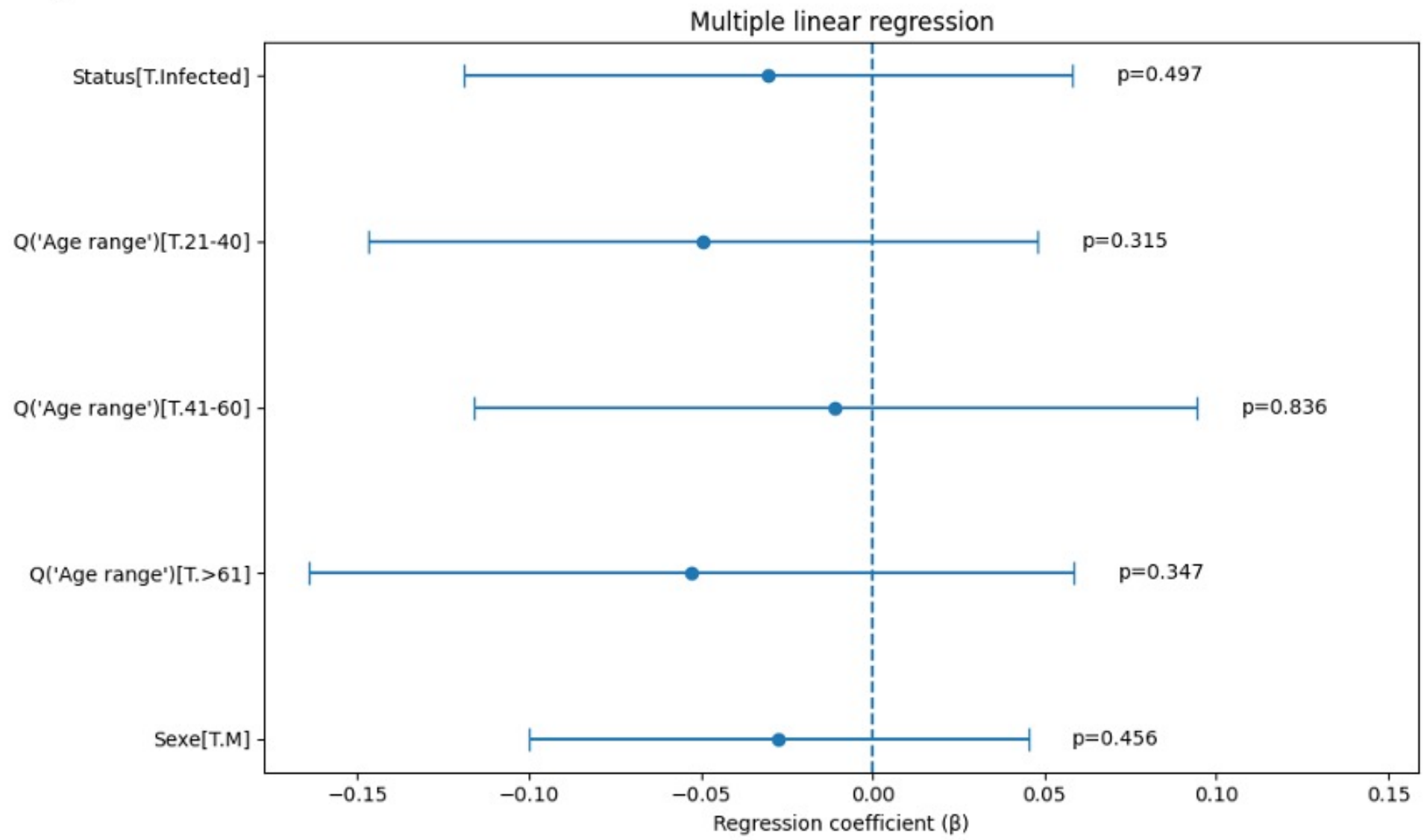

OLS Regression Results

Dep. Variable:	PeptideScore	R-squared:	0.037
Model:	OLS	Adj. R-squared:	-0.031
Method:	Least Squares	F-statistic:	0.5440
Date:	Fri, 22 May 2026	Prob (F-statistic):	0.742
Time:	18:04:52	Log-Likelihood:	38.118
No. Observations:	77	AIC:	-64.24
Df Residuals:	71	BIC:	-50.17
Df Model:	5		
Covariance Type:	nonrobust		

	coef	std err	t	P>|t|	[0.025	0.975]
Intercept	2.6807	0.048	56.279	0.000	2.586	2.776
Status[T.Infected]	-0.0303	0.044	-0.682	0.497	-0.119	0.058
Q('Age range')[T.21-40]	-0.0494	0.049	-1.013	0.315	-0.147	0.048
Q('Age range')[T.41-60]	-0.0109	0.053	-0.207	0.836	-0.116	0.094
Q('Age range')[T.>61]	-0.0528	0.056	-0.947	0.347	-0.164	0.058
Sexe[T.M]	-0.0273	0.036	-0.750	0.456	-0.100	0.045

Omnibus:	0.169	Durbin-Watson:	1.386
Prob(Omnibus):	0.919	Jarque-Bera (JB):	0.344
Skew:	0.072	Prob(JB):	0.842
Kurtosis:	2.706	Cond. No.	6.41

B

32 reactive peptides in humans

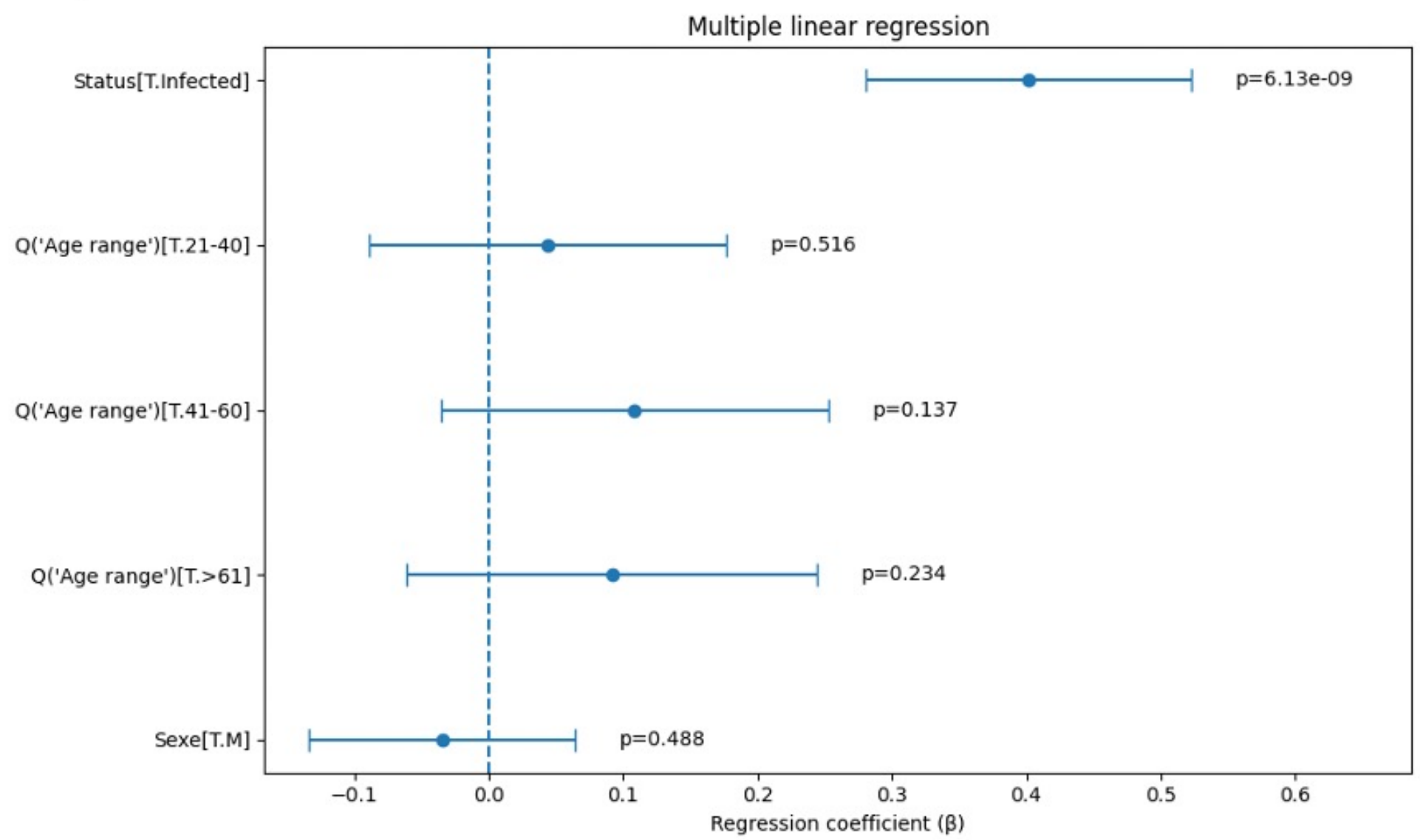

OLS Regression Results

Dep. Variable:	PeptideScore	R-squared:	0.413
Model:	OLS	Adj. R-squared:	0.372
Method:	Least Squares	F-statistic:	9.993
Date:	Fri, 22 May 2026	Prob (F-statistic):	2.87e-07
Time:	18:06:28	Log-Likelihood:	13.961
No. Observations:	77	AIC:	-15.92
Df Residuals:	71	BIC:	-1.859
Df Model:	5		
Covariance Type:	nonrobust		

	coef	std err	t	P>|t|	[0.025	0.975]
Intercept	3.1534	0.065	48.377	0.000	3.023	3.283
Status[T.Infected]	0.4013	0.061	6.607	0.000	0.280	0.522
Q('Age range')[T.21-40]	0.0436	0.067	0.653	0.516	-0.089	0.177
Q('Age range')[T.41-60]	0.1086	0.072	1.505	0.137	-0.035	0.253
Q('Age range')[T.>61]	0.0916	0.076	1.200	0.234	-0.061	0.244
Sexe[T.M]	-0.0347	0.050	-0.696	0.488	-0.134	0.065

Omnibus:	1.383	Durbin-Watson:	1.399
Prob(Omnibus):	0.501	Jarque-Bera (JB):	1.223
Skew:	0.306	Prob(JB):	0.543
Kurtosis:	2.912	Cond. No.	6.41

Figure S9

Supplementary Figure S9. Multiple linear regression analysis assessing potential confounding variables.
Forest plots show the regression coefficients (β) and 95% confidence intervals for predictors included in the multivariable linear regression model: *Y. pestis* exposure status (healthy vs. convalescent), sex, and age group. The mean $\log_{10}$-transformed MFI value for each peptide was used as the dependent variable, whereas *Y. pestis* exposure status, sex, and age group were included as independent variables.
(A) Multivariable linear regression performed using all 1,079 peptides.
(B) Multivariable linear regression performed using the 32 reactive peptides listed in Table 2.
A significant association with peptide reactivity was observed only for *Y. pestis* exposure status (convalescent vs. healthy), whereas neither age group nor sex showed a significant effect in either the complete peptide dataset or the subset of 32 reactive peptides. The accompanying tables report the regression coefficients (β), standard errors (SE), and *p* values for each predictor. Complete results of the ordinary least squares (OLS) regression analyses, including regression coefficients, 95% confidence intervals, and *p* values, are provided in the OLS regression summary tables associated with each forest plot. Abbreviations: MFI. mean fluorescence intensity; OLS, ordinary least squares.

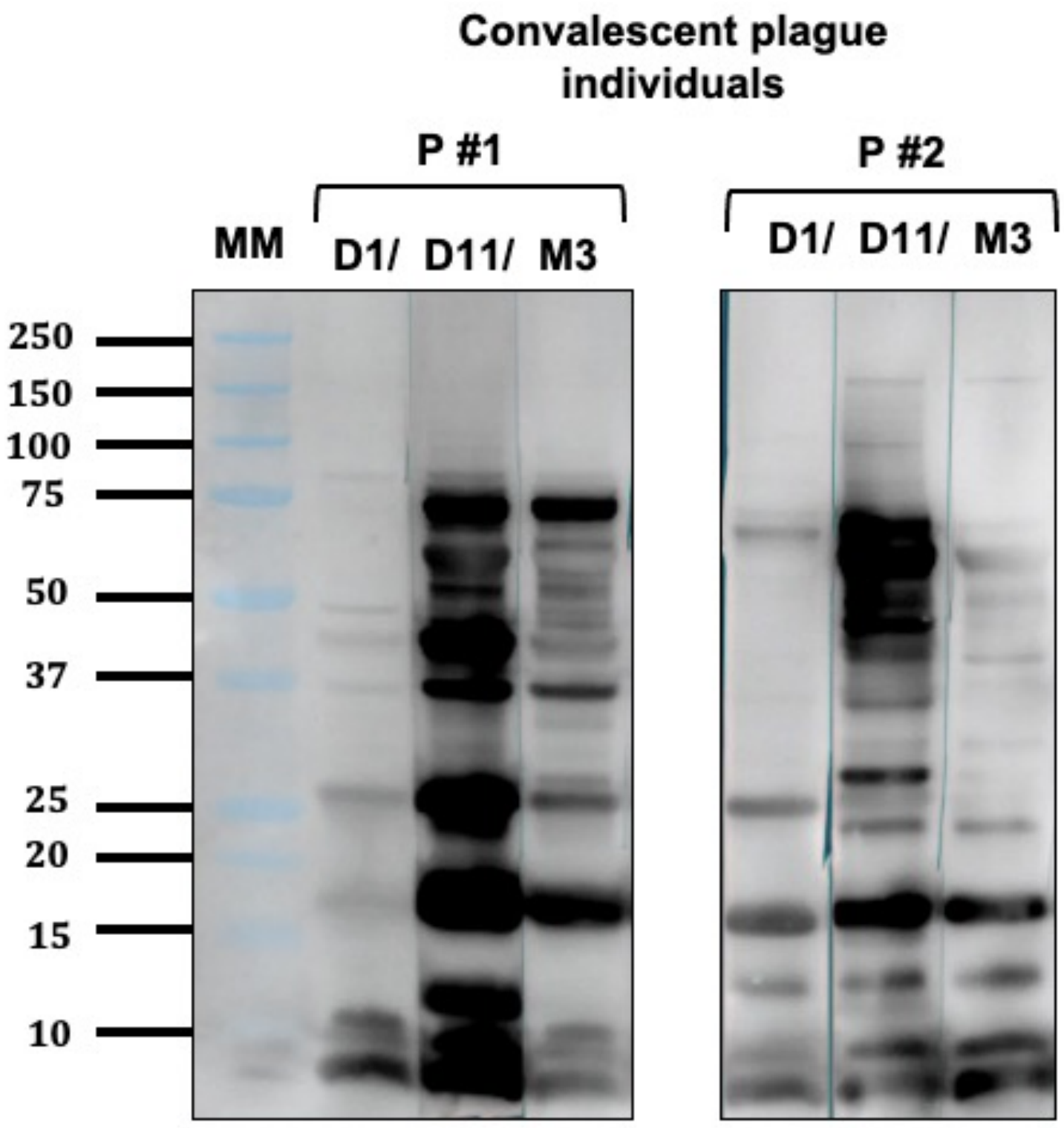

Figure S10. Western blot of *Y. pestis* proteins with sera from patients with plague collected on day 11 and Month 3 post-infection.

Whole cell lysates of *Y. pestis* CO92 strain were grown in low calcium conditions and at 37 ºC. Protein lysates were prepared and run onto SDS-PAGE and transfer to PVDF membranes (iBlot 2 Dry Blotting System with iBlot 2 PVDF Transfer Stacks; Invitrogen, Thermo Fisher Scientific, Cat# IB24002). Strip of lanes were cut and incubated separately with plasma from two patients (1:50) at three different time points (day 1, day 11, and month 3 post declaration of plague disease). The blot was revealed using secondary antibody anti human IgG1 Fc specific (monoclonal antibody clone HP-6001, Sigma #I9388, RRID:AB_260314) (1:10,000). The molecular marker (MM) is Precision Plus Protein™ All Blue Prestained (Biorad #1610373). P = patient. D = day. M = month.
